# Zinc supplementation for acute and persistent watery diarrhoea in children: A systematic review and meta-analysis

**DOI:** 10.7189/jogh.14.04212

**Published:** 2024-12-06

**Authors:** Ayesha Arshad Ali, Syeda Kanza Naqvi, Zain Hasnain, Mustafa Bin Ali Zubairi, Ashraf Sharif, Rehana Abdus Salam, Sajid Soofi, Shabina Ariff, Yasir Bin Nisar, Jai K Das

**Affiliations:** 1 Institute for Global Health and Development, Aga Khan University, Karachi, Pakistan; 2 University Library, Aga Khan University, Karachi, Pakistan; 3 The Daffodil Centre, The University of Sydney, a joint venture with Cancer Council NSW, Sydney, New South Wales, Australia; 4 Centre of Excellence in Women and Child Health, Aga Khan University, Karachi, Pakistan; 5 Department of Pediatrics & Child Health, Aga Khan University, Karachi, Pakistan; 6 Department of Maternal, Newborn, Child, and Adolescent Health and Ageing, World Health Organization, Geneva, Switzerland; 7 Division of Women and Child Health, Aga Khan University, Karachi, Pakistan

## Abstract

**Background:**

Zinc is a micronutrient that plays a role in immune system strengthening and regulation of intestinal epithelial cells, and can reduce the duration and severity of diarrhoea. We conducted a systematic review of randomised controlled trials (RCTs) to assess the effectiveness of zinc compared to no zinc for the management of acute and persistent diarrhoea in children.

**Methods:**

We searched PubMed, the Cochrane Library, Scopus, CINAHL, ClinicalTrials.gov, and World Health Organization (WHO) International Clinical Trials Registry Platform from inception until 31 July 2023 for studies published from year 2000 onwards that assessed the use of zinc in the management of acute and persistent diarrhoea in children aged less than 10 years. We conducted the meta-analysis in Cochrane’s RevMan software, determined risk of bias in individual studies using the Risk of Bias 2 (RoB 2) tool, and assessed the quality of evidence through the Grading of Recommendations Assessment, Development and Evaluation (GRADE) approach. This review was commissioned by the WHO for revision of their guidelines for childhood diarrhoea management.

**Results:**

We included 38 RCTs in this systematic review. Our findings suggest that, in children with acute diarrhoea, zinc supplementation resulted in a greater proportion of children who recovered from diarrhoea at last follow-up (risk ratio (RR) = 1.07; 95% confidence interval (CI) = 1.03, 1.1; moderate certainty of evidence) and a reduction in the duration of diarrhoea (mean difference (MD) = −13.27 hours; 95% CI = −17.66, −8.89; moderate certainty of evidence) when compared to placebo. A significant number of children in the zinc group compared to placebo experienced vomiting (RR = 1.46; 95% CI = 1.22, 1.76; moderate certainty of evidence), however, there were few vomiting episodes in low-dose zinc group compared to high-dose (RR = 0.80; 95% CI = 0.72, 0.89; moderate certainty of evidence). In children with persistent diarrhoea, zinc supplementation led to a greater proportion of children who recovered from diarrhoea (RR = 1.75; 95% CI = 1.34, 2.30; low certainty of evidence). The low certainty of evidence ratings were mostly due to high heterogeneity among the studies.

**Conclusions:**

Zinc should continue to be recommended in children under the age of 10 years with acute or persistent diarrhoea, but moderate certainty of evidence concludes that the dose of zinc should be reduced. However, further multi-country randomised clinical trials are required with a direct comparison to assess the appropriate dosage, duration and adverse effects.

**Registration:**

PROSPERO: CRD42023439028.

Diarrhoea is defined by the World Health Organization (WHO) as the passage of three or more loose or liquid stools per day or as an increase in stool frequency [1,2]. It is categorised into three types: acute watery diarrhoea, defined as diarrhoea lasting for several hours or days but less than 14 days; acute bloody diarrhoea, also known as dysentery; and persistent diarrhoea, defined as diarrhoea lasting 14 days or longer [1,3]. Typically, diarrhoeal diseases are transmitted through contaminated food or beverages and can be caused by viruses, bacteria, or gastrointestinal parasites (worms). The incubation period varies, ranging from several hours to days, contingent upon the underlying aetiology of the disease [4].

In the 1990s, the WHO, in partnership with the United Nations International Children's Emergency Fund (UNICEF), developed the Integrated Management of Childhood Illness (IMCI) approach to reduce morbidity and mortality associated with diarrhoea [5]. Various measures implemented in later years have led to a decline in diarrhoea-related mortality, but the prevalence of diarrhoeal disease still remains significant [6]. According to WHO estimates, diarrhoeal diseases cause an estimated 444 000 deaths of children under the age of five years globally in 2021 which accounted for 9% of all deaths in children under the age of five years [1,7]. Low- and middle-income countries (LMICs) in South Asia and sub-Saharan Africa account for almost 90% of global diarrhoeal deaths in children and bear a significant disease burden [8]. Diarrhoea also leads to long-term consequences, including repeated infections due to weak immunity, malnutrition, growth failure, and cognitive delays [4,6].

The primary task to managing children with acute watery or persistent diarrhoea is preventing dehydration, since diarrhoea can lead to significant water and electrolyte loss (e.g. sodium, chloride, potassium). An important advancement in diarrhoea management was the introduction of the WHO oral rehydration solution (ORS), which has helped reduce morbidity and mortality [9,10]. The use of zinc has further supplemented ORS in these gains [11,12]. The WHO and UNICEF currently recommend prescribing 10–20 mg of oral zinc per day for 14 days to children under five years of age with diarrhoea in available forms of oral zinc, including zinc sulphate, zinc acetate, or zinc gluconate [13,14].

Zinc is a micronutrient and antioxidant that plays a role in cell growth, immune system strengthening, and regulation of intestinal epithelial cells. The literature highlights that zinc deficiency compromises the integrity of the intestinal barrier; consequently, zinc supplementation during diarrhoea can help restore the tight junctions between intestinal cells, reducing the leakage of fluid and electrolytes [15]. Additionally, the role of zinc in boosting the immune system makes it a key part of diarrhoea management, with evidence showing that zinc can reduce the duration of diarrhoea, stool output, and the risk of developing persistent diarrhoea in children [4].

The previous meta-analyses and systematic reviews, alongside current WHO recommendations, support the efficacy of zinc supplementation in managing diarrhoea in children up to five years of age [3]. The WHO has commissioned this review to update its existing guidelines on childhood diarrhoea management. We aimed to synthesise the findings of studies on the effectiveness of oral zinc in children aged up to 10 years with acute watery or persistent diarrhoea and assess the optimum dose, duration, and formulation of zinc supplementation.

## METHODS

### Objective

The objective of this systematic review is to assess the effectiveness of zinc compared to no zinc/placebo for the management of acute watery or persistent diarrhoea in children less than 10 years of age. The protocol for this review was registered in PROSPERO (CRD42023439028). We followed the PRISMA guidelines in reporting our findings [16].

### Inclusion criteria

We included randomised controlled trials (RCTs) that reported the diagnosis of diarrhoea among children aged up to 10 years, provided they were published from the year 2000 onwards in the English language. We excluded case reports, case series, cohort studies, opinions, editorials, commentaries, letters, conference abstracts, reviews or systematic reviews, and studies with external comparison groups (i.e. historical cohort etc.), as well as studies focussing solely on prevention of diarrhoea in children, rather than treatment.

### Outcomes

We defined the outcomes of interest for this review according to the authors of the original studies, as follows: time to recovery; mortality, serious adverse events (including vomiting); and duration of diarrhoea.

### Search strategy

We designed our search strategy per the PICO methodology based on MeSH terms and keywords, but did not restrict it by outcome-related keywords retain a broader search (Tables S1–5 in the **Online Supplementary Document**). We ran the searches in PubMed, CINAHL, the Cochrane Library, ClinicalTrials.gov, the WHO International Clinical Trials Registry Platform, and Scopus until 31 July 2023. We also searched the reference list of all the included studies and relevant systematic reviews for studies not caught by our search. We additionally queried Google Scholar with the title of each included study and screened the first 50 results and their reference lists for potentially relevant studies.

We exported the search results into EndNote, version 20 (Clarivate, London, UK) and uploaded them onto the Covidence platform [17] for deduplication and title, abstract, and full-text screening. The screening itself was conducted independently by at least two authors (ZH, MBAZ, SKN, and AAA) based on pre-determined criteria (**Box 1**), with disagreements resolved by discussion or through consulation with a third author.

Box 1 **Inclusion and exclusion criteria**

Inclusion criteria

- Low-, middle-, or high-income country
- Infants and children 0 months to 10 years (119 months) of age suffering from acute watery or persistent diarrhoea
- Types of interventions: studies that compared the effect of oral zinc vs no zinc in managing acute watery or persistent diarrhoea
- Studies in which zinc high dose was compared to low dose of zinc
- Comparison group: studies in which the comparison group was no zinc, studies in which different doses of zinc were compared, studies in which the difference in the two groups was zinc and if other standard interventions, such as standard WHO ORS, intravenous fluid, antibiotics, supportive care were provided to both groups
- Relevant study designs: RCTs (individual or cluster)

Exclusion criteria

- Studies focussing solely on prevention of diarrhoea in children, rather than treatment; studies focussing on participants with chronic diarrhoea (duration of four weeks or greater); studies where placebo was a prebiotic, bulking agent, or any other agent that affects any of the outcomes of interest and is not being given to the intervention group
- Irrelevant study designs: case reports, case series, cohort studies, opinions, editorials, commentaries, letters, conference abstracts, reviews or systematic reviews, and studies with external comparison groups (i.e. historical cohort etc.)

### Data extraction and management

Two authors (ZH, AAA) independently extracted data from the included studies in duplicate into a standardised data extraction form in Microsoft Excel 2410 (Microsoft Corp., Redmond, Washington, USA) that had previously been pilot tested independently by two reviewers using three included studies. This included data on the study (journal, publication year, study design, location, study period), participants (age, number, inclusion criteria, type of diarrhoea), intervention (duration of zinc treatment, provider, frequency, dose), comparison or control group, outcomes (definition, time of assessment, units, dichotomous or continuous data), study limitations, and funding sources. We contacted corresponding authors for missing information, but did not receive any data in this way.

Three authors (ZH, AAA, MBAZ) then independently assessed the methodological quality of the included RCTs using Risk of Bias 2 (RoB2) tool, with disagreements resolved by discussion or consultation with a third author (SKN). The overall risk of bias could be reated as ‘low’, ‘high’, and ‘some concerns’ [18]. The tool assesses trials on the following domains: randomisation process, deviations from the intended interventions, missing outcome data, measurement of the outcome, and selection of the reported result.

### Statistical analysis

We conducted the meta-analysis in RevMan, version 5.4.1 (Cochrane, London, UK). We used risk ratios (RRs) to synthesise dichotomous outcomes and mean differences (MDs) or standardised mean difference (SMDs) along with a 95% confidence intervals (CIs) to present continuous outcomes. We converted any data reported as medians, interquartile ranges (IQRs), or CIs to means and standard deviations (SDs) using Hozo’s method [19]. We adjusted for studies that reported zero means or SDs by using very small nonzero values (i.e. 0.0001) in the meta-analysis, and used SDs from similar studies in cases where a study did not report SD, standard error, range, or CI for the data set. For trials with multiple arms, we entered mean and SD values separately for each arm in the analysis; if there was only one control group, we halved the number of participants for both continuous and dichotomous outcomes [20].

We assessed statistical heterogeneity using τ^2^, *I*^2^, and significance of the χ^2^ test, as well as by visually inspecting forest plots. We performed a random or fixed-effect analysis based on statistical heterogeneity for all comparisons. We also planned to adjust for clustering using an intraclass correlation coefficient, but the included studies adjusted all the outcomes for clustering.

### Sensitivity analysis

We performed sensitivity analyses on all outcomes in cases where studies were at high risk of bias or had some concerns in two or more domains. We otherwise did not perform sensitivity analyses for studies at low risk of bias or with some concerns in only one domain.

### Subgroup analysis

We conducted subgroup analyses on several reported outcomes selected based on previous review of literature, as well as discussions among the authors and with the WHO Guideline Development Group:

- Age categories
- Reporting time
- Socioeconomic status of the country where the trial was conducted
- Dose of zinc
- Form and formulation of zinc
- Duration of treatment
- Study setting – inpatient or outpatient
- Definition of diarrhoea

### Quality assessment

We conducted the Grading of Recommendations Assessment, Development and Evaluation (GRADE) assessment for all the included outcomes using GRADEPro and generated evidence profiles [21]. The outcomes were assessed based on the risk of bias, inconsistency, indirectness, imprecision, and publication bias. We then rated the certainty of the evidence for each outcome as ‘very low’, ‘low’, ‘moderate’ or ‘high’ [22].

## RESULTS

**Figure 1:**
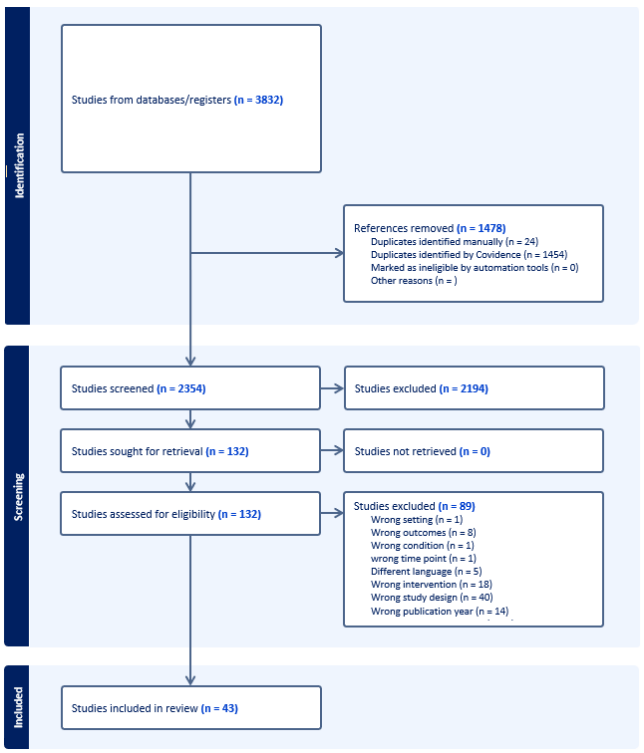
PRISMA flow diagram.

Our search retrieved 3832 records, with 2354 remaining for title/abstract screening following deduplication. We the reviewed 132 full texts, including 23 studies from cross-referencing, and included 43 studies in our review for acute watery and persistent diarrhoea (**Figure 1**).

### Study characteristics

We included 38 primary trials from 43 published studies, encompassing 33 693 children in total (**Table 1**). Most trials (n = 35) included children with acute diarrhoea; only three [62,64,66] included children with persistent diarrhoea (Tables S6 in the **Online Supplementary Document**). One trial reported results of two intervention groups, both receiving zinc supplements but in different dosages [31]; one included different arms for two formulations (ORS or syrup) [26]; and one trial presented results divided into two subgroups based on zinc levels (low or normal) at enrollment [50]. Three trials reported data for study sites in multiple countries [25,37,65].

**Table 1:** Study characteristics

	Country	Setting	Age	Formulation	Form	Duration in days	Dose
**Zinc vs no zinc in acute diarrhoea**							
Ahmadipour et al., 2019 [23]	Iran	Inpatient	6 mo to 2 y	Elemental zinc as zinc gluconate	Syrup	3–7	20 mg
Al Sonboli et al., 2003 [24]	Brazil	Inpatient	3 to 60 mo	Elemental zinc	Dispersible tablet	Max of 5 d or less if resolution of diarrhoea	3–6 mo: 22.5 mg; 7–60 mo: 45mg
Awasthi et al., 2006 [25]	Brazil, Ethiopia, India, Egypt, Phillipines	Outpatient	2 to 59 mo		Dispersible tablet	14	Ethiopia and Lucknow, India gave 2 zinc tablets whereas the other sites gave 1 tablet once per day
Bahl et al., 2002 [26]	Delhi, India	Outpatient	6 to 35 mo	Elemental zinc as gluconate	Syrup and ORS both	14	12–35 mo: 30 mg; 6–11 mo: 15 mg
Baqui et al., 2002 [27]	Bangladesh	Outpatient	3 to 47 mo	Elemental zinc as acetate	Syrup	14	20 mg
Bhandari et al., 2008 [28]	India	Outpatient	1 to 59 mo		Dispersible tablet	14	10 mo for <6 mo, 20 mg >6 mo
Bhatnagar et al., 2004 [29]	India	Inpatient	3 to 36 mo	Zinc sulfate had 1 mg per milliliter of elemental zinc	Syrup	14	1 mg/ml of elemental zinc. Participants <12 and >12 mo were given 15 mL and 30 mL of zinc in 3 divided doses.
Boran et al., 2005 [30]	Turkey	Outpatient	6 to 60 mo	Zinc sulfate	Syrup	14	15 mg for 6–12 mo and 30 mg for 12–60 mo old
Brooks et al., 2005 [31]	Bangladesh	Inpatient	1 to 6 mo	Zinc acetate	Syrup		20 mg and 5 mg
Crisinel et al., 2015 [32]	Switzerland	Both	2 mo to 5 y	Zinc sulphate	Dispersible tablet	10	10 mg once a day (QD) in children <6 mo and 20 mg QD for children ≥6 mo.
Dalgic et al., 2011 [33]	Turkey	Inpatient	1 to 28 mo	Zinc acetate	Suspension		Less than 6 mo 10mg/d more than 6 mo 20 mg/d
Dutta et al., 2000 [34]	India	Inpatient	3 to 24 mo	Zinc sulphate	Syrup	Until recovery or 5 d max	40 mg
Dutta et al., 2011 [35]	India	Inpatient	6 to 24 mo	Elemental zinc		14	20 mg
Elnemr et al., 2007 [36]	Yemen	Both	3 mo to 2 y	Zinc acetate	Syrup	14	20 mg
Fischer Walker et al., 2006 [37]	Pakistan, India, Ethiopia	Outpatient	1 to 5 mo		Dispersible tablet	14	10 mg
Fischer Walker et al., 2007 [38]		Outpatient	3 to 24 mo	Elemental zinc	Syrup		20 mg
Fischer Walker et al., 2008 [39]	Bangladesh	Outpatient	3 to 47 mo	Zinc acetate		14	20 mg
Gregorio et al., 2007 [40]	Philippines	Outpatient	2 to 59 mo	Zinc sulfate	Dispersible tablet	14	20 mg
Karamyyar et al., 2013 [41]	Iran	Inpatient	9 mo to 5 y	Zinc sulfate	Syrup		1 mg/kg/d
Kakar et al., 2022 [42]	Pakistan		3 mo to 5 y				
Larson et al., 2005 [43]	Bangladesh	Both	3 to 59 mo	Zinc sulphate	Dispersible tablet	10–14	20 mg
Mazumder et al., 2010 [44]	India	Outpatient	1 to 5 mo	20 mg zinc	Dispersible tablet		10 mg
Patel et al., 2013 [45]	India	Both	6 to 59 mo	Zinc sulphate	Syrup	14	Zinc 2 mg/kg/d
Patel et al., 2013 (2) [46]	India	Both	6 to 59 mo	Zinc sulphate	Syrup	14	Zinc 2 mg/kg/d
Patel et al., 2009 [47]	India	Both	6 to 59 mo	Zinc sulphate	Syrup	14	Zinc 2 mg/kg/day
Patel et al., 2015 [48]	India	Both	<12 y	Zinc sulphate		14	20 mg/d for patients aged >6 mo and 10 mg/d for infants aged <6 mo.
Patro et al., 2010 [49]	Poland	Both	3 to 48 mo	Zinc sulfate	Syrup	10	10 mg for <6 mo and 20mg for >6 mo.
Polat et al., 2003 [50]	Turkey	Outpatient	2 to 29 mo	Zinc sulfate	Syrup	14	20
Rerksuppaphol et al,, 2020 [51]	Thailand	Inpatient	≥ 6 mo	Zinc bisglycinate (15 mg elemental zinc)	Powder form in a single dose sachet and dissolved in water	Until discharge from hospital	15
Roy et al., 2008 [52]	Bangladesh	Inpatient	3 to 14 y	Elemental zinc as acetate	Syrup	Until resolution of diarrhoea or for up to seven days	30
Shah et al., 2021 [53]	Pakistan	Inpatient	6 mo to 5 y	Oral zinc		14	20
Shahzad et al., 2022 [54]	Pakistan		6 mo to 5 y	Zinc sulfate	Syrup	10–14	20
Shimelis et al., 2008 [55]	Ethiopia	Outpatient	2 to 59 mo		Dispersible tablet	14	20
Strand et al., 2002 [56]	Nepal	Outpatient	6 to 35 mo		Syrup	Daily supplementation during diarrhoea until 7 d after recovery	15 and 30
Trivedi et al., 2009 [57]	India	Inpatient	6 to 59 mo	Zinc sulfate	Syrup	Continued till they were get cured and discharged	10 mg for less than 1 y, 20mg for more than 1 y
Valery et al., 2005 [58]	Australia	Inpatient	less than 11 y	Elemental zinc (zinc sulphate)		5	less than 1 y: 20 mg, 1–10 y: 40 mg
Wadhwa et al., 2011 [59]	India	Inpatient	1 to 35 mo	Elemental zinc as zinc gluconate	ORS		10 mg elemental zinc: Infants <6 mo; 20 mg/d: children >6 mo
Yalcin et al., 2022 [60]	Turkey	Outpatient	6 mo to 6 y		Suspension	10	20 mg
Yazar et al., 2016 [61]	Turkey	Outpatient	6 to 120 mo		Suspension	5	15 mg
**Zinc vs no zinc in persistent diarrhoea**							
Wang et al., 2016 [62]	China	Inpatient	Not found	Not found	Not found	14	10 mg/d: Children <6 mo; 20 mg/d: children ≥6 mo
Khatun et al., 2001 [63]	Bangladesh	Not mentioned	6 mo to 2 y	Zinc acetate	syrup	7	20 mg
Roy et al., 2007 [64]	Bangladesh		3 to 24 mo	Elemental zinc	Syrup	14	20 mg
**High dose vs low dose zinc in acute diarrhoea**							
Dhingra et al., 2020 [65]	India and Tanzania	Outpatient	6 to 59 mo	Zinc sulfate regimens	Dispersible tablet	14	5 mg, 10 mg or 20 mg

mo – month, ORS – oral rehydration solution, y – year

Most of the trials (n = 21) used 20 mg zinc as the intervention for acute diarrhoea; three trials [24,34,58] gave 40 mg zinc; three trials [26,52,56] used 30 mg zinc; six trials gave lower (usually half) dosages of zinc to children below six months of age and these doses (range: 10 to 22.5 mg); and two trials [41,45] gave 1 and 2 mg per kilogram of child’s bodyweight per day to the participants, respectively. Fourteen trials used zinc sulfate [29,30,32,34,40,41,43,48–50,54,57,58,65], six used zinc acetate [27,31,33,36,39,52], three used zinc gluconate [26,59], three [24,35,64] mentioned elemental zinc only, and the remaining trials did not specify the exact zinc salt used as the intervention [28,42,44,53,55,56,60,61].

Seventeen trials administered zinc supplements for a period of 14 days [25-30,34,36–40,43,46–48,50,53-55]; two trials [23,52] gave zinc for three to seven days; two trials [24,34] gave it for a maximum of five days depending on symptom resolution; two trials [58,61] gave for five days; and three trials [32,49,60] gave for a total of 10 days. Participants in one trial [57] received zinc until they were cured and discharged from the hospital.

### Comparison 1: Zinc vs placebo for children with acute diarrhoea

#### Risk of bias

**Figure 2:**
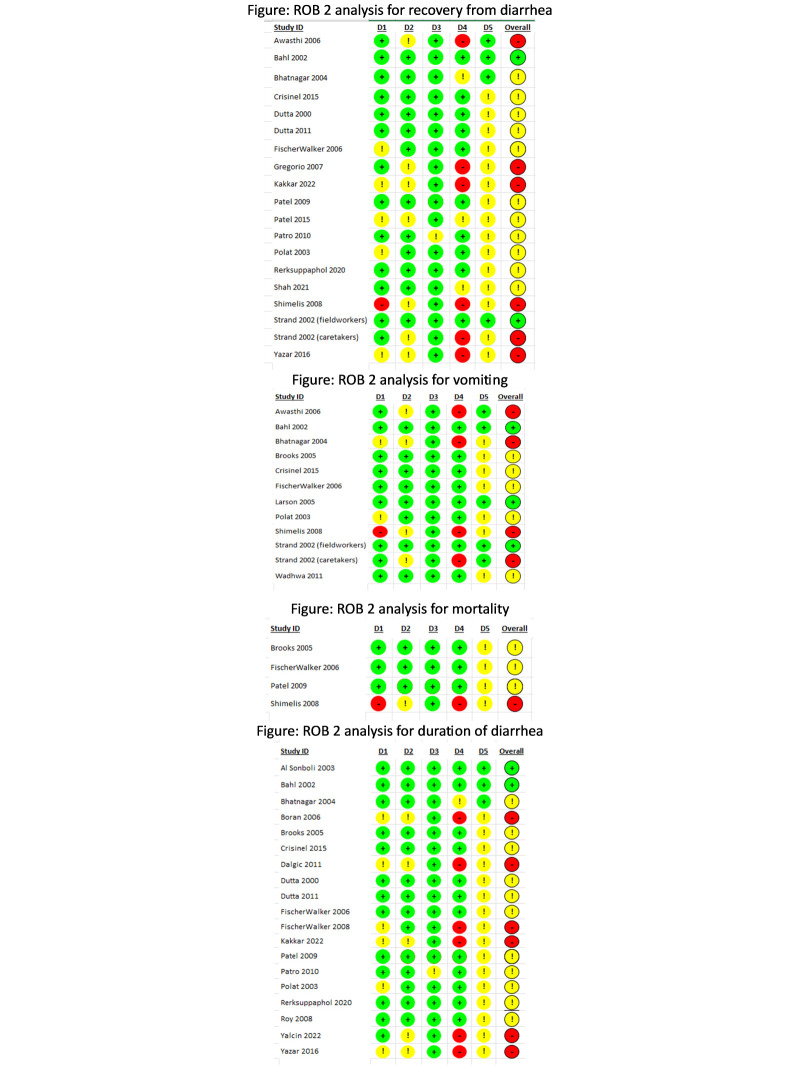
RoB 2 assessment for acute diarrhoea.

For the outcome of recovery from acute diarrhoea, the intervention allocation methods used in five trials showed some concerns [37,42,48,50,61] and high risk of bias in one trial [55]. For deviations from the intended interventions, seven trials showed some concerns [25,40,42,48,55,56,61]. There were some concerns about missing outcome data for one trial [49]. The measurement methods for three trials had some concerns [29,48,53], and eight trials showed high risk of bias [25,37,40,42,55,56,61]. Fifteen trials showed some concerns due to selective reporting of outcomes, about whether or not they used multiple measurement or analysis methods, or if these methods adhered strictly to the study protocol or not [32,34,35,37,40,42,47,49–51,53,55,56,61,67] (**Figure 2**).

For the outcome of vomiting after taking zinc, the intervention allocation methods used in two of the trials either had some concerns [29,50] and put one study at high risk of bias [55]. For four of the trials, there were some concerns about deviations from the intervention [25,29,55,56]. Four trials were at high risk of bias for the methods of measurement used [25,29,55,56]. In seven trials, there were some concerns about whether or not they used multiple measurements or analysis methods, or if these methods adhered strictly to the study protocol [29,31,32,37,50,55,59].

For the outcome of mortality from acute diarrhoea, the allocation sequence used in one trial was at high risk of bias [55]. In one trial, there were some concerns about whether or not there was deviation from intended intervention [55]. One trial was at high risk of bias for the methods of measurement used [55]. With regard to the selection of the reported result, there were some concerns for all four trials about whether or not they used multiple measurements or analysis methods, or if these methods adhered strictly to the study protocol [31,37,47,55].

For the outcome of duration of diarrhoea, two trials showed an overall low risk of bias [24,26]. The allocation sequence used in six trials showed some concerns[30,33,39,42,50,61]. For five of the trials, there were some concerns about deviations from the intervention [30,33,42,60,61]. One trial showed some concerns for missing outcome data [49]. Six trials were at high risk of bias for the methods of measurement used [30,33,39,42,60,61], while one showed some concerns in this domain [29]. In seventeen trials, there were some concerns about whether or not they used multiple measurement or analysis methods, or if these methods adhered strictly to the study protocol or not [30–35,37,39,42,47,49–52,60,61].

### Effect estimates

#### Recovery

**Figure 3:**
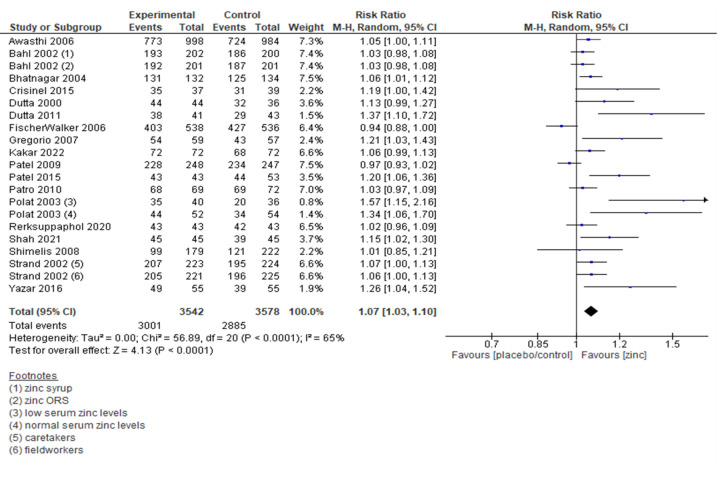
Forest plot for recovery from diarrhoea.

There were some variations in the definition of recovery from diarrhoea among the studies that clearly reported outcome definitions (Table S6 and S7 in the **Online Supplementary Document**). Overall, the results suggested that the treatment with zinc resulted in more children recovering from diarrhoea at the last follow-up (RR = 1.07; 95% CI = 1.03, 1.1; 7120 children, 18 trials, moderate certainty of evidence) (**Figure 3**). The sensitivity analysis also showed similar results.

In the subgroup analysis by follow-up day, there was a significant increase in recovery from diarrhoea at day five; however, on day seven, the number of children who had recovered was comparable in both zinc and control groups. Studies that followed the WHO definition of diarrhoea and those that used other definitions both showed a significantly higher proportion of children who had recovered from diarrhoea in the zinc group. Subgroup analysis by the dose of zinc administered showed a comparable proportion of children recovered from diarrhoea at last follow-up in the 10, 15, and 40 mg zinc groups. There was a significantly higher proportion of children who had recovered by the last follow-up in the 20 mg zinc group and dose based on age or weight categories compared to the control group. A significantly higher proportion of children had recovered from diarrhoea in groups receiving zinc for five days and 14 days; however, the effect of 10 days of zinc supplementation was comparable among the two groups. Administration of zinc sulfate and zinc gluconate had a significantly favorable effect on the proportion of children who recovered from diarrhoea, while the proportion of children who had recovered was comparable in the zinc bisglycinate (**Table 2**; Figure S1–10 in the **Online Supplementary Document**).

**Table 2:** Subgroup estimates for acute diarrhoea

	Recovery, RR (95% CI)	Number of studies	Vomiting, RR (95% CI)	Number of studies	Mortality, RR (95% CI)	Number of studies	Duration of diarrhoea, MD (95% CI)	Number of studies
**Definition of diarrhoea**								
WHO definition	1.04 (1.01, 1.08)	11	1.37 (1.15, 1.63)	7	0.71 (0.10, 4.88)	3	−11.26 (−17.51, −5.00)	14
Other definitions	1.11 (1.03, 1.19)	5	1.46 (0.83, 2.55)	3	Not estimable	1	−16.69 (−27.78, −5.60)	4
Unspecified definition	1.23 (1.09, 1.40)	2	2.17 (1.65, 2.84)	1			−22.21 (−33.32, −11.10)	2
**Dose of zinc**								
5 mg			2.73 (0.78, 9.61)	1			0.00 (−4.43, 4.43)	1
10 mg	0.94 (0.88, 1.00)	1	1.42 (0.92, 2.18)	1	1.00 (0.06,15.8)	1	12.00 (1.63, 22.37)	1
15 mg	1.13 (0.80, 1.58)	2					−24.85 (−33.31, −16.39)	2
20 mg	1.22 (1.10, 1.36)	5	2.20 (1.75, 2.75)	4	Not estimable	1	−17.62 (−27.35, −7.88)	6
30 mg							−8.70 (−9.58, −7.82)	1
40 mg	1.13 (0.99, 1.27)	1					−33.00 (−39.32, −26.68)	1
Dose based on weight or age	1.04 (1.01, 1.08)	7	1.30 (1.04, 1.61)	5	0.50 (0.03, 7.86)	2	−13.33 (−24.81, −1.84)	7
Unspecified dose	1.05 (1.01, 1.10)	2	1.27 (0.93, 1.72)	1			−4.57 (−17.73, 8.59)	2
**Duration of supplementation**								
≤5-d supplementation	1.17 (1.04, 1.31)	2					−31.75 (−36.96, −26.54)	3
≤10-d Zinc supplementation	1.09 (0.91, 1.31)	2	1.24 (0.90, 1.69)	1			−4.55 (−10.49, 1.39)	4
14-d zinc supplementation	1.07 (1.02, 1.12)	11	1.32 (1.03, 1.67)	6	0.66 (0.11, 3.96)	3	−12.86 (−20.98, −4.73)	9
Unspecified duration of zinc supplementation	1.05 (1.02, 1.09)	3	1.72 (1.24, 2.40)	4	Not estimable	1	−13.96 (−24.81, −3.10)	4
**Zinc formulation**								
Zinc sulfate	1.12 (1.04, 1.20)	8	1.48 (0.96, 2.27)	4	0.50 (0.03, 7.86)	1	−15.70 (−27.12, −4.27)	7
Zinc gluconate	1.04 (1.01, 1.07)	2	1.08 (0.79, 1.48)	2			−4.74 (−11.74, 2.26)	1
Zinc acetate			2.04 (0.86, 4.85)	1	Not estimable	1	−14.44 (−22.71, −6.17)	4
Zinc bisglycinate	1.02 (0.96, 1.09)	1					−21.30 (−33.75, −8.85)	1
Unspecified zinc formulation	1.08 (1.00, 1.16)	7	1.70 (1.36, 2.12)	4	1.00 (0.06, 15.89)	2	−11.26 (−20.90, −1.63)	7
**World Bank income-level classification**								
LMIC	1.07 (1.03, 1.11)	13	1.52 (1.21, 1.90)	9	0.71 (0.10, 4.88)	4	−11.26 (−16.27, −6.30)	12
HIC	1.09 (0.98, 1.21)	4	1.24 (0.90, 1.69)	1			−17.97 (−30.21, −5.73)	8
Mixed	1.05 (1.00, 1.11)	1	1.27 (0.93, 1.72)	1				
**Zinc formulation**								
Syrup	1.07 (1.02, 1.12)	8	1.59 (1.20, 2.11)	5			−13.87 (−19.82, −7.92)	11
ORS	1.03 (0.98, 1.08)	1	0.95 (0.64 to 1.40)	2			−2.40 (−12.17, 7.37)	1
Dispersible tablet	1.06 (0.97, 1.15)	5	1.61 (1.22, 2.13)	5			−9.63 (−30.03, 10.77)	4
Powder sachet	1.02 (0.96, 1.09)	1					−21.30 (−33.75, −8.85)	1
Unspecified	1.16 (1.04, 1.31)	4					−16.13 (−28.36, −3.90)	4
**Age**								
>6 mo	1.05 (1.01, 1.10)	7	1.42 (1.06, 1.90)	3	0.50 (0.03, 7.86)	1	−11.86 (−19.75, −3.97)	7
<6 mo	0.94 (0.88, 1.00)	1	1.52 (1.04, 2.23)	2	1.00 (0.06, 15.8)	2	2.05 (−3.01, 7.12)	2
>6 and <6 mo	1.09 (1.05, 1.14)	10	1.49 (1.10, 2.01)	6	Not estimable	1	−19.64 (−27.18, −12.11)	11
**Setting**								
Inpatient	1.10 (1.02, 1.19)	5	1.12 (0.97, 1.28)	3	Not estimable	1	−17.77 (−25.27, −10.27)	8
Outpatient	1.07 (1.03, 1.12)	9	1.53 (1.21, 1.93)		1.00 (0.06, 15.8)	2	−14.26 (−22.71, −5.81)	8
Both	1.02 (0.95, 1.10)	3	1.65 (0.91, 2.97)		0.50 (0.03, 7.86)	1	2.90 (−2.57, 8.37)	3
Unspecified	1.06 (0.99, 1.13)	1					−11.23 (−17.03, −5.43)	1
**Time point**								
At day 5	1.08 (1.04, 1.12)	13						
At day 7	1.03 (1.00, 1.07)	8						

CI – confidence interval, LMIC – low- and middle-income country, HIC – high income country, MD – mean difference, ORS – oral rehydration solution, RR – risk ratio, WHO – World Health Organization

#### Vomiting

**Figure 4:**
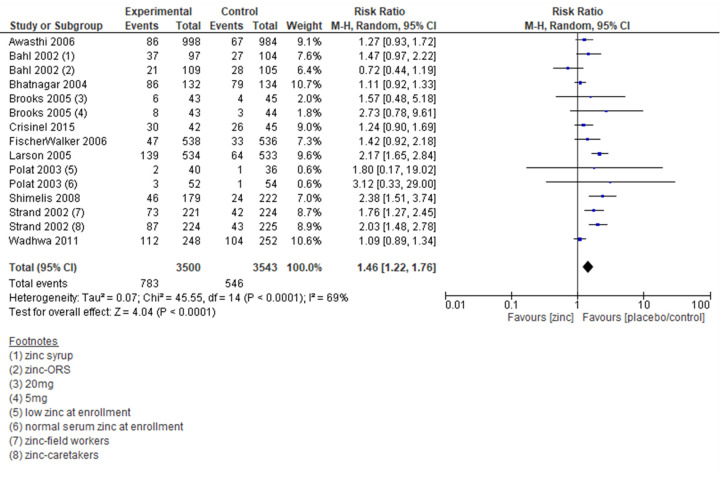
Forest plot for frequency of vomiting.

The results suggest that patients in the zinc supplementation group had a higher risk of vomiting compared to the control/placebo group (RR = 1.46; 95% CI = 1.22, 1.76; 11 trials, 7043 participants, moderate certainty of evidence) (**Figure 4**). The sensitivity analysis showed a similar effect.

In the subgroup analysis by definition of diarrhoea, there was a significant increase for trials that used the WHO definition of diarrhoea, while the risk was comparable in trials that used other definitions of diarrhoea. In the subgroup analysis by the dose of zinc, the risk was comparable in children receiving 5 mg and 10 mg zinc, while the risk of vomiting was higher in children 20 mg zinc or doses based on age or weight. Subgroup analysis based on the duration of zinc supplementation showed a comparable effect on vomiting in those getting zinc for 10 days, while the risk of vomiting was statistically significant in the 14 days group. Subgroup analysis based on the formulation of zinc showed that the risk of vomiting was comparable in zinc gluconate, zinc sulfate, and zinc acetate groups.(**Table 2**; Figure S11–19 in the **Online Supplementary Document**).

#### Mortality

**Figure 5:**
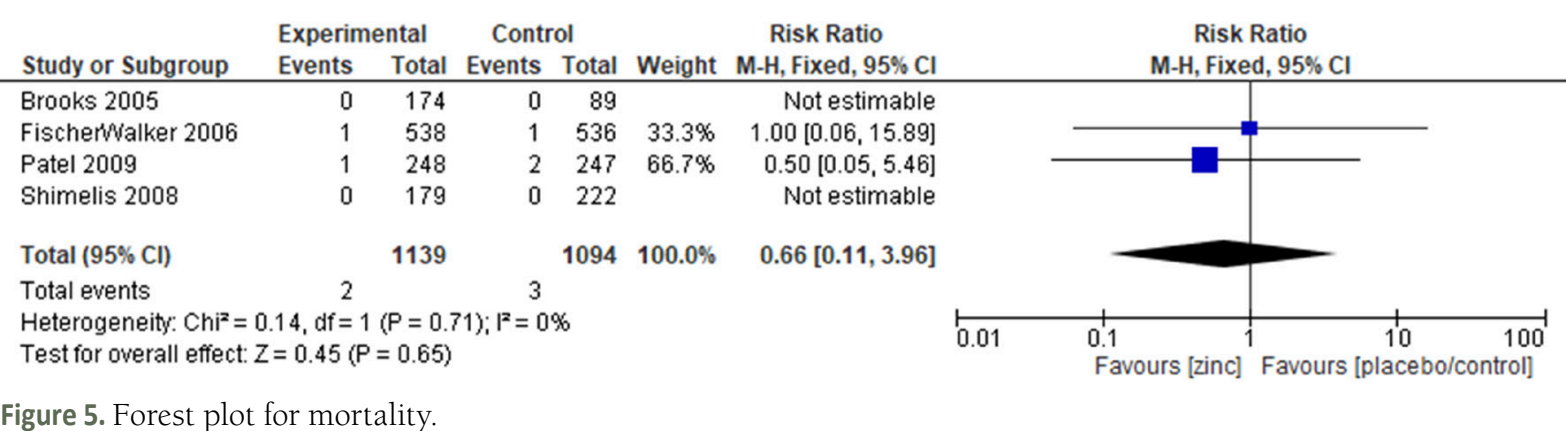
Forest plot for mortality.

Four trials reported on the outcome of mortality, with the meta-analysis showing comparable results in the zinc and the control group (RR = 0.71; 95% CI = 0.10, 4.88; four trials, 2109 participants, moderate certainty of evidence) (**Figure 5**). The sensitivity analysis showed a similar effect. Subgroup analyses by definition of diarrhoea, dose of zinc, duration of zinc supplementation, and zinc formulation showed comparable mortality risk in zinc intervention and control groups (**Table 2**; Figure S20–26 in the **Online Supplementary Document**).

#### Duration of diarrhoea

**Figure 6:**
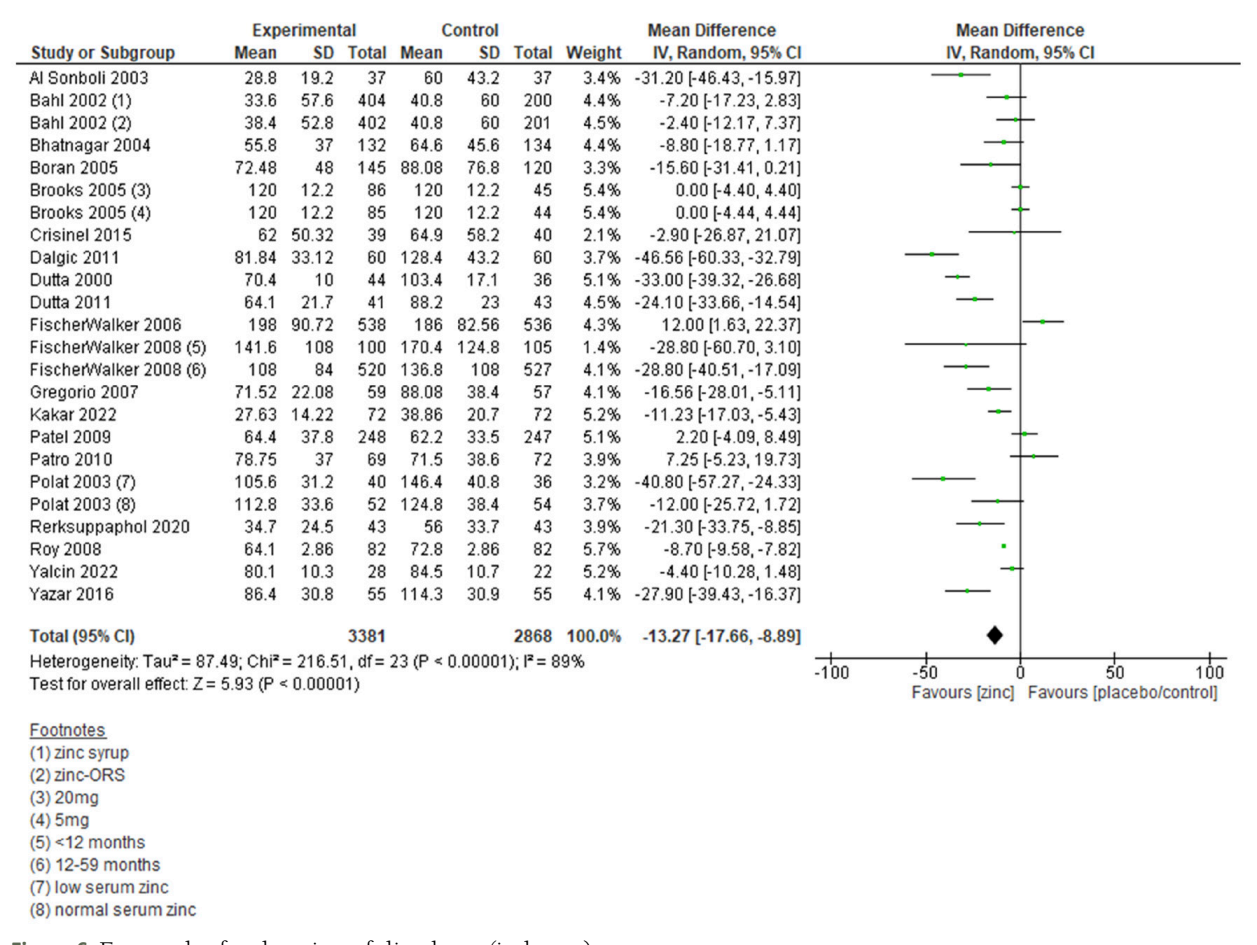
Forest plot for duration of diarrhoea (in hours).

Twenty-four trials reported on the duration of diarrhoea, and the associated meta-analysis suggested that the duration of diarrhoea decreased significantly in the zinc group as compared to no zinc/placebo group (MD = −13.27 hours; 95% CI = −17.66, −8.89; 24 trials, 6249 participants, moderate certainty of evidence) (**Figure 6**). The sensitivity analysis showed similar results.

Sub-group analysis by definition of diarrhoea showed a significant decrease in durationof diarrhoea in all subgroups. Sub-group analysis by dose of zinc showed a significant decrease in duration for 15 mg, 20 mg, 30 mg, 40 mg, and dose based on age or weight bands. Sub-group analysis by the duration of zinc supplementation showed a significant decrease in the duration of diarrhoea for five days, 14 days, and results were comparable for the 10-day zinc supplementation group. Sub-group analysis by the zinc formulation showed a significant decrease in the duration of diarrhoea in all groups except for zinc gluconate formulation, where the results were comparable between the two groups (**Table 2**; Figure S27–35 in the **Online Supplementary Document**).

### Comparison 2: Zinc vs no zinc for persistent diarrhoea

#### Risk of bias

**Figure 7:**
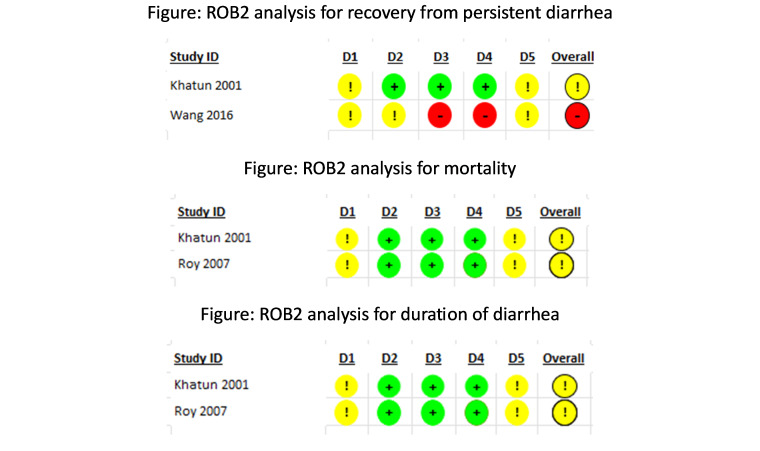
RoB 2 assessment for persistent diarrhoea.

For the outcome of recovery from persistent diarrhoea, both trials had some concerns about whether they used adequate randomisation methods to generate the allocation sequence (**Figure 7**). There were some concerns about one trial [62] for whether any deviation from intended intervention occurred, a high risk of bias for missing outcome data, and a high risk of bias for the methods of outcome measurement or ascertainment [62]. For the selection of the reported result, there were some concerns about both trials [62,63], whether they used multiple measurement or analysis methods, or whether these methods adhered strictly to the study protocol.

For mortality and duration of diarrhoea, there were some concerns about whether they used adequate randomisation methods to generate the allocation sequence. For the selection of the reported result, there were some concerns for both trials [63,64] about whether or not they used multiple measurement or analysis methods, or if the methods adhered strictly to the study protocol or not.

#### Effect estimates

Two trials reported on the outcome of recovery from diarrhoea either on day five or day seven. The meta-analysis suggests a significantly higher proportion of children had recovered from diarrhoea in the zinc group compared to the control/placebo (RR = 1.75; 95% CI = 1.34, 2.30; two trials, 112 participants, low certainty of evidence) (Figure S36 in the **Online Supplementary Document**). Mortality during the study period was comparable between the zinc and placebo/control groups (RR = 0.84; 95% CI = 0.29, 2.37; two trials, 202 participants, moderate certainty of evidence) (Figure S37 in the **Online Supplementary Document**).

Two trials reported on the duration of diarrhoea. The meta-analysis showed that the duration of diarrhoea decreased significantly in the zinc group when compared to the placebo/control group (RR = −26.29; 95% CI = −47.35, −5.23; two trials, 242 participants, very low certainty of evidence) (Figure S38 in the **Online Supplementary Document**).

### Comparison 3: High dose vs low dose zinc supplementation

#### Risk of bias

There was only one study included for high dose vs low dose of zinc comparison. It showed an overall low risk of bias for the number of participants who had diarrhoea more than five days after starting zinc and the number of participants with vomiting in 5 mg and 10 mg zinc groups compared to 20 mg zinc group (Figure S39 in the **Online Supplementary Document**).

#### Effect estimates

The study reported the number of children who had diarrhoea for greater than five days after starting zinc supplementation, and the results suggested that continued diarrhoea after five days was comparable between the two groups (RR = 1.15; 95% CI = 0.91, 1.44) (one trial, 4439 participants, low certainty of evidence) (Figure S40 in the **Online Supplementary Document**). While the risk of vomiting significantly decreased in the 5 mg/10 mg zinc groups compared with the 20 mg zinc group (RR = 0.79; 95% CI = 0.70, 0.88) (one trial, 4474 participants, moderate certainty of evidence) (Figure S41 in the **Online Supplementary Document**). The full GRADE assessment is provided in **Table 3** and Table S9 in the **Online Supplementary Document**.

**Table 3:** GRADE evidence profile for acute diarrhoea

Certainty assessment							Number of patients		Effect			
**Outcome**	**Study design**	**Risk of bias**	**Inconsistency**	**Indirectness**	**Imprecision**	**Other considerations**	**Zinc, n/N (%)**	**No zinc, n/N (%)**	**Relative, RR (95% CI)**	**Absolute (95% CI)**	**Certainty**	**Importance**
Recovery (18 studies)	Randomised trials	Not serious*	Serious†	Not serious	Not serious	None‡	3001/3542 (84.7)	2885/3578 (80.6)	1.07 (1.03, 1.10)	56 more per 1000 (from 24 more to 81 more)	Moderate	CRITICAL
Vomiting (11 studies)	Randomised trials	Not serious§	Serious‖	Not serious	Not serious	None‡	783/3500 (22.4)	546/3543 (15.4)	1.46 (1.22, 1.76)	71 more per 1000 (from 34 more to 117 more)	Moderate	Critical
Mortality (four studies)	Randomised trials	Not serious¶	Not serious	Not serious	Serious**	None	2/1139 (0.2)	2/970 (0.2)	0.71 (0.10, 4.88)	1 fewer per 1000 (from 2 fewer to 8 more)	Moderate	Critical
Duration of diarrhoea in hours (20 studies)	Randomised trials	Not serious††	Serious‡‡	Not serious	Not serious	None	3381	2868	-	MD = 13.27 lower (17.66 lower to 8.89 lower)	Moderate	Critical

CI – confidence interval, MD – mean difference, RR – risk ratio

*High risk of bias in some domains for four studies; however, all reported estimates including the null/no meaningful difference.

†*P*-value <0.0001 and *I*^2^ 66%.

‡Asymmetrical funnel plot; however, larger studies and studies with lower risk of bias concerns still report benefit.

§Despite high risk of bias assessment in three studies and some concerns in five others; sensitivity analysis excluding studies at high risk of bias are robust.

‖*I*^2^ value is 69%. Subgroup analyses of dose (increased dose leading to increased vomiting) and definition of diarrhoea contribute statistical heterogeneity.

¶Two high risk studies and two with some concerns; however, only one study contributes to the analysis and is consistent with the study without risk of bias concerns.

**CI crossing null value.

††Six studies at high risk of bias, all others with some concerns except two at low risk of bias; however, sensitivity analysis of risk of bias removing studies with serious concerns does not change the effect estimate substantially.

‡‡*P*-value <0.00001, *I*^2^ = 89%.

## DISCUSSION

The results for the treatment of children with acute diarrhoea suggested that recovery was significantly higher in the zinc group, at a dose of 20 mg, when zinc is administered for five or 14 days in the form of zinc sulfate or zinc gluconate. Zinc supplementation also led to a reduction in the duration of diarrhoea and the duration decreased significantly when zinc was administered for five or 14 days, while mortality was comparable between the zinc group and placebo/control groups. However, the risk of vomiting was also higher in the zinc group at a dose of 20 mg or when zinc is administered for 14 days. For the treatment of children with persistent diarrhoea, zinc supplementation improved recovery and reduced the duration of diarrhoea, whereas mortality was comparable. For the comparison of high dose vs low dose of zinc, the recovery was comparable, but the risk of vomiting decreased significantly for the 5 mg/10 mg dose.

Our findings are in line with existing guidelines and reviews [12,13,68,69], while our subgroup analyses on the optimal dose, frequency, and formulation add new knowledge to the existing evidence base. In 2004, the WHO and UNICEF endorsed the use of zinc as a treatment for childhood diarrhoea, indicating that the oral administration of zinc can reduce both the duration and severity of diarrhoea [14]. Since then, a substantial amount of work went into investigating the effects of oral zinc administration on diarrhoeal outcomes, including mortality and side effects, but especially vomiting [25,26,29,31,32,37,43,50,55,56,59], as it acts as a deterrent for the use of zinc supplementation. Our review also suggested that the risk of vomiting was 46% higher in patients receiving zinc. However, when comparing standard dose zinc vs low dose zinc, we observed that the risk of vomiting decreased significantly by 24% in the low dose group, i.e. 10 mg/5 mg group. These results suggest that low doses of zinc should be administered to reduce vomiting, as they achieve a similar efficacy as standard dose zinc. However, it remains crucial to persist in promoting suitable fluid and dietary therapy as the fundamental approach to decrease the morbidity and mortality associated with diarrhoea [70].

According to the RoB 2 assssment, five trials showed an overall low risk of bias and ‘some concerns’ were found in 16 trials mainly because of their selection of reported results,as most of the trials were not registered hence protocols were not available. Fifteen trials had high risk of bias for their randomisation process and measurement of outcomes. Four trials showed some concerns for one outcome and high risk of bias for another outcome. This included the study by Strand et al. [56], which showed overall low risk of bias for fieldworkers and high risk of bias for caretakers group due to high risk in domain measurement of the outcome, and the study by Bhatnagar et al. [29], which showed some concerns for measurement of the outcome for recovery and a high risk of bias for the outcome of vomiting. We noted some concerns in the selection of the reported result for duration of diarrhoea whereas a high risk of bias was observed in measurement of the outcome for recovery in the study by Fischer Walker et al. [37] and for the selection of the reported resultfor duration of diarrhoea in the study by Patel et al. [47].

The GRADE assessment [21,22] for acute diarrhoea suggested moderate certainty evidence for recovery and risk of vomiting for the zinc group (**Table 3**). The evidence was downgraded due to high heterogeneity. The evidence for mortality was of moderate certainty and was downgraded due to CIs crossing null value, and small sample size. Moderate certainty evidence suggests that there is a substantial decrease in mean duration of diarrhoea in zinc group. Evidence was downgraded because of high heterogeneity. The high heterogeneity values could be due to varying sample sizes, different times of outcome assessment, variations in diarrhoea severity and clinical response to zinc treatment, and different doses, duration and formulation of zinc utilised in different trials.

The strengths of this review lie in its robust methodology and the wide search strategy, as well as the inclusion of RCTs only. Subgroup analyses based on several important predictors, such as time point, definition of diarrhoea, dose, formulation, duration, and other factors further strengthened the review by answering important clinical questions. However, we only found two studies that included children above the age of five years. We also did not account for differences in baseline nutritional status or access to health care facilities among different populations. Additionally, the presence of some concerns and high risk of bias in studies highlights a lack of quality evidence and a need for cautious interpretation of our findings.

## CONCLUSIONS

Current evidence reinforces the benefits of zinc supplementation in the management of acute and persistent diarrhoea and recommends the continuation of guidelines for zinc supplementation in childhood diarrhoea although the current recommended dose could be reduced. Further large-scale multi-country randomised trials with direct comparisons are required for definitive dosage and duration of zinc supplementation for all age groups.

## Additional material


Online Supplementary Document
