# Supplementary material for: Zinc supplementation for acute and persistent watery diarrhoea in children: A systematic review and meta-analysis
Source: J Glob Health. 2024 Dec 6;14:04212. doi: 10.7189/jogh.14.04212 (PMC11622351; doi:10.7189/jogh.14.04212)
Supplement: Online Supplementary Document [file jogh-14-04212-s001.pdf]

# Online Supplementary File

## Search strategy

Table S1: PubMed

| Diarrhea | Children up to 10 years | Zinc |
|---|---|---|
| ((Diarrhea[MeSH Terms]) AND (Diarrhea[Title/Abstract] OR diarrhoea[Title/Abstract])) OR (Diarrhea, Infantile[MeSH Terms]) OR (“Infantile Diarrhea*”[Title/Abstract]) OR (“Childhood Diarrhea*”[Title/Abstract]))) | ((((Child[MeSH Terms]) OR (Child, Preschool[MeSH Terms]) OR (Child*[Title/Abstract] OR preschool*[Title/Abstract] OR Schoolchild*[Title/Abstract] OR “school age”[Title/Abstract] OR Kid[Title/Abstract] OR Kids[Title/Abstract] OR toddler*[Title/Abstract]) OR (Infant[MeSH Terms]) OR (Infant*[Title/Abstract] OR Infancy[Title/Abstract] OR Baby[Title/Abstract] OR Babies[Title/Abstract] OR Newborn*[Title/Abstract] OR Neonat*[Title/Abstract] OR Preterm[Title/Abstract] OR Prematur*[Title/Abstract]) OR (Pediatrics[MeSH Terms]) OR (Pediatric*[Title/Abstract] OR Paediatric*[Title/Abstract]) OR (Schools, Nursery[MeSH Terms]) OR (Nursery school[Title/Abstract] OR Kindergar*[Title/Abstract])) | ((Zinc[MeSH Terms]) OR (Zinc[Title/Abstract] OR (Zn[Title/Abstract])))) |

Table S2: Cochrane Central Register of Controlled Trials

| Children up to 10 years | Diarrhea | Zinc |
|---|---|---|
| #1 MeSH descriptor: [Child] explode all trees<br><br>#2 MeSH descriptor: [Child, Preschool] explode all trees<br><br>#3 (Child* OR preschool* OR Schoolchild* OR “school age” OR Kid OR Kids OR toddler*):ti,ab,kw<br><br>#4 MeSH descriptor: [Infant] explode all trees<br><br>#5 (Infant* OR Infancy OR Baby OR Babies OR Newborn* OR Neonat* OR Preterm OR Prematur*):ti,ab,kw<br><br>#6 MeSH descriptor: [Pediatrics] explode all trees<br><br>#7 (Pediatric* OR Paediatric*):ti,ab,kw<br><br>#8 MeSH descriptor: [Schools, Nursery] explode all trees<br><br>#9 (Nursery school OR Kindergar*):ti,ab,kw<br><br>#10 #1 OR #2 OR #3 OR #4 OR #5 OR #6 OR #7 OR #8 OR #9 | #11 MeSH descriptor: [Diarrhea] explode all trees<br><br>#12 ((Diarrhea OR diarrhoea)):ti,ab,kw<br><br>#13 MeSH descriptor: [Diarrhea, Infantile] explode all trees<br><br>#14 (“Infantile Diarrhea*”):ti,ab,kw<br><br>#15 (“Childhood Diarrhea*”):ti,ab,kw<br><br>#16 #11 OR #12 OR #13 OR #14 OR #15<br><br>#17 #10 AND #16 | #18 MeSH descriptor: [Zinc] explode all trees<br><br>#19 (zinc):ti,ab,kw<br><br>#20 (Zn):ti,ab,kw<br><br>#21 #18 OR #19 OR #20<br><br>#22 #17 AND #21 515 |

Table S3: Scopus

| Diarrhea | Children up to 10 years | Zinc |
|---|---|---|
| TITLE-ABS-KEY ( ( diarrhea OR dehydrat* ) ) | ( TITLE-ABS-KEY ( ( child* OR “preschool child*” OR schoolchild* OR kid OR kids OR toddler* OR infant OR infants OR “Infancy” OR “Baby” OR “Babies”OR “ne wborn*” OR “neonat*” OR preterm OR pre matur* OR “pediatric*” OR “paediatric*” OR kinderg* ) ) | TITLE-ABS-KEY ( ( Zinc OR Zn ) ) ) |

Table S4: CINAHL

| Diarrhea | Children up to 10 years | Zinc |
|---|---|---|
| ( ((MH “Diarrhea”) OR MH Dehydration OR MH Dysentery OR MH Giardiasis OR “blood in stool” OR Dysenter* OR “Abdominal distension” OR “loose motion”) ) | ( ( Infant* OR toddler* OR baby OR babies OR preschool OR newborn OR neonate* OR kindergarten OR under-10 OR under-ten OR “under ten” OR kid OR kids OR paediatr* OR pediatr* ) ) | ( ( Zinc OR Zn ) ) |

Table S5: Clinicaltrials.gov

| Diarrhea | Children up to 10 years | Zinc |
|---|---|---|
| "Diarrhea" OR "diarrhoea" OR "diarrhea, infantile" OR "diarrhoea, infantile" OR "infantile diarrhea*" "infantile diarrhoea*" OR "childhood diarrhea*" OR "childhood diarrhoea*" | "child" OR "child, preschool" OR "child*" OR "preschool*" OR "schoolchild*" OR "school age" OR "Kid" OR "Kids" OR "toddler*" OR "infant" OR "infant*" OR "Infancy" OR "Baby" OR "Babies" OR "newborn*" OR "neonat*" OR "Preterm" OR "prematur*" OR "pediatrics" OR "pediatric*" OR "paediatric*" OR "schools, | "Zinc" OR "Zn" |

| | | |
|---|---|---|
| | nursery" OR "nursery school" OR "kindergar*" | |

Table 1A: Definition of diarrhea according to the included studies

| **Study ID** | **Definition of diarrhea** |
|---|---|
| Ahmadipour 2019 (25) | Defined as a watery stool occurring at least 3 times in a 24-hour period |
| Al Sonboli 2003 (26) | Defined as three or more watery stools in the previous 24 hours with <7 days duration. |
| Awasthi 2006 (19) | Defined as three or more loose or watery motions per day, and of less than 7 days duration |
| Bahl 2002 (17) | Defined as the passage of 5 rather than ≥3 liquid stools |
| Baqui 2002 (27) | Defined as three or more loose, liquid, or watery stools in a 24 hour period. |
| Bhandari 2008 (28) | a) In infants aged < 2 months, diarrhea was defined based on caregiver's report of recent change in consistency and/or frequency of stools;<br>b) For older children, caregiver's report of ≥ 3 loose or watery stools in a 24-hour period constituted a diarrheal illness. |
| Bhatnagar 2004 (29) | Defined as passage of three or more liquid stools daily for ≤ 72 hours |
| Boran 2005 (30) | Defined as three or more loose stools in a 24 hour period |
| Brooks 2005 (16) | Defined as passage of ≥ 3 watery stools in the preceding 24 hour period. |
| Crisinel 2015 (31) | Defined as passage of 3 or more stools per day for less than 72 hour period. |
| Dalgic 2011 (32) | Defined as passage of ≥ 3 watery or looser than normal stools within a 24 hour period. |

| | |
|---|---|
| Dutta 2000 (33) | Defined as passage of > four stools within last 24 hours (for 72 hours or less). |
| Dutta 2011 (34) | Defined as more than 3 diarrheal episodes within the last 24 hours) of less than 72 hours duration. |
| Elnemr 2007 (35) | Defined as passage of three or more loose, liquid stools in a 24 hour period. |
| FischerWalker 2006 (20) | Defined as passage of ≥ 3 watery stools in a 24 hour period. |
| FischerWalker 2007 (36) | Defined as passage of ≥ 3 loose or watery stools in a 24 hour period. |
| FischerWalker 2008 (37) | Defined as passage of > 3 loose, liquid, or watery stools in a 24 hour period. |
| Gregorio 2007 (38) | diarrhea <7 days duration and no evidence of dehydration. |
| Karamyyar 2013 (39) | Defined as acute onset of change in stool frequency and consistency lasting for fewer than 14 days and without blood in stool examination. |
| Kakar 2022 (40) | Defined as loose or watery stool passing more than three times in a 24 hour period, with or without mucus in stool. |
| Larson 2005 (41) | not mentioned |
| Mazumder 2010 (42) | In infants aged <2 months, diarrhoea was defined based on the caregiver's report of a recent change in the consistency or frequency of stools.<br><br>For children 2 months of age or older, the caregiver's report of ≥3 loose or watery stools in a 24-hour period was considered to indicate diarrhoeal illness. |
| Dhingra 2020 (21) | Defined as three or more loose or watery stools in the past 24 hour period, for less than 72 hours. |
| Patel 2013 (43) | Defined as passage of more than three unformed stools in the prior 24 hours. |
| Patel 2013 (2) (44) | Defined as passage of more than 3 unformed stools in the prior 24 hours. |

| | |
|---|---|
| Patel 2009 (45) | Defined as passage of more than three unformed stools in the prior 24 hours. |
| Patel 2015 (46) | Defined as passage of one or more loose stools in a 24 hour period. |
| Patro 2010 (47) | Defined as the passage of ≥ 3 loose stools in a 24-hour period. |
| Polat 2003 (18) | Defined as the passage of four or more watery, loose stools reported in the 24 hour period before enrolment |
| Rerksuppaphol 2020 (48) | Acute diarrhoea was defined according to the following criteria:<br>(i) patients who passed abnormal watery and/or mucous stool,<br>(ii) more than three times within the previous 24 hours,<br>(iii) for less than 2 weeks. |
| Roy 2008 (49) | Defined as passage of one or more loose stools in a 24 hour period. |
| Roy 2007 (23) | Defined as three or more liquid, watery or mucoid stools in a 24 hour period. |
| Shah 2021 (50) | Defined as passage of 3 or more loose stools in last 24 hours before enrollment. |
| Shahzad 2022 (51) | Defined as passage of three or more loose or liquid stools per day, for three or more days, and for less than 14 days. |
| Shimelis 2008 (52) | acute watery diarrhea for less than 7days |
| Strand 2002 (53) | Defined as the passage of 3 or more loose or watery stools in the 24-hour period before enrollment. |
| Trivedi 2009 (54) | Defined as passage of at least three unformed stools in a 24-hour period. |
| Valery 2005 (55) | Defined as passage of more than three loose stools in a 24-hour period. |
| Wadhwa 2011 (56) | Defined as passage of ≥ 3 loose stools per day, and at least 1 in the last 12 hours, for fewer than 7 days. |

| | |
|---|---|
| Yalcin 2022 (57) | Defined as passage of three or more watery or loose stools in 24 hours for a minimum of one day and a maximum of 14 days. |
| Yazar 2016 (58) | not mentioned |
| Wang 2016 (24) | passage of loose stools for >2 weeks, with progression to chronic diarrhea at the 4-week mark. |
| Khatun 2001 (59) | persistent diarrhea, defined as diarrhea for more than 14 d duration and having no systemic infection or clinical signs of vitamin A deficiency and having not received vitamin A supplementation within 3 mo. |

Table 2A: Definition of recovery by the included trials

| Study | Definition |
|---|---|
| **Dutta 2000 (33)** | Recovery defined as the passage of normal stool or no stool for the last 18 hours. |
| **Dutta 2011 (34)** | Recovery defined as the passage of soft stool, formed stool, or no stool for 18 hours. |
| **Karamyyar 2013 (39)** | Complete recovery defined as diarrhea discontinuation and return to the past defecation status. Relative recovery defined as decreased stool frequency to 1-2 times per day and change stool consistency from watery to soft or firm. |
| **Kakar 2022 (40)** | Recovery from diarrhea defined in term of reduction in frequency and volume of stool. Reduction in the frequency of diarrhea means passing of stools less than three times a day. |
| **Patel 2015 (46)** | Resolution of diarrhea defined as no episode of loose stool in a day. |
| **Rerksuppaphol 2020 (48)** | Recovery from diarrhea defined as the passage of two consecutive semi-formed stools or not passing stool for 12 hours since the last defecation |
| **Roy 2008 (49)** | Resolution of diarrhea defined as the passage of first formed stools or no stool for 24 hours |
| **Roy 2007 (23)** | Recovery from diarrhea defined as the passage of soft stool. |
| **Shah 2021 (50)** | Recovery from diarrhea defined as the passage of normal stool or no stool since last 18 hours. |
| **Strand 2002 (53)** | Recovery from diarrhea defined as the first of the first 2 consecutive diarrhea-free days. A diarrhea-free day was a day when the child passed 3 loose and no watery stools. |

| | |
|---|---|
| **Wadhwa 2011 (56)** | Recovery from diarrhea defined as the time of the last abnormal stool before a 12-hour period when no stool had been passed or before the passage of 2 consecutive formed stools. |
| **Wang 2016 (24)** | Significantly effective treatment defined as stool traits and frequency returned to normal, and systemic symptoms resolved. Effective treatment defined as stool traits and frequency and systemic symptoms clearly improved |
| **Khatun 2001 (59)** | Clinical recovery from persistent diarrhea defined as the time of the last diarrheal stool followed by passage of soft or formed stool for more than 48 hours of observation in the hospital. |

Table 3A: GRADE evidence profile for persistent diarrhea

| **Certainty assessment** | | | | | | | **№ of patients** | | **Effect** | | **Certainty** | **Importance** |
|---|---|---|---|---|---|---|---|---|---|---|---|---|
| **№ of studies** | **Study design** | **Risk of bias** | **Inconsistency** | **Indirectness** | **Imprecision** | **Other considerations** | **zinc** | **no zinc** | **Relative (95% CI)** | **Absolute (95% CI)** | | |
| **Recovery** | | | | | | | | | | | | |
| 2 | randomized trials | very serious[a] | not serious[b] | not serious | serious[b] | none | 49/55 (89.1%) | 29/57 (50.9%) | **RR 1.75** (1.34 to 2.30) | **382 more per 1,000** (from 173 more to 661 more) | ⨁◯◯◯ Very low | CRITICAL |
| **Mortality** | | | | | | | | | | | | |
| 2 | randomized trials | serious[c] | not serious | not serious | serious[b] | none | 6/102 (5.9%) | 7/100 (7.0%) | **RR 0.84** (0.29 to 2.37) | **11 fewer per 1,000** (from 50 fewer to 96 more) | ⨁⨁◯◯ Low | IMPORTANT |
| **Duration of diarrhea** | | | | | | | | | | | | |
| 2 | randomized trials | serious[c] | serious[d] | not serious | serious[b] | none | 122 | 120 | - | MD **26.29 lower** (47.35 lower to 5.23 lower) | ⨁◯◯◯ Very low | CRITICAL |

**CI:** confidence interval; **MD:** mean difference; **RR:** risk ratio

**Explanations**

a. 1 study had high risk of bias
b. small sample size
c. Studies included had some concerns
d. heterogeneity p value = 0.004 and I2 88%

# Zinc vs no zinc for acute diarrhea (subgroups)

## Recovery

Figure 1A

Outcome 1: Recovery from diarrhea (subgroup based on definition of diarrhea)

| Study or Subgroup | Experimental Events | Experimental Total | Control Events | Control Total | Weight | Risk Ratio M-H, Random, 95% CI |
|---|---|---|---|---|---|---|
| **13.4.1 WHO definition** | | | | | | |
| Awasthi 2006 | 773 | 998 | 724 | 984 | 7.3% | 1.05 [1.00, 1.11] |
| Bhatnagar 2004 | 131 | 132 | 125 | 134 | 7.5% | 1.06 [1.01, 1.12] |
| Crisinel 2015 | 35 | 37 | 31 | 39 | 2.2% | 1.19 [1.00, 1.42] |
| Dutta 2011 | 38 | 41 | 29 | 43 | 1.5% | 1.37 [1.10, 1.72] |
| FischerWalker 2006 | 403 | 538 | 427 | 536 | 6.4% | 0.94 [0.88, 1.00] |
| Kakar 2022 | 72 | 72 | 68 | 72 | 6.6% | 1.06 [0.99, 1.13] |
| Patel 2009 | 228 | 248 | 234 | 247 | 7.5% | 0.97 [0.93, 1.02] |
| Patro 2010 | 68 | 69 | 69 | 72 | 7.0% | 1.03 [0.97, 1.09] |
| Rerksuppaphol 2020 | 43 | 43 | 42 | 43 | 6.5% | 1.02 [0.96, 1.09] |
| Shah 2021 | 45 | 45 | 39 | 45 | 3.7% | 1.15 [1.02, 1.30] |
| Strand 2002 (1) | 207 | 223 | 195 | 224 | 6.6% | 1.07 [1.00, 1.13] |
| Strand 2002 (2) | 205 | 221 | 196 | 225 | 6.6% | 1.06 [1.00, 1.13] |
| **Subtotal (95% CI)** | | **2667** | | **2664** | **69.4%** | **1.04 [1.01, 1.08]** |
| Total events | 2248 | | 2179 | | | |
| Heterogeneity: Tau² = 0.00; Chi² = 31.26, df = 11 (P = 0.001); I² = 65% | | | | | | |
| Test for overall effect: Z = 2.59 (P = 0.010) | | | | | | |
| **13.4.2 Other definition** | | | | | | |
| Bahl 2002 (3) | 193 | 202 | 186 | 200 | 7.4% | 1.03 [0.98, 1.08] |
| Bahl 2002 (4) | 192 | 201 | 187 | 201 | 7.4% | 1.03 [0.98, 1.08] |
| Dutta 2000 | 44 | 44 | 32 | 36 | 3.6% | 1.13 [0.99, 1.27] |
| Patel 2015 | 43 | 43 | 44 | 53 | 3.5% | 1.20 [1.06, 1.36] |
| Polat 2003 (5) | 35 | 40 | 20 | 36 | 0.8% | 1.57 [1.15, 2.16] |
| Polat 2003 (6) | 44 | 52 | 34 | 54 | 1.4% | 1.34 [1.06, 1.70] |
| Shimelis 2008 | 99 | 179 | 121 | 222 | 2.2% | 1.01 [0.85, 1.21] |
| **Subtotal (95% CI)** | | **761** | | **802** | **26.3%** | **1.11 [1.03, 1.19]** |
| Total events | 650 | | 624 | | | |
| Heterogeneity: Tau² = 0.01; Chi² = 20.32, df = 6 (P = 0.002); I² = 70% | | | | | | |
| Test for overall effect: Z = 2.68 (P = 0.007) | | | | | | |
| **13.4.3 Unspecified** | | | | | | |
| Gregorio 2007 | 54 | 59 | 43 | 57 | 2.4% | 1.21 [1.03, 1.43] |
| Yazar 2016 | 49 | 55 | 39 | 55 | 1.9% | 1.26 [1.04, 1.52] |
| **Subtotal (95% CI)** | | **114** | | **112** | **4.3%** | **1.23 [1.09, 1.40]** |
| Total events | 103 | | 82 | | | |
| Heterogeneity: Tau² = 0.00; Chi² = 0.07, df = 1 (P = 0.79); I² = 0% | | | | | | |
| Test for overall effect: Z = 3.23 (P = 0.001) | | | | | | |
| **Total (95% CI)** | | **3542** | | **3578** | **100.0%** | **1.07 [1.03, 1.10]** |
| Total events | 3001 | | 2885 | | | |
| Heterogeneity: Tau² = 0.00; Chi² = 56.89, df = 20 (P < 0.0001); I² = 65% | | | | | | |
| Test for overall effect: Z = 4.13 (P < 0.0001) | | | | | | |
| Test for subgroup differences: Chi² = 7.41, df = 2 (P = 0.02), I² = 73.0% | | | | | | |

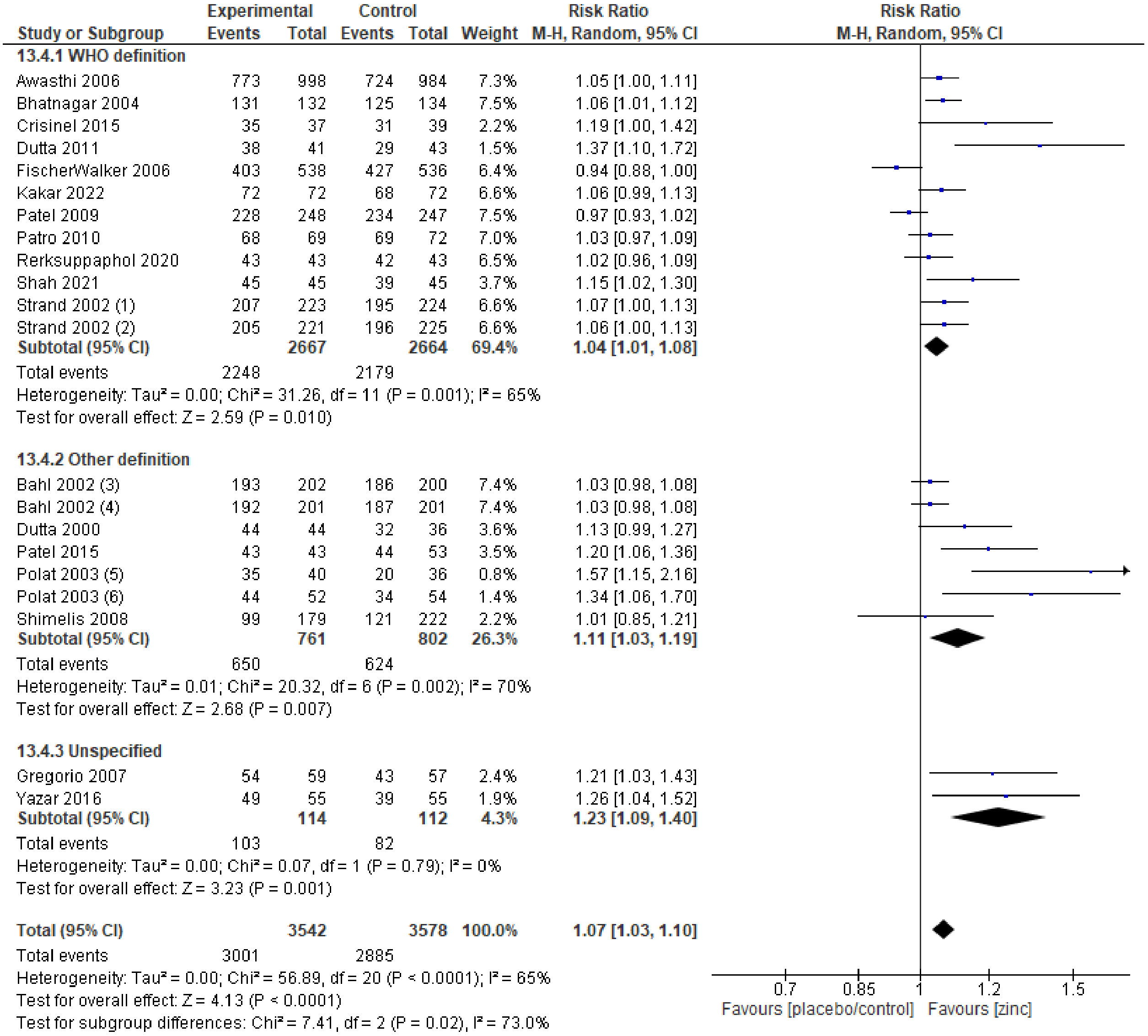


Footnotes

(1) caretakers

(2) fieldworkers

(3) zinc syrup

(4) zinc ORS

(5) low serum zinc levels

(6) normal serum zinc levels

Figure 1B

## Outcome 1: Recovery from diarrhea (subgroups based on dose of zinc administered)

| Study or Subgroup | Experimental Events | Experimental Total | Control Events | Control Total | Weight | Risk Ratio M-H, Random, 95% CI |
|---|---|---|---|---|---|---|
| **3.3.1 10mg** | | | | | | |
| FischerWalker 2006 | 403 | 538 | 427 | 536 | 6.4% | 0.94 [0.88, 1.00] |
| **Subtotal (95% CI)** | | **538** | | **536** | **6.4%** | **0.94 [0.88, 1.00]** |
| Total events | 403 | | 427 | | | |
| Heterogeneity: Not applicable | | | | | | |
| Test for overall effect: Z = 1.86 (P = 0.06) | | | | | | |
| **3.3.2 15mg** | | | | | | |
| Rerksuppaphol 2020 | 43 | 43 | 42 | 43 | 6.5% | 1.02 [0.96, 1.09] |
| Yazar 2016 | 49 | 55 | 39 | 55 | 1.9% | 1.26 [1.04, 1.52] |
| **Subtotal (95% CI)** | | **98** | | **98** | **8.4%** | **1.13 [0.80, 1.58]** |
| Total events | 92 | | 81 | | | |
| Heterogeneity: Tau$^2$ = 0.05; Chi$^2$ = 11.11, df = 1 (P = 0.0009); I$^2$ = 91% | | | | | | |
| Test for overall effect: Z = 0.69 (P = 0.49) | | | | | | |
| **3.3.3 20mg** | | | | | | |
| Dutta 2011 | 38 | 41 | 29 | 43 | 1.5% | 1.37 [1.10, 1.72] |
| Gregorio 2007 | 54 | 59 | 43 | 57 | 2.4% | 1.21 [1.03, 1.43] |
| Polat 2003 (1) | 35 | 40 | 20 | 36 | 0.8% | 1.57 [1.15, 2.16] |
| Polat 2003 (2) | 44 | 52 | 34 | 54 | 1.4% | 1.34 [1.06, 1.70] |
| Shah 2021 | 45 | 45 | 39 | 45 | 3.7% | 1.15 [1.02, 1.30] |
| Shimelis 2008 | 99 | 179 | 121 | 222 | 2.2% | 1.01 [0.85, 1.21] |
| **Subtotal (95% CI)** | | **416** | | **457** | **12.0%** | **1.22 [1.10, 1.36]** |
| Total events | 315 | | 286 | | | |
| Heterogeneity: Tau$^2$ = 0.01; Chi$^2$ = 9.08, df = 5 (P = 0.11); I$^2$ = 45% | | | | | | |
| Test for overall effect: Z = 3.72 (P = 0.0002) | | | | | | |
| **3.3.4 40mg** | | | | | | |
| Dutta 2000 | 44 | 44 | 32 | 36 | 3.6% | 1.13 [0.99, 1.27] |
| **Subtotal (95% CI)** | | **44** | | **36** | **3.6%** | **1.13 [0.99, 1.27]** |
| Total events | 44 | | 32 | | | |
| Heterogeneity: Not applicable | | | | | | |
| Test for overall effect: Z = 1.88 (P = 0.06) | | | | | | |
| **3.3.5 Dose by weight or age** | | | | | | |
| Bahl 2002 (3) | 193 | 202 | 186 | 200 | 7.4% | 1.03 [0.98, 1.08] |
| Bahl 2002 (4) | 192 | 201 | 187 | 201 | 7.4% | 1.03 [0.98, 1.08] |
| Bhatnagar 2004 | 131 | 132 | 125 | 134 | 7.5% | 1.06 [1.01, 1.12] |
| Crisinel 2015 | 35 | 37 | 31 | 39 | 2.2% | 1.19 [1.00, 1.42] |
| Patel 2009 | 228 | 248 | 234 | 247 | 7.5% | 0.97 [0.93, 1.02] |
| Patel 2015 | 43 | 43 | 44 | 53 | 3.5% | 1.20 [1.06, 1.36] |
| Patro 2010 | 68 | 69 | 69 | 72 | 7.0% | 1.03 [0.97, 1.09] |
| Strand 2002 (5) | 207 | 223 | 195 | 224 | 6.6% | 1.07 [1.00, 1.13] |
| Strand 2002 (6) | 205 | 221 | 196 | 225 | 6.6% | 1.06 [1.00, 1.13] |
| **Subtotal (95% CI)** | | **1376** | | **1395** | **55.7%** | **1.04 [1.01, 1.08]** |
| Total events | 1302 | | 1267 | | | |
| Heterogeneity: Tau$^2$ = 0.00; Chi$^2$ = 18.19, df = 8 (P = 0.02); I$^2$ = 56% | | | | | | |
| Test for overall effect: Z = 2.75 (P = 0.006) | | | | | | |
| **3.3.6 Unspecified** | | | | | | |
| Awasthi 2006 (7) | 773 | 998 | 724 | 984 | 7.3% | 1.05 [1.00, 1.11] |
| Kakar 2022 | 72 | 72 | 68 | 72 | 6.6% | 1.06 [0.99, 1.13] |
| **Subtotal (95% CI)** | | **1070** | | **1056** | **13.9%** | **1.05 [1.01, 1.10]** |
| Total events | 845 | | 792 | | | |
| Heterogeneity: Tau$^2$ = 0.00; Chi$^2$ = 0.02, df = 1 (P = 0.87); I$^2$ = 0% | | | | | | |
| Test for overall effect: Z = 2.69 (P = 0.007) | | | | | | |
| **Total (95% CI)** | | **3542** | | **3578** | **100.0%** | **1.07 [1.03, 1.10]** |
| Total events | 3001 | | 2885 | | | |
| Heterogeneity: Tau$^2$ = 0.00; Chi$^2$ = 56.89, df = 20 (P < 0.0001); I$^2$ = 65% | | | | | | |
| Test for overall effect: Z = 4.13 (P < 0.0001) | | | | | | |
| Test for subgroup differences: Chi$^2$ = 20.40, df = 5 (P = 0.001), I$^2$ = 75.5% | | | | | | |

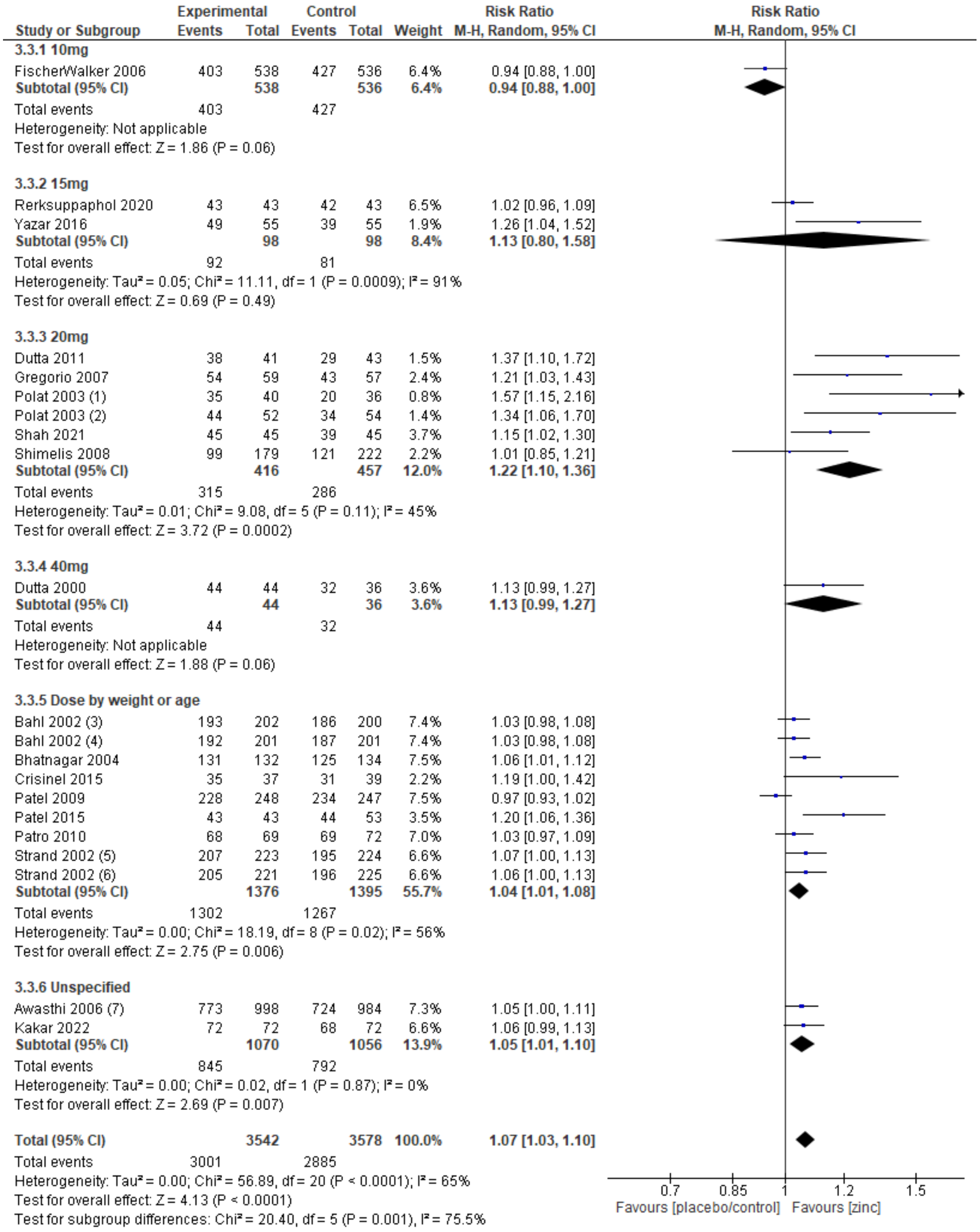


Footnotes
(1) low serum zinc levels
(2) normal serum zinc levels
(3) zinc syrup
(4) zinc ORS
(5) caretakers
(6) fieldworkers
(7) Ethiopia and India gave 20mg whereas other sites gave 10mg

Figure 1C

## Outcome 1: Recovery from diarrhea (based on duration of zinc supplementation)

| Study or Subgroup | Experimental Events | Experimental Total | Control Events | Control Total | Weight | Risk Ratio M-H, Random, 95% CI |
|---|---|---|---|---|---|---|
| **8.5.1 <=5d** | | | | | | |
| Dutta 2000 | 44 | 44 | 32 | 36 | 3.6% | 1.13 [0.99, 1.27] |
| Yazar 2016 | 49 | 55 | 39 | 55 | 1.9% | 1.26 [1.04, 1.52] |
| **Subtotal (95% CI)** | | **99** | | **91** | **5.5%** | **1.17 [1.04, 1.31]** |
| Total events | 93 | | 71 | | | |
| Heterogeneity: Tau$^2$ = 0.00; Chi$^2$ = 1.13, df = 1 (P = 0.29); I$^2$ = 12% | | | | | | |
| Test for overall effect: Z = 2.64 (P = 0.008) | | | | | | |
| **8.5.2 <=10d** | | | | | | |
| Crisinel 2015 | 35 | 37 | 31 | 39 | 2.2% | 1.19 [1.00, 1.42] |
| Patro 2010 | 68 | 69 | 69 | 72 | 7.0% | 1.03 [0.97, 1.09] |
| **Subtotal (95% CI)** | | **106** | | **111** | **9.2%** | **1.09 [0.91, 1.31]** |
| Total events | 103 | | 100 | | | |
| Heterogeneity: Tau$^2$ = 0.01; Chi$^2$ = 3.95, df = 1 (P = 0.05); I$^2$ = 75% | | | | | | |
| Test for overall effect: Z = 0.93 (P = 0.35) | | | | | | |
| **8.5.3 14d** | | | | | | |
| Awasthi 2006 | 773 | 998 | 724 | 984 | 7.3% | 1.05 [1.00, 1.11] |
| Bahl 2002 (1) | 193 | 202 | 186 | 200 | 7.4% | 1.03 [0.98, 1.08] |
| Bahl 2002 (2) | 192 | 201 | 187 | 201 | 7.4% | 1.03 [0.98, 1.08] |
| Bhatnagar 2004 | 131 | 132 | 125 | 134 | 7.5% | 1.06 [1.01, 1.12] |
| Dutta 2011 | 38 | 41 | 29 | 43 | 1.5% | 1.37 [1.10, 1.72] |
| FischerWalker 2006 | 403 | 538 | 427 | 536 | 6.4% | 0.94 [0.88, 1.00] |
| Gregorio 2007 | 54 | 59 | 43 | 57 | 2.4% | 1.21 [1.03, 1.43] |
| Patel 2009 | 228 | 248 | 234 | 247 | 7.5% | 0.97 [0.93, 1.02] |
| Patel 2015 | 43 | 43 | 44 | 53 | 3.5% | 1.20 [1.06, 1.36] |
| Polat 2003 (3) | 44 | 52 | 34 | 54 | 1.4% | 1.34 [1.06, 1.70] |
| Polat 2003 (4) | 35 | 40 | 20 | 36 | 0.8% | 1.57 [1.15, 2.16] |
| Shah 2021 | 45 | 45 | 39 | 45 | 3.7% | 1.15 [1.02, 1.30] |
| Shimelis 2008 | 99 | 179 | 121 | 222 | 2.2% | 1.01 [0.85, 1.21] |
| **Subtotal (95% CI)** | | **2778** | | **2812** | **59.0%** | **1.07 [1.02, 1.12]** |
| Total events | 2278 | | 2213 | | | |
| Heterogeneity: Tau$^2$ = 0.00; Chi$^2$ = 46.97, df = 12 (P < 0.00001); I$^2$ = 74% | | | | | | |
| Test for overall effect: Z = 2.93 (P = 0.003) | | | | | | |
| **8.5.4 unspecified** | | | | | | |
| Kakar 2022 | 72 | 72 | 68 | 72 | 6.6% | 1.06 [0.99, 1.13] |
| Rerksuppaphol 2020 | 43 | 43 | 42 | 43 | 6.5% | 1.02 [0.96, 1.09] |
| Strand 2002 (5) | 205 | 221 | 196 | 225 | 6.6% | 1.06 [1.00, 1.13] |
| Strand 2002 (6) | 207 | 223 | 195 | 224 | 6.6% | 1.07 [1.00, 1.13] |
| **Subtotal (95% CI)** | | **559** | | **564** | **26.3%** | **1.05 [1.02, 1.09]** |
| Total events | 527 | | 501 | | | |
| Heterogeneity: Tau$^2$ = 0.00; Chi$^2$ = 1.26, df = 3 (P = 0.74); I$^2$ = 0% | | | | | | |
| Test for overall effect: Z = 3.27 (P = 0.001) | | | | | | |
| **Total (95% CI)** | | **3542** | | **3578** | **100.0%** | **1.07 [1.03, 1.10]** |
| Total events | 3001 | | 2885 | | | |
| Heterogeneity: Tau$^2$ = 0.00; Chi$^2$ = 56.89, df = 20 (P < 0.0001); I$^2$ = 65% | | | | | | |
| Test for overall effect: Z = 4.13 (P < 0.0001) | | | | | | |
| Test for subgroup differences: Chi$^2$ = 3.00, df = 3 (P = 0.39), I$^2$ = 0.1% | | | | | | |

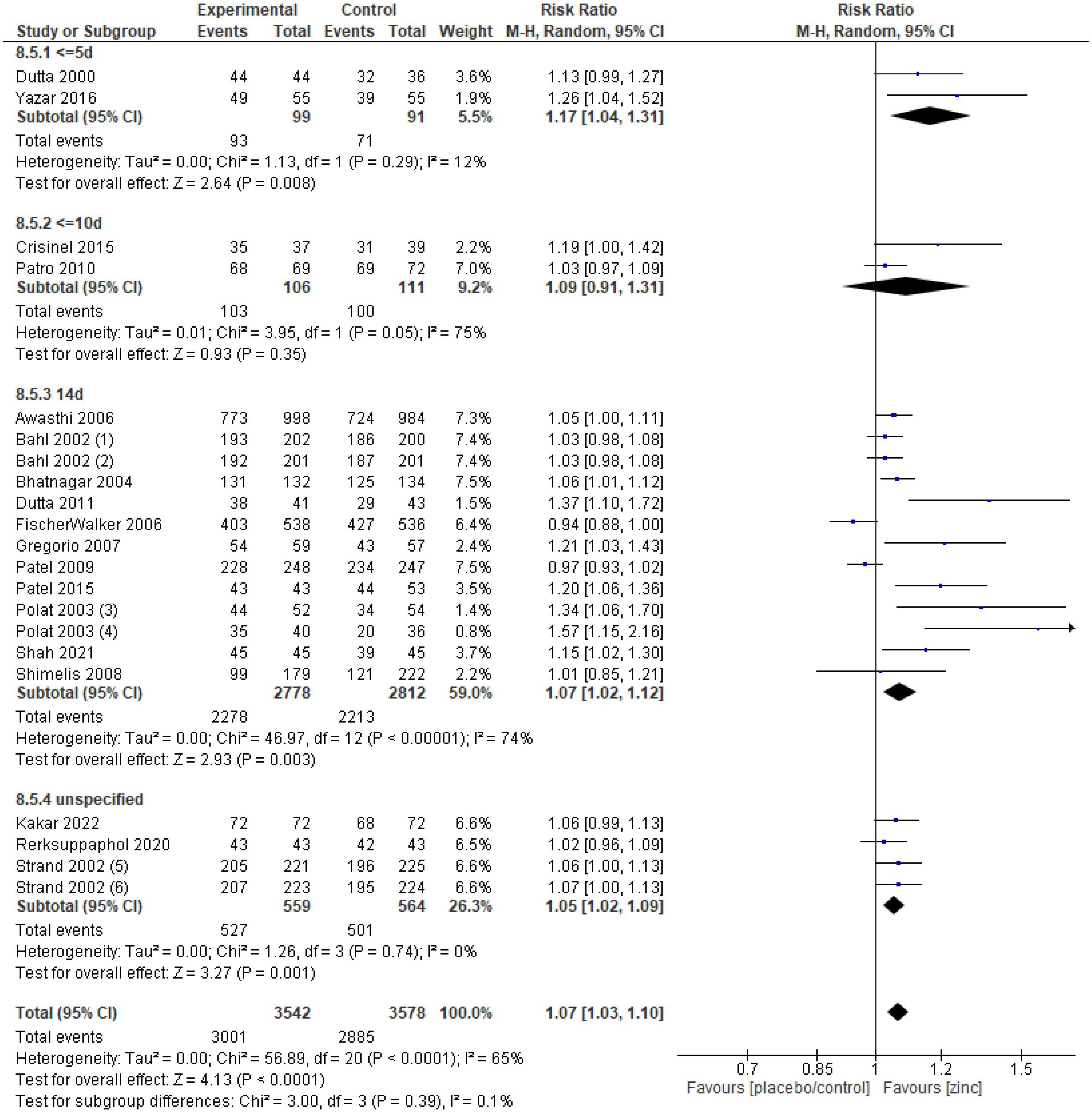


Footnotes
(1) zinc syrup
(2) zinc ORS
(3) normal serum zinc levels
(4) low serum zinc levels
(5) fieldworkers
(6) caretakers

Figure 1D

## Outcome 1: Recovery from diarrhea (based on zinc formulation)

| Study or Subgroup | Experimental Events | Experimental Total | Control Events | Control Total | Weight | Risk Ratio M-H, Random, 95% CI |
|---|---|---|---|---|---|---|
| **4.3.1 Zinc sulfate** | | | | | | |
| Bhatnagar 2004 | 131 | 132 | 125 | 134 | 7.5% | 1.06 [1.01, 1.12] |
| Crisinel 2015 | 35 | 37 | 31 | 39 | 2.2% | 1.19 [1.00, 1.42] |
| Dutta 2000 | 44 | 44 | 32 | 36 | 3.6% | 1.13 [0.99, 1.27] |
| Gregorio 2007 | 54 | 59 | 43 | 57 | 2.4% | 1.21 [1.03, 1.43] |
| Patel 2009 | 228 | 248 | 234 | 247 | 7.5% | 0.97 [0.93, 1.02] |
| Patel 2015 | 43 | 43 | 44 | 53 | 3.5% | 1.20 [1.06, 1.36] |
| Patro 2010 | 68 | 69 | 69 | 72 | 7.0% | 1.03 [0.97, 1.09] |
| Polat 2003 (1) | 35 | 40 | 20 | 36 | 0.8% | 1.57 [1.15, 2.16] |
| Polat 2003 (2) | 44 | 52 | 34 | 54 | 1.4% | 1.34 [1.06, 1.70] |
| **Subtotal (95% CI)** | | **724** | | **728** | **35.8%** | **1.12 [1.04, 1.20]** |
| Total events | 682 | | 632 | | | |
| Heterogeneity: Tau² = 0.01; Chi² = 40.03, df = 8 (P < 0.00001); I² = 80% | | | | | | |
| Test for overall effect: Z = 3.07 (P = 0.002) | | | | | | |
| **4.3.2 Zinc gluconate** | | | | | | |
| Bahl 2002 (3) | 193 | 202 | 186 | 200 | 7.4% | 1.03 [0.98, 1.08] |
| Bahl 2002 (4) | 192 | 201 | 187 | 201 | 7.4% | 1.03 [0.98, 1.08] |
| Strand 2002 (5) | 207 | 223 | 195 | 224 | 6.6% | 1.07 [1.00, 1.13] |
| Strand 2002 (6) | 205 | 221 | 196 | 225 | 6.6% | 1.06 [1.00, 1.13] |
| **Subtotal (95% CI)** | | **847** | | **850** | **28.1%** | **1.04 [1.01, 1.07]** |
| Total events | 797 | | 764 | | | |
| Heterogeneity: Tau² = 0.00; Chi² = 1.82, df = 3 (P = 0.61); I² = 0% | | | | | | |
| Test for overall effect: Z = 2.94 (P = 0.003) | | | | | | |
| **4.3.3 Zinc bisglycinate** | | | | | | |
| Rerksuppaphol 2020 | 43 | 43 | 42 | 43 | 6.5% | 1.02 [0.96, 1.09] |
| **Subtotal (95% CI)** | | **43** | | **43** | **6.5%** | **1.02 [0.96, 1.09]** |
| Total events | 43 | | 42 | | | |
| Heterogeneity: Not applicable | | | | | | |
| Test for overall effect: Z = 0.71 (P = 0.48) | | | | | | |
| **4.3.4 Unspecified** | | | | | | |
| Awasthi 2006 | 773 | 998 | 724 | 984 | 7.3% | 1.05 [1.00, 1.11] |
| Dutta 2011 | 38 | 41 | 29 | 43 | 1.5% | 1.37 [1.10, 1.72] |
| FischerWalker 2006 | 403 | 538 | 427 | 536 | 6.4% | 0.94 [0.88, 1.00] |
| Kakar 2022 | 72 | 72 | 68 | 72 | 6.6% | 1.06 [0.99, 1.13] |
| Shah 2021 | 45 | 45 | 39 | 45 | 3.7% | 1.15 [1.02, 1.30] |
| Shimelis 2008 | 99 | 179 | 121 | 222 | 2.2% | 1.01 [0.85, 1.21] |
| Yazar 2016 | 49 | 55 | 39 | 55 | 1.9% | 1.26 [1.04, 1.52] |
| **Subtotal (95% CI)** | | **1928** | | **1957** | **29.7%** | **1.08 [1.00, 1.16]** |
| Total events | 1479 | | 1447 | | | |
| Heterogeneity: Tau² = 0.01; Chi² = 22.62, df = 6 (P = 0.0009); I² = 73% | | | | | | |
| Test for overall effect: Z = 2.02 (P = 0.04) | | | | | | |
| **Total (95% CI)** | | **3542** | | **3578** | **100.0%** | **1.07 [1.03, 1.10]** |
| Total events | 3001 | | 2885 | | | |
| Heterogeneity: Tau² = 0.00; Chi² = 56.89, df = 20 (P < 0.0001); I² = 65% | | | | | | |
| Test for overall effect: Z = 4.13 (P < 0.0001) | | | | | | |
| Test for subgroup differences: Chi² = 4.52, df = 3 (P = 0.21), I² = 33.7% | | | | | | |

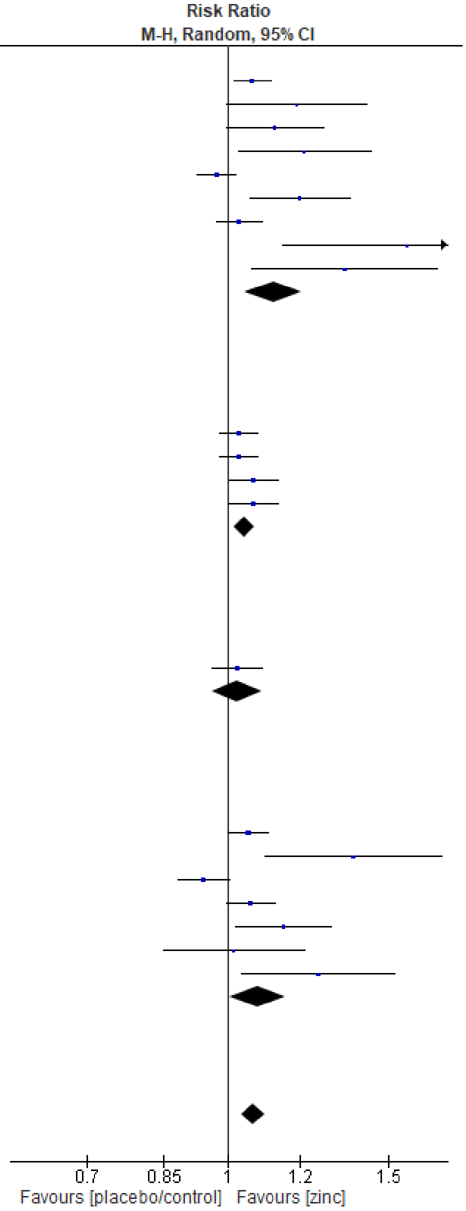


Footnotes
(1) low serum zinc levels
(2) normal serum zinc levels
(3) zinc syrup
(4) zinc ORS
(5) caretakers
(6) fieldworkers

Figure 1E

## Outcome1: Recovery from diarrhea (based on World Bank Income Classification)

| Study or Subgroup | Experimental Events | Experimental Total | Control Events | Control Total | Weight | Risk Ratio M-H, Random, 95% CI |
|---|---|---|---|---|---|---|
| **2.3.1 LMIC** | | | | | | |
| Bahl 2002 (1) | 193 | 202 | 186 | 200 | 7.4% | 1.03 [0.98, 1.08] |
| Bahl 2002 (2) | 192 | 201 | 187 | 201 | 7.4% | 1.03 [0.98, 1.08] |
| Bhatnagar 2004 | 131 | 132 | 125 | 134 | 7.5% | 1.06 [1.01, 1.12] |
| Dutta 2000 | 44 | 44 | 32 | 36 | 3.6% | 1.13 [0.99, 1.27] |
| Dutta 2011 | 38 | 41 | 29 | 43 | 1.5% | 1.37 [1.10, 1.72] |
| FischerWalker 2006 | 403 | 538 | 427 | 536 | 6.4% | 0.94 [0.88, 1.00] |
| Gregorio 2007 | 54 | 59 | 43 | 57 | 2.4% | 1.21 [1.03, 1.43] |
| Kakar 2022 | 72 | 72 | 68 | 72 | 6.6% | 1.06 [0.99, 1.13] |
| Patel 2009 | 228 | 248 | 234 | 247 | 7.5% | 0.97 [0.93, 1.02] |
| Patel 2015 | 43 | 43 | 44 | 53 | 3.5% | 1.20 [1.06, 1.36] |
| Polat 2003 (3) | 35 | 40 | 20 | 36 | 0.8% | 1.57 [1.15, 2.16] |
| Polat 2003 (4) | 44 | 52 | 34 | 54 | 1.4% | 1.34 [1.06, 1.70] |
| Shah 2021 | 45 | 45 | 39 | 45 | 3.7% | 1.15 [1.02, 1.30] |
| Shimelis 2008 | 99 | 179 | 121 | 222 | 2.2% | 1.01 [0.85, 1.21] |
| Strand 2002 (5) | 207 | 223 | 195 | 224 | 6.6% | 1.07 [1.00, 1.13] |
| Strand 2002 (6) | 205 | 221 | 196 | 225 | 6.6% | 1.06 [1.00, 1.13] |
| **Subtotal (95% CI)** | | **2340** | | **2385** | **75.1%** | **1.07 [1.03, 1.11]** |
| Total events | 2033 | | 1980 | | | |
| Heterogeneity: Tau² = 0.00; Chi² = 50.03, df = 15 (P < 0.0001); I² = 70% | | | | | | |
| Test for overall effect: Z = 3.50 (P = 0.0005) | | | | | | |
| **2.3.2 HIC** | | | | | | |
| Crisinel 2015 | 35 | 37 | 31 | 39 | 2.2% | 1.19 [1.00, 1.42] |
| Patro 2010 | 68 | 69 | 69 | 72 | 7.0% | 1.03 [0.97, 1.09] |
| Rerksuppaphol 2020 | 43 | 43 | 42 | 43 | 6.5% | 1.02 [0.96, 1.09] |
| Yazar 2016 | 49 | 55 | 39 | 55 | 1.9% | 1.26 [1.04, 1.52] |
| **Subtotal (95% CI)** | | **204** | | **209** | **17.6%** | **1.09 [0.98, 1.21]** |
| Total events | 195 | | 181 | | | |
| Heterogeneity: Tau² = 0.01; Chi² = 13.99, df = 3 (P = 0.003); I² = 79% | | | | | | |
| Test for overall effect: Z = 1.59 (P = 0.11) | | | | | | |
| **2.3.3 Mixed** | | | | | | |
| Awasthi 2006 | 773 | 998 | 724 | 984 | 7.3% | 1.05 [1.00, 1.11] |
| **Subtotal (95% CI)** | | **998** | | **984** | **7.3%** | **1.05 [1.00, 1.11]** |
| Total events | 773 | | 724 | | | |
| Heterogeneity: Not applicable | | | | | | |
| Test for overall effect: Z = 2.00 (P = 0.05) | | | | | | |
| **Total (95% CI)** | | **3542** | | **3578** | **100.0%** | **1.07 [1.03, 1.10]** |
| Total events | 3001 | | 2885 | | | |
| Heterogeneity: Tau² = 0.00; Chi² = 56.89, df = 20 (P < 0.0001); I² = 65% | | | | | | |
| Test for overall effect: Z = 4.13 (P < 0.0001) | | | | | | |
| Test for subgroup differences: Chi² = 0.44, df = 2 (P = 0.80), I² = 0% | | | | | | |

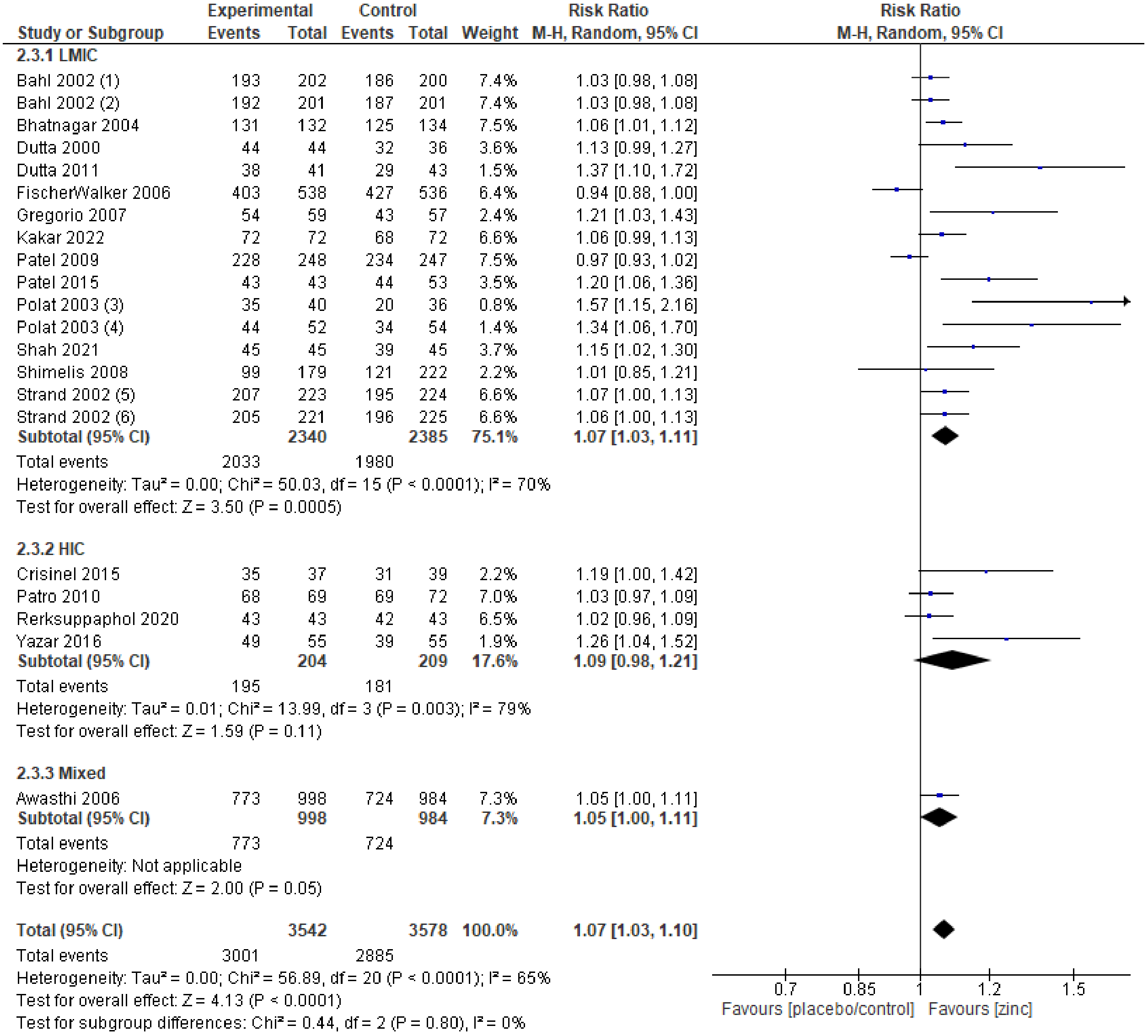


Footnotes
(1) zinc syrup
(2) zinc ORS
(3) low serum zinc levels
(4) normal serum zinc levels
(5) caretakers
(6) fieldworkers

Figure 1F

## Outcome 1: Recovery from diarrhea (based on form of zinc given)

| Study or Subgroup | Experimental Events | Experimental Total | Control Events | Control Total | Weight | Risk Ratio M-H, Random, 95% CI |
|---|---|---|---|---|---|---|
| **5.3.1 Syrup** | | | | | | |
| Bahl 2002 (1) | 193 | 202 | 186 | 200 | 7.4% | 1.03 [0.98, 1.08] |
| Bhatnagar 2004 | 131 | 132 | 125 | 134 | 7.5% | 1.06 [1.01, 1.12] |
| Dutta 2000 | 44 | 44 | 32 | 36 | 3.6% | 1.13 [0.99, 1.27] |
| Patel 2009 | 228 | 248 | 234 | 247 | 7.5% | 0.97 [0.93, 1.02] |
| Patro 2010 | 68 | 69 | 69 | 72 | 7.0% | 1.03 [0.97, 1.09] |
| Polat 2003 (2) | 35 | 40 | 20 | 36 | 0.8% | 1.57 [1.15, 2.16] |
| Polat 2003 (3) | 44 | 52 | 34 | 54 | 1.4% | 1.34 [1.06, 1.70] |
| Strand 2002 (4) | 205 | 221 | 196 | 225 | 6.6% | 1.06 [1.00, 1.13] |
| Strand 2002 (5) | 207 | 223 | 195 | 224 | 6.6% | 1.07 [1.00, 1.13] |
| Yazar 2016 | 49 | 55 | 39 | 55 | 1.9% | 1.26 [1.04, 1.52] |
| **Subtotal (95% CI)** | | **1286** | | **1283** | **50.3%** | **1.07 [1.02, 1.12]** |
| Total events | 1204 | | 1130 | | | |
| Heterogeneity: Tau² = 0.00; Chi² = 31.25, df = 9 (P = 0.0003); I² = 71% | | | | | | |
| Test for overall effect: Z = 2.81 (P = 0.005) | | | | | | |
| **5.3.2 ORS** | | | | | | |
| Bahl 2002 (6) | 192 | 201 | 187 | 201 | 7.4% | 1.03 [0.98, 1.08] |
| **Subtotal (95% CI)** | | **201** | | **201** | **7.4%** | **1.03 [0.98, 1.08]** |
| Total events | 192 | | 187 | | | |
| Heterogeneity: Not applicable | | | | | | |
| Test for overall effect: Z = 1.07 (P = 0.28) | | | | | | |
| **5.3.3 Dispersible tablet** | | | | | | |
| Awasthi 2006 | 773 | 998 | 724 | 984 | 7.3% | 1.05 [1.00, 1.11] |
| Crisinel 2015 | 35 | 37 | 31 | 39 | 2.2% | 1.19 [1.00, 1.42] |
| FischerWalker 2006 | 403 | 538 | 427 | 536 | 6.4% | 0.94 [0.88, 1.00] |
| Gregorio 2007 | 54 | 59 | 43 | 57 | 2.4% | 1.21 [1.03, 1.43] |
| Shimelis 2008 | 99 | 179 | 121 | 222 | 2.2% | 1.01 [0.85, 1.21] |
| **Subtotal (95% CI)** | | **1811** | | **1838** | **20.5%** | **1.06 [0.97, 1.15]** |
| Total events | 1364 | | 1346 | | | |
| Heterogeneity: Tau² = 0.01; Chi² = 14.54, df = 4 (P = 0.006); I² = 72% | | | | | | |
| Test for overall effect: Z = 1.19 (P = 0.23) | | | | | | |
| **5.3.4 Powder sachet** | | | | | | |
| Rerksuppaphol 2020 | 43 | 43 | 42 | 43 | 6.5% | 1.02 [0.96, 1.09] |
| **Subtotal (95% CI)** | | **43** | | **43** | **6.5%** | **1.02 [0.96, 1.09]** |
| Total events | 43 | | 42 | | | |
| Heterogeneity: Not applicable | | | | | | |
| Test for overall effect: Z = 0.71 (P = 0.48) | | | | | | |
| **5.3.5 Unspecified** | | | | | | |
| Dutta 2011 | 38 | 41 | 29 | 43 | 1.5% | 1.37 [1.10, 1.72] |
| Kakar 2022 | 72 | 72 | 68 | 72 | 6.6% | 1.06 [0.99, 1.13] |
| Patel 2015 | 43 | 43 | 44 | 53 | 3.5% | 1.20 [1.06, 1.36] |
| Shah 2021 | 45 | 45 | 39 | 45 | 3.7% | 1.15 [1.02, 1.30] |
| **Subtotal (95% CI)** | | **201** | | **213** | **15.3%** | **1.16 [1.04, 1.31]** |
| Total events | 198 | | 180 | | | |
| Heterogeneity: Tau² = 0.01; Chi² = 11.19, df = 3 (P = 0.01); I² = 73% | | | | | | |
| Test for overall effect: Z = 2.55 (P = 0.01) | | | | | | |
| **Total (95% CI)** | | **3542** | | **3578** | **100.0%** | **1.07 [1.03, 1.10]** |
| Total events | 3001 | | 2885 | | | |
| Heterogeneity: Tau² = 0.00; Chi² = 56.89, df = 20 (P < 0.0001); I² = 65% | | | | | | |
| Test for overall effect: Z = 4.13 (P < 0.0001) | | | | | | |
| Test for subgroup differences: Chi² = 4.91, df = 4 (P = 0.30), I² = 18.5% | | | | | | |

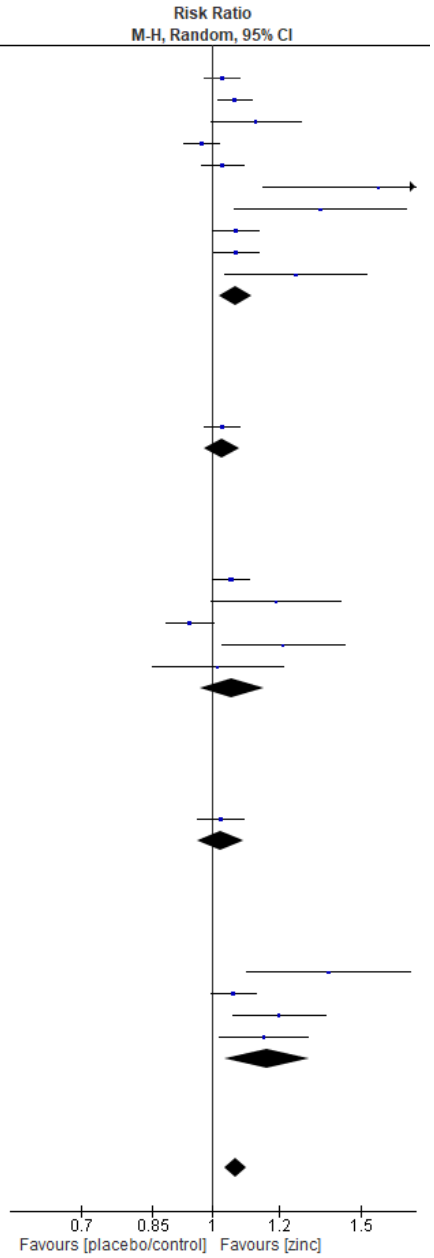


Footnotes
(1) zinc syrup
(2) low serum zinc levels
(3) normal serum zinc levels
(4) fieldworkers
(5) caretakers
(6) zinc ORS

Figure 1G

## Outcome 1: Recovery from diarrhea (based on age)

| Study or Subgroup | Experimental Events | Experimental Total | Control Events | Control Total | Weight | Risk Ratio M-H, Random, 95% CI |
|---|---|---|---|---|---|---|
| **6.3.1 >6months** | | | | | | |
| Bahl 2002 (1) | 192 | 201 | 187 | 201 | 7.4% | 1.03 [0.98, 1.08] |
| Bahl 2002 (2) | 193 | 202 | 186 | 200 | 7.4% | 1.03 [0.98, 1.08] |
| Dutta 2011 | 38 | 41 | 29 | 43 | 1.5% | 1.37 [1.10, 1.72] |
| Patel 2009 | 228 | 248 | 234 | 247 | 7.5% | 0.97 [0.93, 1.02] |
| Rerksuppaphol 2020 | 43 | 43 | 42 | 43 | 6.5% | 1.02 [0.96, 1.09] |
| Shah 2021 | 45 | 45 | 39 | 45 | 3.7% | 1.15 [1.02, 1.30] |
| Strand 2002 (3) | 207 | 223 | 195 | 224 | 6.6% | 1.07 [1.00, 1.13] |
| Strand 2002 (4) | 205 | 221 | 196 | 225 | 6.6% | 1.06 [1.00, 1.13] |
| Yazar 2016 | 49 | 55 | 39 | 55 | 1.9% | 1.26 [1.04, 1.52] |
| **Subtotal (95% CI)** | | **1279** | | **1283** | **49.2%** | **1.05 [1.01, 1.10]** |
| Total events | 1200 | | 1147 | | | |
| Heterogeneity: Tau² = 0.00; Chi² = 24.23, df = 8 (P = 0.002); I² = 67% | | | | | | |
| Test for overall effect: Z = 2.44 (P = 0.01) | | | | | | |
| **6.3.2 <6 months** | | | | | | |
| FischerWalker 2006 | 403 | 538 | 427 | 536 | 6.4% | 0.94 [0.88, 1.00] |
| **Subtotal (95% CI)** | | **538** | | **536** | **6.4%** | **0.94 [0.88, 1.00]** |
| Total events | 403 | | 427 | | | |
| Heterogeneity: Not applicable | | | | | | |
| Test for overall effect: Z = 1.86 (P = 0.06) | | | | | | |
| **6.3.3 >6 months and <6 months** | | | | | | |
| Awasthi 2006 | 773 | 998 | 724 | 984 | 7.3% | 1.05 [1.00, 1.11] |
| Bhatnagar 2004 | 131 | 132 | 125 | 134 | 7.5% | 1.06 [1.01, 1.12] |
| Crisinel 2015 | 35 | 37 | 31 | 39 | 2.2% | 1.19 [1.00, 1.42] |
| Dutta 2000 | 44 | 44 | 32 | 36 | 3.6% | 1.13 [0.99, 1.27] |
| Gregorio 2007 | 54 | 59 | 43 | 57 | 2.4% | 1.21 [1.03, 1.43] |
| Kakar 2022 | 72 | 72 | 68 | 72 | 6.6% | 1.06 [0.99, 1.13] |
| Patel 2015 | 43 | 43 | 44 | 53 | 3.5% | 1.20 [1.06, 1.36] |
| Patro 2010 | 68 | 69 | 69 | 72 | 7.0% | 1.03 [0.97, 1.09] |
| Polat 2003 (5) | 35 | 40 | 20 | 36 | 0.8% | 1.57 [1.15, 2.16] |
| Polat 2003 (6) | 44 | 52 | 34 | 54 | 1.4% | 1.34 [1.06, 1.70] |
| Shimelis 2008 | 99 | 179 | 121 | 222 | 2.2% | 1.01 [0.85, 1.21] |
| **Subtotal (95% CI)** | | **1725** | | **1759** | **44.4%** | **1.09 [1.05, 1.14]** |
| Total events | 1398 | | 1311 | | | |
| Heterogeneity: Tau² = 0.00; Chi² = 20.18, df = 10 (P = 0.03); I² = 50% | | | | | | |
| Test for overall effect: Z = 4.19 (P < 0.0001) | | | | | | |
| **Total (95% CI)** | | **3542** | | **3578** | **100.0%** | **1.07 [1.03, 1.10]** |
| Total events | 3001 | | 2885 | | | |
| Heterogeneity: Tau² = 0.00; Chi² = 56.89, df = 20 (P < 0.0001); I² = 65% | | | | | | |
| Test for overall effect: Z = 4.13 (P < 0.0001) | | | | | | |
| Test for subgroup differences: Chi² = 14.69, df = 2 (P = 0.0006), I² = 86.4% | | | | | | |

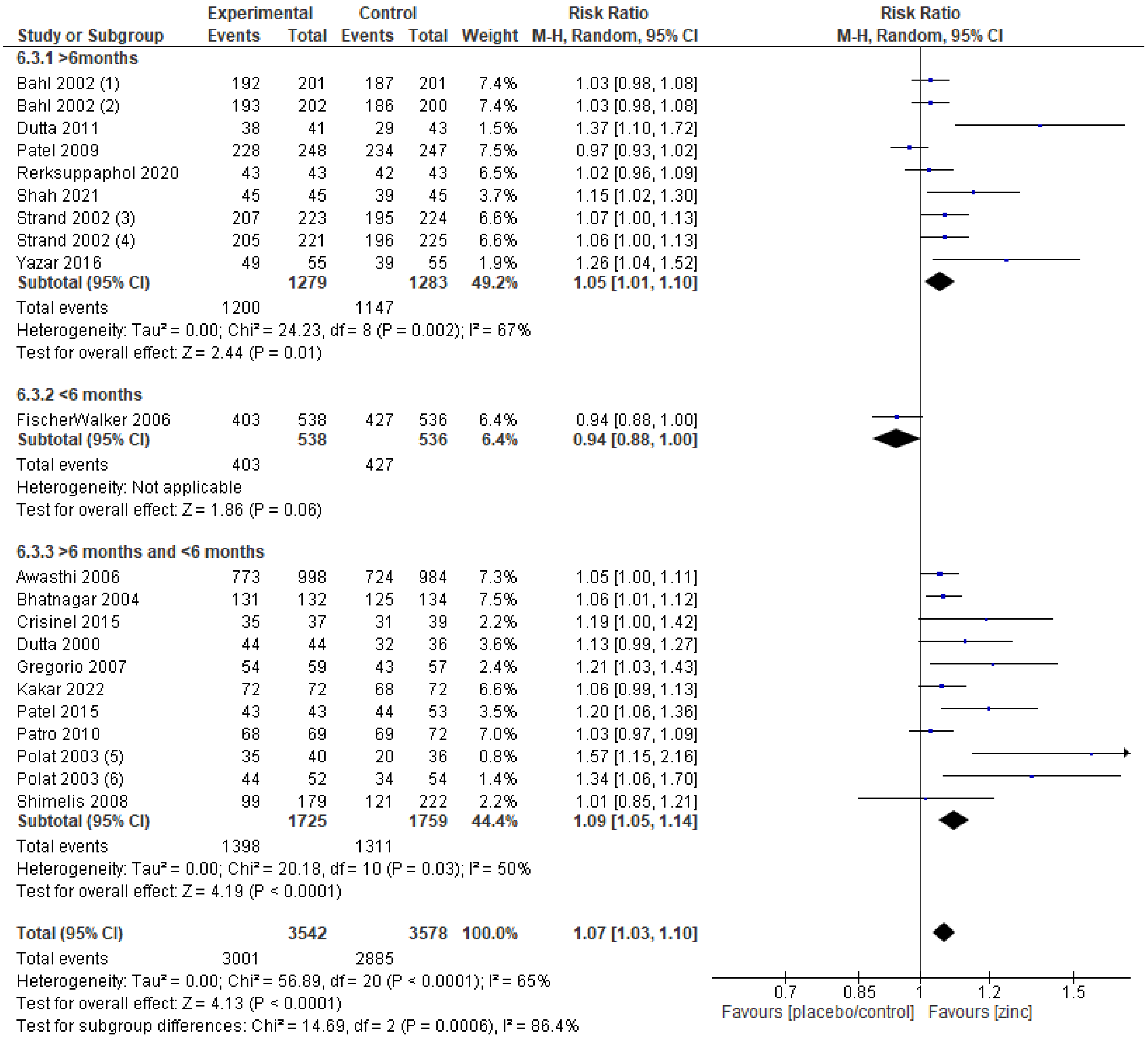


Footnotes
(1) zinc ORS
(2) zinc syrup
(3) caretakers
(4) fieldworkers
(5) low serum zinc levels
(6) normal serum zinc levels

Figure 1H

## Outcome 1: Recovery from diarrhea (based on study setting)

| Study or Subgroup | Experimental Events | Experimental Total | Control Events | Control Total | Weight | Risk Ratio M-H, Random, 95% CI |
|---|---|---|---|---|---|---|
| **7.3.1 Inpatient** | | | | | | |
| Bhatnagar 2004 | 131 | 132 | 125 | 134 | 7.5% | 1.06 [1.01, 1.12] |
| Dutta 2000 | 44 | 44 | 32 | 36 | 3.6% | 1.13 [0.99, 1.27] |
| Dutta 2011 | 38 | 41 | 29 | 43 | 1.5% | 1.37 [1.10, 1.72] |
| Rerksuppaphol 2020 | 43 | 43 | 42 | 43 | 6.5% | 1.02 [0.96, 1.09] |
| Shah 2021 | 45 | 45 | 39 | 45 | 3.7% | 1.15 [1.02, 1.30] |
| **Subtotal (95% CI)** | | **305** | | **301** | **22.7%** | **1.10 [1.02, 1.19]** |
| Total events | 301 | | 267 | | | |
| Heterogeneity: Tau² = 0.00; Chi² = 13.33, df = 4 (P = 0.010); I² = 70% | | | | | | |
| Test for overall effect: Z = 2.51 (P = 0.01) | | | | | | |
| **7.3.2 Outpatient** | | | | | | |
| Awasthi 2006 | 773 | 998 | 724 | 984 | 7.3% | 1.05 [1.00, 1.11] |
| Bahl 2002 (1) | 193 | 202 | 186 | 200 | 7.4% | 1.03 [0.98, 1.08] |
| Bahl 2002 (2) | 192 | 201 | 187 | 201 | 7.4% | 1.03 [0.98, 1.08] |
| FischerWalker 2006 | 403 | 538 | 427 | 536 | 6.4% | 0.94 [0.88, 1.00] |
| Gregorio 2007 | 54 | 59 | 43 | 57 | 2.4% | 1.21 [1.03, 1.43] |
| Patel 2015 | 43 | 43 | 44 | 53 | 3.5% | 1.20 [1.06, 1.36] |
| Polat 2003 (3) | 44 | 52 | 34 | 54 | 1.4% | 1.34 [1.06, 1.70] |
| Polat 2003 (4) | 35 | 40 | 20 | 36 | 0.8% | 1.57 [1.15, 2.16] |
| Shimelis 2008 | 99 | 179 | 121 | 222 | 2.2% | 1.01 [0.85, 1.21] |
| Strand 2002 (5) | 205 | 221 | 196 | 225 | 6.6% | 1.06 [1.00, 1.13] |
| Strand 2002 (6) | 207 | 223 | 195 | 224 | 6.6% | 1.07 [1.00, 1.13] |
| Yazar 2016 | 49 | 55 | 39 | 55 | 1.9% | 1.26 [1.04, 1.52] |
| **Subtotal (95% CI)** | | **2811** | | **2847** | **54.0%** | **1.07 [1.03, 1.12]** |
| Total events | 2297 | | 2216 | | | |
| Heterogeneity: Tau² = 0.00; Chi² = 34.09, df = 11 (P = 0.0003); I² = 68% | | | | | | |
| Test for overall effect: Z = 3.09 (P = 0.002) | | | | | | |
| **7.3.3 Inpatient and outpatient** | | | | | | |
| Crisinel 2015 | 35 | 37 | 31 | 39 | 2.2% | 1.19 [1.00, 1.42] |
| Patel 2009 | 228 | 248 | 234 | 247 | 7.5% | 0.97 [0.93, 1.02] |
| Patro 2010 | 68 | 69 | 69 | 72 | 7.0% | 1.03 [0.97, 1.09] |
| **Subtotal (95% CI)** | | **354** | | **358** | **16.7%** | **1.02 [0.95, 1.10]** |
| Total events | 331 | | 334 | | | |
| Heterogeneity: Tau² = 0.00; Chi² = 6.22, df = 2 (P = 0.04); I² = 68% | | | | | | |
| Test for overall effect: Z = 0.56 (P = 0.57) | | | | | | |
| **7.3.4 Unspecified** | | | | | | |
| Kakar 2022 | 72 | 72 | 68 | 72 | 6.6% | 1.06 [0.99, 1.13] |
| **Subtotal (95% CI)** | | **72** | | **72** | **6.6%** | **1.06 [0.99, 1.13]** |
| Total events | 72 | | 68 | | | |
| Heterogeneity: Not applicable | | | | | | |
| Test for overall effect: Z = 1.80 (P = 0.07) | | | | | | |
| **Total (95% CI)** | | **3542** | | **3578** | **100.0%** | **1.07 [1.03, 1.10]** |
| Total events | 3001 | | 2885 | | | |
| Heterogeneity: Tau² = 0.00; Chi² = 56.89, df = 20 (P < 0.0001); I² = 65% | | | | | | |
| Test for overall effect: Z = 4.13 (P < 0.0001) | | | | | | |
| Test for subgroup differences: Chi² = 2.13, df = 3 (P = 0.55), I² = 0% | | | | | | |

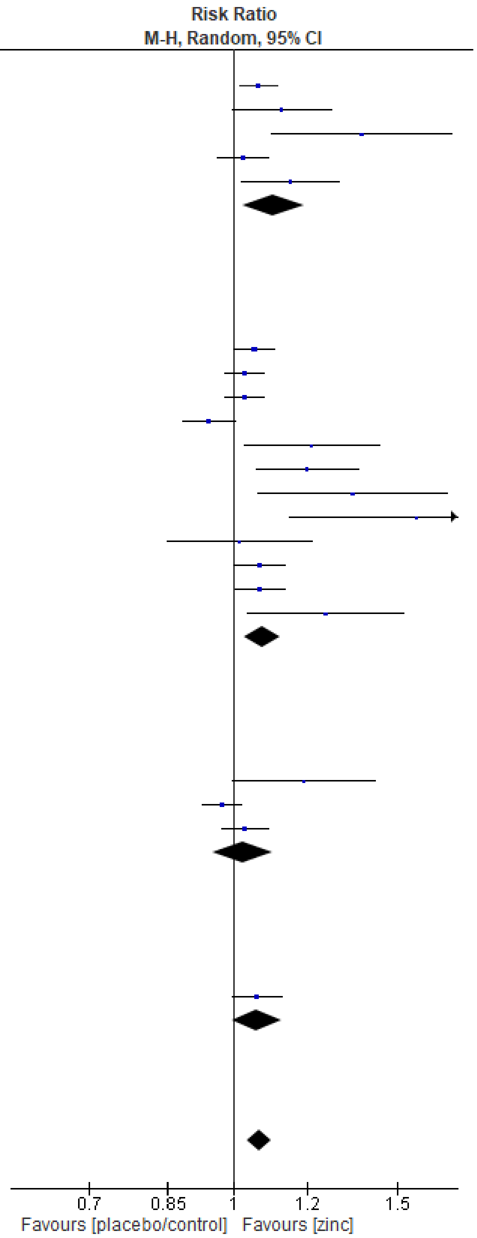


Footnotes
(1) zinc syrup
(2) zinc ORS
(3) normal serum zinc levels
(4) low serum zinc levels
(5) fieldworkers
(6) caretakers

Figure 1I

## Outcome 1: Recovery from diarrhea (by day 5 and day7)

| Study or Subgroup | Experimental Events | Experimental Total | Control Events | Control Total | Weight | Risk Ratio M-H, Random, 95% CI |
|---|---|---|---|---|---|---|
| **1.6.1 day5** | | | | | | |
| Awasthi 2006 | 773 | 998 | 724 | 984 | 11.8% | 1.05 [1.00, 1.11] |
| Bahl 2002 (1) | 180 | 201 | 175 | 200 | 9.9% | 1.02 [0.95, 1.10] |
| Bahl 2002 (2) | 185 | 202 | 176 | 201 | 10.2% | 1.05 [0.98, 1.12] |
| Bhatnagar 2004 | 115 | 132 | 107 | 134 | 6.9% | 1.09 [0.98, 1.21] |
| Crisinel 2015 | 35 | 37 | 31 | 39 | 3.6% | 1.19 [1.00, 1.42] |
| Dutta 2000 | 44 | 44 | 32 | 36 | 5.9% | 1.13 [0.99, 1.27] |
| Dutta 2011 | 38 | 41 | 29 | 43 | 2.5% | 1.37 [1.10, 1.72] |
| Kakar 2022 | 72 | 72 | 68 | 72 | 10.7% | 1.06 [0.99, 1.13] |
| Patel 2009 | 229 | 247 | 236 | 248 | 12.3% | 0.97 [0.93, 1.02] |
| Patel 2015 | 43 | 43 | 44 | 53 | 5.7% | 1.20 [1.06, 1.36] |
| Rerksuppaphol 2020 | 43 | 43 | 40 | 43 | 8.0% | 1.07 [0.98, 1.18] |
| Shah 2021 | 45 | 45 | 39 | 45 | 6.0% | 1.15 [1.02, 1.30] |
| Shimelis 2008 | 99 | 179 | 121 | 222 | 3.6% | 1.01 [0.85, 1.21] |
| Yazar 2016 | 49 | 55 | 39 | 55 | 3.1% | 1.26 [1.04, 1.52] |
| **Subtotal (95% CI)** | | **2339** | | **2375** | **100.0%** | **1.08 [1.04, 1.12]** |
| Total events | 1950 | | 1861 | | | |
| Heterogeneity: Tau² = 0.00; Chi² = 31.19, df = 13 (P = 0.003); I² = 58% | | | | | | |
| Test for overall effect: Z = 3.81 (P = 0.0001) | | | | | | |
| **1.6.2 day7** | | | | | | |
| Bahl 2002 (3) | 192 | 201 | 187 | 201 | 11.7% | 1.03 [0.98, 1.08] |
| Bahl 2002 (4) | 193 | 202 | 186 | 200 | 11.7% | 1.03 [0.98, 1.08] |
| Bhatnagar 2004 | 131 | 132 | 125 | 134 | 11.7% | 1.06 [1.01, 1.12] |
| FischerWalker 2006 | 403 | 538 | 427 | 536 | 9.7% | 0.94 [0.88, 1.00] |
| Patel 2009 | 228 | 248 | 234 | 247 | 11.8% | 0.97 [0.93, 1.02] |
| Patro 2010 | 68 | 69 | 69 | 72 | 10.7% | 1.03 [0.97, 1.09] |
| Polat 2003 (5) | 44 | 52 | 34 | 54 | 1.8% | 1.34 [1.06, 1.70] |
| Polat 2003 (6) | 35 | 40 | 20 | 36 | 1.0% | 1.57 [1.15, 2.16] |
| Rerksuppaphol 2020 | 43 | 43 | 42 | 43 | 9.8% | 1.02 [0.96, 1.09] |
| Strand 2002 (7) | 207 | 223 | 195 | 224 | 10.0% | 1.07 [1.00, 1.13] |
| Strand 2002 (8) | 205 | 221 | 196 | 225 | 10.0% | 1.06 [1.00, 1.13] |
| **Subtotal (95% CI)** | | **1969** | | **1972** | **100.0%** | **1.03 [1.00, 1.07]** |
| Total events | 1749 | | 1715 | | | |
| Heterogeneity: Tau² = 0.00; Chi² = 29.71, df = 10 (P = 0.0010); I² = 66% | | | | | | |
| Test for overall effect: Z = 1.88 (P = 0.06) | | | | | | |

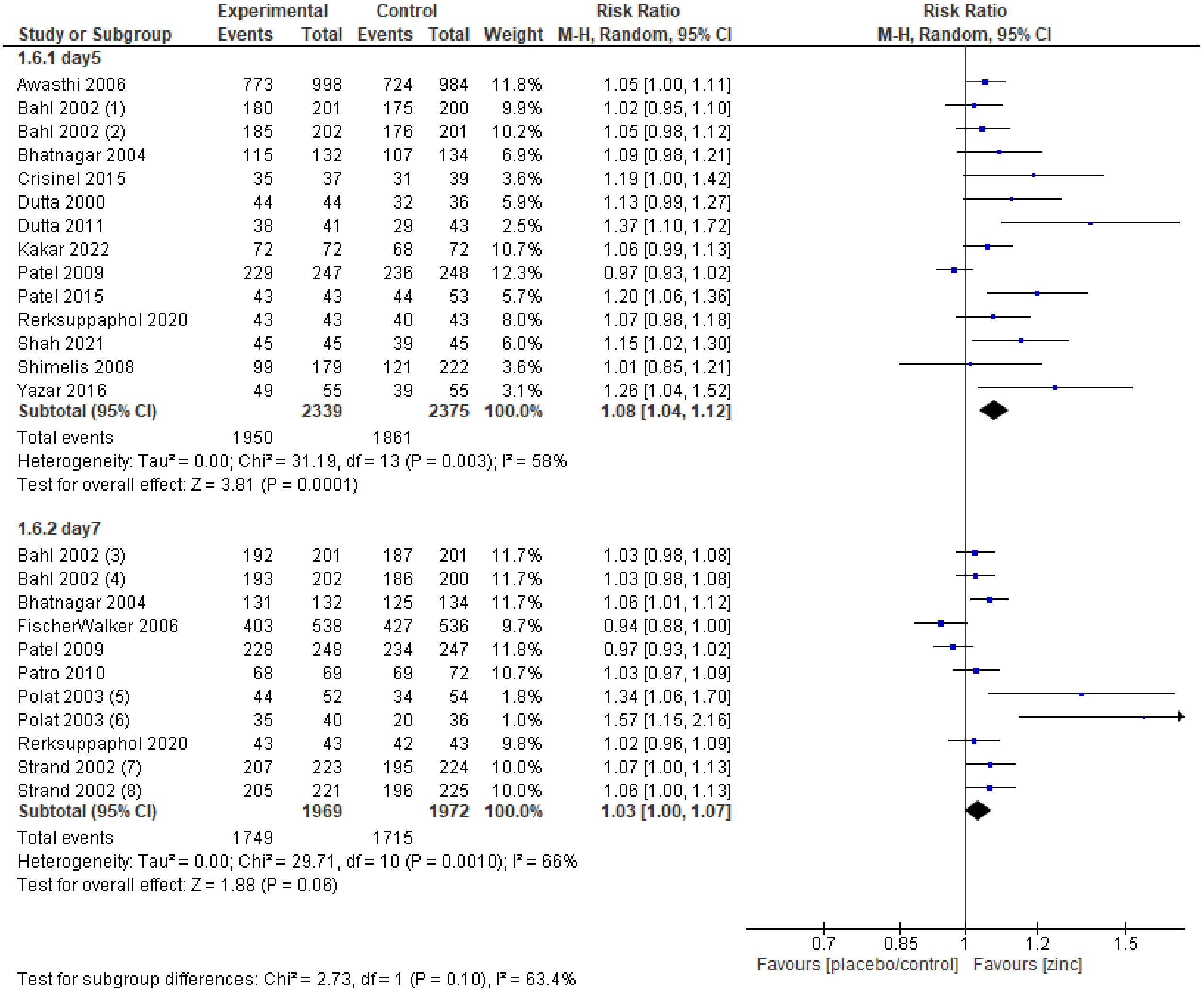


Test for subgroup differences: Chi² = 2.73, df = 1 (P = 0.10), I² = 63.4%

Footnotes
(1) ORS
(2) syrup
(3) zinc ORS
(4) zinc syrup
(5) normal serum zinc levels
(6) low serum zinc levels
(7) caretakers
(8) fieldworkers

Figure 1J

Outcome 1: Recovery from diarrhea- sensitivity analysis

| Study or Subgroup | Experimental Events | Experimental Total | Control Events | Control Total | Weight | Risk Ratio M-H, Random, 95% CI |
|---|---|---|---|---|---|---|
| Bahl 2002 (1) | 192 | 201 | 187 | 201 | 11.0% | 1.03 [0.98, 1.08] |
| Bahl 2002 (2) | 193 | 202 | 186 | 200 | 11.0% | 1.03 [0.98, 1.08] |
| Bhatnagar 2004 | 131 | 132 | 125 | 134 | 11.1% | 1.06 [1.01, 1.12] |
| Crisinel 2015 | 35 | 37 | 31 | 39 | 3.6% | 1.19 [1.00, 1.42] |
| Dutta 2000 | 44 | 44 | 32 | 36 | 5.7% | 1.13 [0.99, 1.27] |
| Dutta 2011 | 38 | 41 | 29 | 43 | 2.5% | 1.37 [1.10, 1.72] |
| FischerWalker 2006 | 403 | 538 | 427 | 536 | 9.7% | 0.94 [0.88, 1.00] |
| Patel 2015 | 43 | 43 | 44 | 53 | 5.5% | 1.20 [1.06, 1.36] |
| Patro 2010 | 68 | 69 | 69 | 72 | 10.4% | 1.03 [0.97, 1.09] |
| Polat 2003 (3) | 35 | 40 | 20 | 36 | 1.4% | 1.57 [1.15, 2.16] |
| Polat 2003 (4) | 44 | 52 | 34 | 54 | 2.3% | 1.34 [1.06, 1.70] |
| Rerksuppaphol 2020 | 43 | 43 | 42 | 43 | 9.8% | 1.02 [0.96, 1.09] |
| Shah 2021 | 45 | 45 | 39 | 45 | 5.9% | 1.15 [1.02, 1.30] |
| Strand 2002 (5) | 205 | 221 | 196 | 225 | 9.9% | 1.06 [1.00, 1.13] |
| **Total (95% CI)** | | **1708** | | **1717** | **100.0%** | **1.07 [1.03, 1.12]** |
| Total events | 1519 | | 1461 | | | |

Heterogeneity: Tau² = 0.00; Chi² = 39.84, df = 13 (P = 0.0001); I² = 67%
Test for overall effect: Z = 3.53 (P = 0.0004)

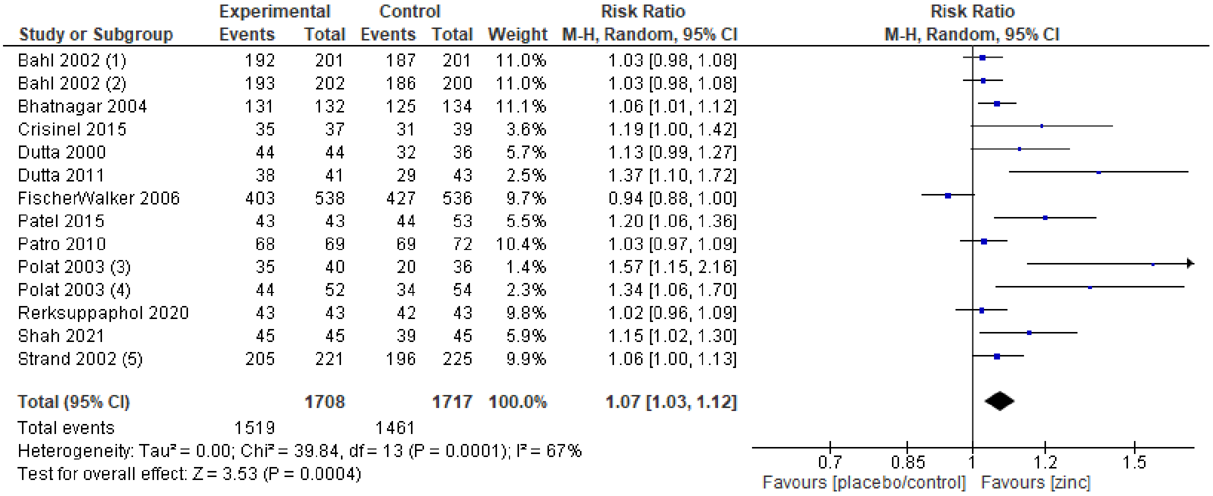


Footnotes
(1) zinc ORS
(2) zinc syrup
(3) low serum zinc levels
(4) normal serum zinc levels
(5) fieldworkers

**Vomiting**

Figure 2A

Outcome 2- Vomiting (subgroups based on definition of diarrhea)

| Study or Subgroup | Experimental Events | Experimental Total | Control Events | Control Total | Weight | Risk Ratio M-H, Random, 95% CI |
|---|---|---|---|---|---|---|
| **13.16.1 WHO definition** | | | | | | |
| Awasthi 2006 | 86 | 998 | 67 | 984 | 9.1% | 1.27 [0.93, 1.72] |
| Bhatnagar 2004 | 86 | 132 | 79 | 134 | 10.7% | 1.11 [0.92, 1.33] |
| Brooks 2005 | 8 | 43 | 3 | 44 | 1.8% | 2.73 [0.78, 9.61] |
| Brooks 2005 | 6 | 43 | 4 | 45 | 2.0% | 1.57 [0.48, 5.18] |
| Crisinel 2015 | 30 | 42 | 26 | 45 | 9.0% | 1.24 [0.90, 1.69] |
| FischerWalker 2006 | 47 | 538 | 33 | 536 | 7.3% | 1.42 [0.92, 2.18] |
| Strand 2002 (1) | 73 | 221 | 42 | 224 | 8.7% | 1.76 [1.27, 2.45] |
| Strand 2002 (2) | 87 | 224 | 43 | 225 | 8.9% | 2.03 [1.48, 2.78] |
| Wadhwa 2011 | 112 | 248 | 104 | 252 | 10.6% | 1.09 [0.89, 1.34] |
| **Subtotal (95% CI)** | | **2489** | | **2489** | **68.2%** | **1.37 [1.15, 1.63]** |
| Total events | 535 | | 401 | | | |
| Heterogeneity: Tau² = 0.04; Chi² = 19.23, df = 8 (P = 0.01); I² = 58% | | | | | | |
| Test for overall effect: Z = 3.51 (P = 0.0004) | | | | | | |
| **13.16.2 Other definition** | | | | | | |
| Bahl 2002 (3) | 21 | 109 | 28 | 105 | 6.4% | 0.72 [0.44, 1.19] |
| Bahl 2002 (4) | 37 | 97 | 27 | 104 | 7.6% | 1.47 [0.97, 2.22] |
| Polat 2003 (5) | 3 | 52 | 1 | 54 | 0.6% | 3.12 [0.33, 29.00] |
| Polat 2003 (6) | 2 | 40 | 1 | 36 | 0.6% | 1.80 [0.17, 19.02] |
| Shimelis 2008 | 46 | 179 | 24 | 222 | 7.0% | 2.38 [1.51, 3.74] |
| **Subtotal (95% CI)** | | **477** | | **521** | **22.2%** | **1.46 [0.83, 2.55]** |
| Total events | 109 | | 81 | | | |
| Heterogeneity: Tau² = 0.22; Chi² = 12.60, df = 4 (P = 0.01); I² = 68% | | | | | | |
| Test for overall effect: Z = 1.31 (P = 0.19) | | | | | | |
| **13.16.3 Not mentioned** | | | | | | |
| Larson 2005 | 139 | 534 | 64 | 533 | 9.6% | 2.17 [1.65, 2.84] |
| **Subtotal (95% CI)** | | **534** | | **533** | **9.6%** | **2.17 [1.65, 2.84]** |
| Total events | 139 | | 64 | | | |
| Heterogeneity: Not applicable | | | | | | |
| Test for overall effect: Z = 5.60 (P < 0.00001) | | | | | | |
| **Total (95% CI)** | | **3500** | | **3543** | **100.0%** | **1.46 [1.22, 1.76]** |
| Total events | 783 | | 546 | | | |
| Heterogeneity: Tau² = 0.07; Chi² = 45.55, df = 14 (P < 0.0001); I² = 69% | | | | | | |
| Test for overall effect: Z = 4.04 (P < 0.0001) | | | | | | |
| Test for subgroup differences: Chi² = 7.83, df = 2 (P = 0.02), I² = 74.5% | | | | | | |

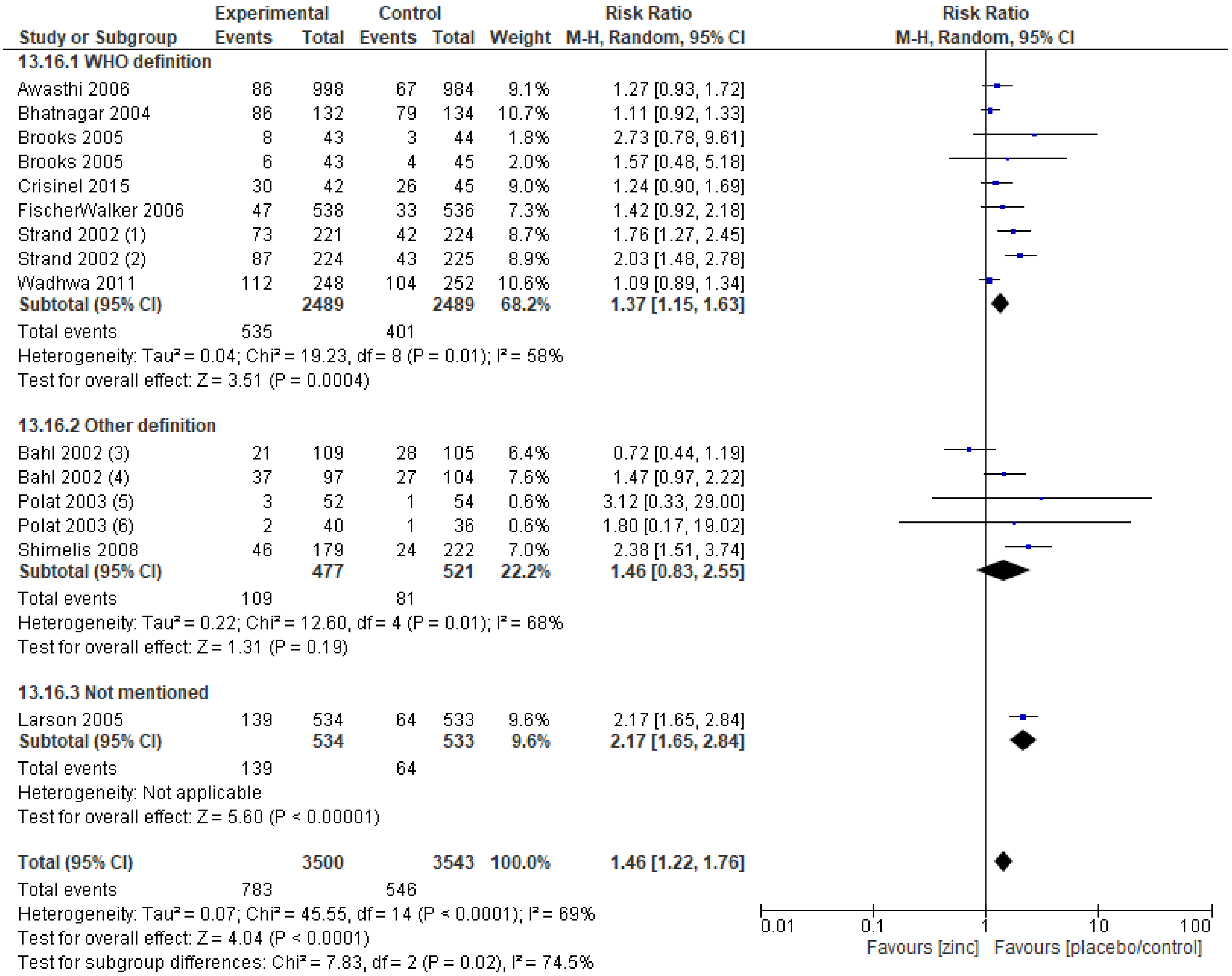


Footnotes
(1) zinc-field workers
(2) zinc-caretakers
(3) zinc-ORS
(4) zinc syrup
(5) normal serum zinc at enrollment
(6) low zinc at enrollment

Figure 2B

## Outcome 2: Vomiting (subgroups based on dose of zinc)

| Study or Subgroup | Experimental Events | Experimental Total | Control Events | Control Total | Weight | Risk Ratio M-H, Random, 95% CI |
|---|---|---|---|---|---|---|
| **3.10.1 5mg** | | | | | | |
| Brooks 2005 | 8 | 43 | 3 | 44 | 1.8% | 2.73 [0.78, 9.61] |
| **Subtotal (95% CI)** | | **43** | | **44** | **1.8%** | **2.73 [0.78, 9.61]** |
| Total events | 8 | | 3 | | | |
| Heterogeneity: Not applicable | | | | | | |
| Test for overall effect: Z = 1.56 (P = 0.12) | | | | | | |
| **3.10.2 10mg** | | | | | | |
| FischerWalker 2006 | 47 | 538 | 33 | 536 | 7.3% | 1.42 [0.92, 2.18] |
| **Subtotal (95% CI)** | | **538** | | **536** | **7.3%** | **1.42 [0.92, 2.18]** |
| Total events | 47 | | 33 | | | |
| Heterogeneity: Not applicable | | | | | | |
| Test for overall effect: Z = 1.60 (P = 0.11) | | | | | | |
| **3.10.3 20mg** | | | | | | |
| Brooks 2005 | 6 | 43 | 4 | 45 | 2.0% | 1.57 [0.48, 5.18] |
| Larson 2005 | 139 | 534 | 64 | 533 | 9.6% | 2.17 [1.65, 2.84] |
| Polat 2003 (1) | 2 | 40 | 1 | 36 | 0.6% | 1.80 [0.17, 19.02] |
| Polat 2003 (2) | 3 | 52 | 1 | 54 | 0.6% | 3.12 [0.33, 29.00] |
| Shimelis 2008 | 46 | 179 | 24 | 222 | 7.0% | 2.38 [1.51, 3.74] |
| **Subtotal (95% CI)** | | **848** | | **890** | **19.8%** | **2.20 [1.75, 2.75]** |
| Total events | 196 | | 94 | | | |
| Heterogeneity: Tau² = 0.00; Chi² = 0.55, df = 4 (P = 0.97); I² = 0% | | | | | | |
| Test for overall effect: Z = 6.83 (P < 0.00001) | | | | | | |
| **3.10.4 dose by weight or age** | | | | | | |
| Bahl 2002 (3) | 37 | 97 | 27 | 104 | 7.6% | 1.47 [0.97, 2.22] |
| Bahl 2002 (4) | 21 | 109 | 28 | 105 | 6.4% | 0.72 [0.44, 1.19] |
| Bhatnagar 2004 | 86 | 132 | 79 | 134 | 10.7% | 1.11 [0.92, 1.33] |
| Crisinel 2015 | 30 | 42 | 26 | 45 | 9.0% | 1.24 [0.90, 1.69] |
| Strand 2002 (5) | 73 | 221 | 42 | 224 | 8.7% | 1.76 [1.27, 2.45] |
| Strand 2002 (6) | 87 | 224 | 43 | 225 | 8.9% | 2.03 [1.48, 2.78] |
| Wadhwa 2011 | 112 | 248 | 104 | 252 | 10.6% | 1.09 [0.89, 1.34] |
| **Subtotal (95% CI)** | | **1073** | | **1089** | **61.9%** | **1.30 [1.04, 1.61]** |
| Total events | 446 | | 349 | | | |
| Heterogeneity: Tau² = 0.06; Chi² = 22.44, df = 6 (P = 0.001); I² = 73% | | | | | | |
| Test for overall effect: Z = 2.35 (P = 0.02) | | | | | | |
| **3.10.5 unspecified** | | | | | | |
| Awasthi 2006 (7) | 86 | 998 | 67 | 984 | 9.1% | 1.27 [0.93, 1.72] |
| **Subtotal (95% CI)** | | **998** | | **984** | **9.1%** | **1.27 [0.93, 1.72]** |
| Total events | 86 | | 67 | | | |
| Heterogeneity: Not applicable | | | | | | |
| Test for overall effect: Z = 1.50 (P = 0.13) | | | | | | |
| **Total (95% CI)** | | **3500** | | **3543** | **100.0%** | **1.46 [1.22, 1.76]** |
| Total events | 783 | | 546 | | | |
| Heterogeneity: Tau² = 0.07; Chi² = 45.55, df = 14 (P < 0.0001); I² = 69% | | | | | | |
| Test for overall effect: Z = 4.04 (P < 0.0001) | | | | | | |
| Test for subgroup differences: Chi² = 14.40, df = 4 (P = 0.006), I² = 72.2% | | | | | | |

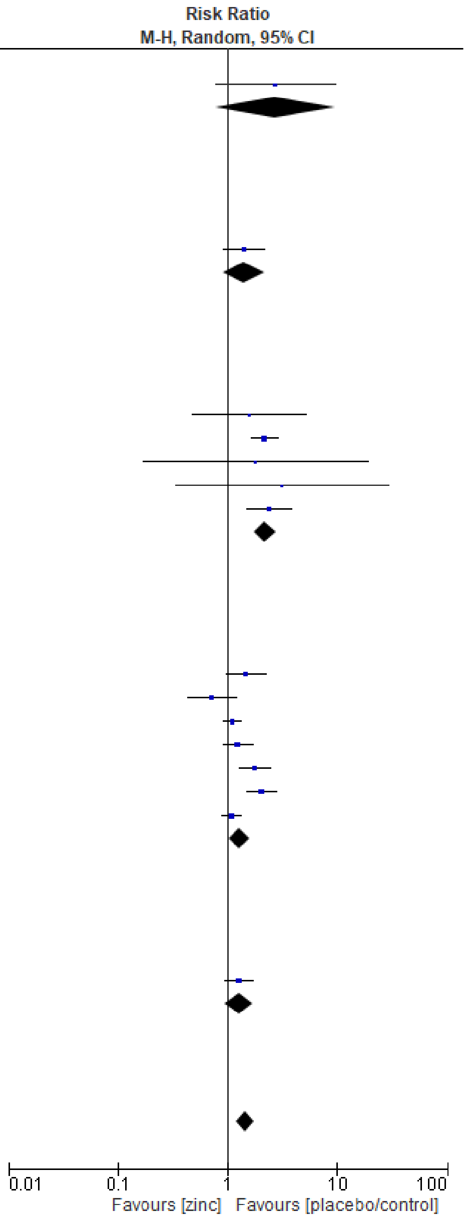


Footnotes
(1) low zinc at enrollment
(2) normal serum zinc at enrollment
(3) zinc syrup
(4) zinc-ORS
(5) zinc-field workers
(6) zinc-caretakers
(7) Ethiopia and Lucknow, India gave two zinctablets, whereas the other sites gave one zinc tablet once perday

Figure 2C

## Outcome 2: Vomiting (subgroups based on duration of zinc supplementation)

| Study or Subgroup | Experimental Events | Experimental Total | Control Events | Control Total | Weight | Risk Ratio M-H, Random, 95% CI |
|---|---|---|---|---|---|---|
| **8.4.1 10d** | | | | | | |
| Crisinel 2015 | 30 | 42 | 26 | 45 | 9.0% | 1.24 [0.90, 1.69] |
| **Subtotal (95% CI)** | | **42** | | **45** | **9.0%** | **1.24 [0.90, 1.69]** |
| Total events | 30 | | 26 | | | |
| Heterogeneity: Not applicable | | | | | | |
| Test for overall effect: Z = 1.32 (P = 0.19) | | | | | | |
| **8.4.2 14d** | | | | | | |
| Awasthi 2006 | 86 | 998 | 67 | 984 | 9.1% | 1.27 [0.93, 1.72] |
| Bahl 2002 (1) | 37 | 97 | 27 | 104 | 7.6% | 1.47 [0.97, 2.22] |
| Bahl 2002 (2) | 21 | 109 | 28 | 105 | 6.4% | 0.72 [0.44, 1.19] |
| Bhatnagar 2004 | 86 | 132 | 79 | 134 | 10.7% | 1.11 [0.92, 1.33] |
| FischerWalker 2006 | 47 | 538 | 33 | 536 | 7.3% | 1.42 [0.92, 2.18] |
| Polat 2003 (3) | 2 | 40 | 1 | 36 | 0.6% | 1.80 [0.17, 19.02] |
| Polat 2003 (4) | 3 | 52 | 1 | 54 | 0.6% | 3.12 [0.33, 29.00] |
| Shimelis 2008 | 46 | 179 | 24 | 222 | 7.0% | 2.38 [1.51, 3.74] |
| **Subtotal (95% CI)** | | **2145** | | **2175** | **49.4%** | **1.32 [1.03, 1.67]** |
| Total events | 328 | | 260 | | | |
| Heterogeneity: Tau² = 0.06; Chi² = 16.19, df = 7 (P = 0.02); I² = 57% | | | | | | |
| Test for overall effect: Z = 2.22 (P = 0.03) | | | | | | |
| **8.4.3 unspecified** | | | | | | |
| Brooks 2005 | 8 | 43 | 3 | 44 | 1.8% | 2.73 [0.78, 9.61] |
| Brooks 2005 | 6 | 43 | 4 | 44 | 2.0% | 1.53 [0.47, 5.06] |
| Larson 2005 | 139 | 534 | 64 | 533 | 9.6% | 2.17 [1.65, 2.84] |
| Strand 2002 (5) | 73 | 221 | 42 | 224 | 8.7% | 1.76 [1.27, 2.45] |
| Strand 2002 (6) | 87 | 224 | 43 | 225 | 8.9% | 2.03 [1.48, 2.78] |
| Wadhwa 2011 | 112 | 248 | 104 | 252 | 10.6% | 1.09 [0.89, 1.34] |
| **Subtotal (95% CI)** | | **1313** | | **1322** | **41.7%** | **1.72 [1.24, 2.40]** |
| Total events | 425 | | 260 | | | |
| Heterogeneity: Tau² = 0.11; Chi² = 22.34, df = 5 (P = 0.0005); I² = 78% | | | | | | |
| Test for overall effect: Z = 3.24 (P = 0.001) | | | | | | |
| **Total (95% CI)** | | **3500** | | **3542** | **100.0%** | **1.46 [1.22, 1.76]** |
| Total events | 783 | | 546 | | | |
| Heterogeneity: Tau² = 0.07; Chi² = 45.53, df = 14 (P < 0.0001); I² = 69% | | | | | | |
| Test for overall effect: Z = 4.03 (P < 0.0001) | | | | | | |
| Test for subgroup differences: Chi² = 2.37, df = 2 (P = 0.31), I² = 15.6% | | | | | | |

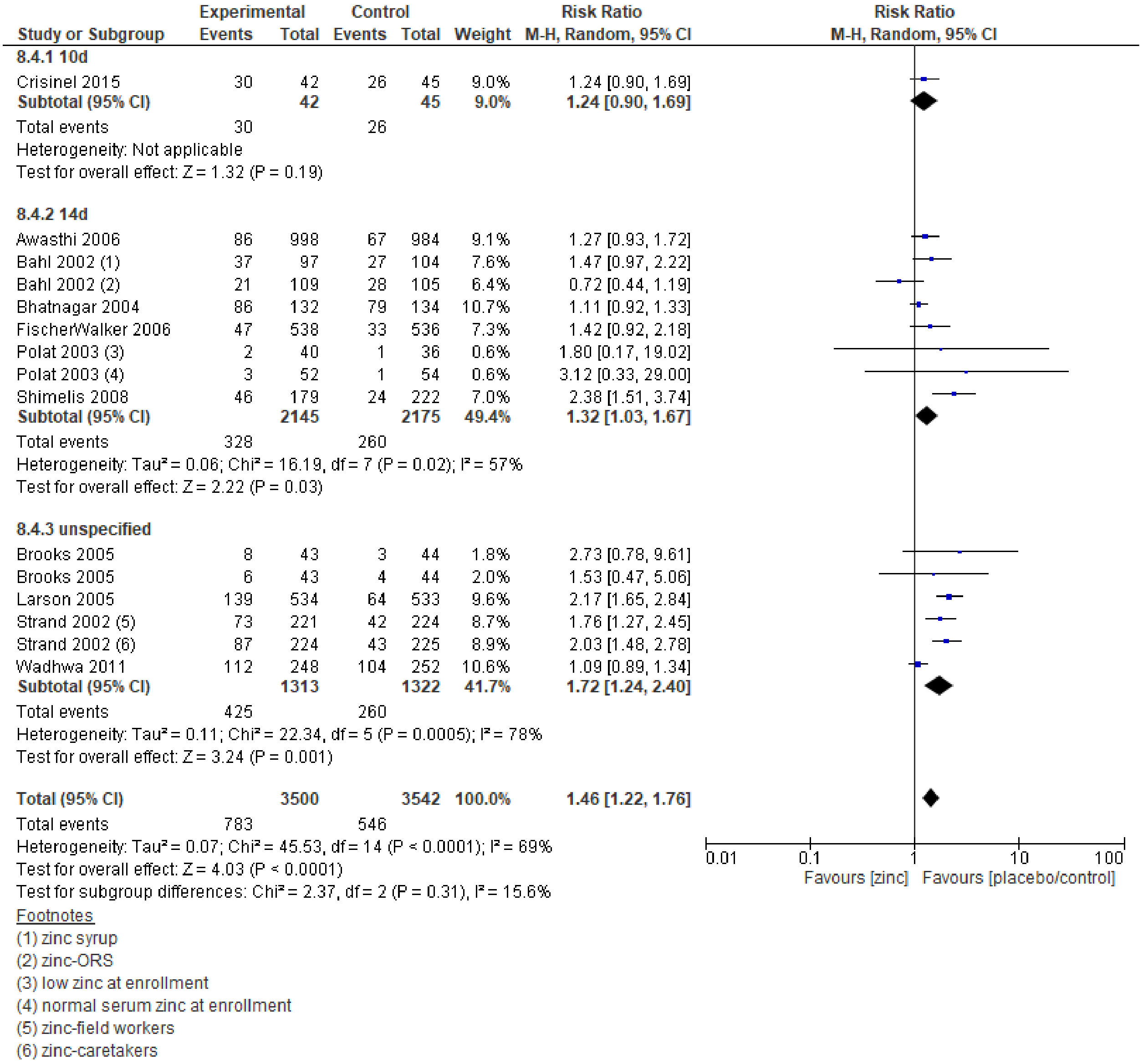


Footnotes
(1) zinc syrup
(2) zinc-ORS
(3) low zinc at enrollment
(4) normal serum zinc at enrollment
(5) zinc-field workers
(6) zinc-caretakers

Figure 2D

## Outcome 2: Vomiting (subgroups based on formulation of zinc)

| Study or Subgroup | Experimental Events | Experimental Total | Control Events | Control Total | Weight | Risk Ratio M-H, Random, 95% CI |
|---|---|---|---|---|---|---|
| **4.10.1 Zinc gluconate** | | | | | | |
| Bahl 2002 (1) | 21 | 109 | 28 | 105 | 6.4% | 0.72 [0.44, 1.19] |
| Bahl 2002 (2) | 37 | 97 | 27 | 104 | 7.6% | 1.47 [0.97, 2.22] |
| Wadhwa 2011 | 112 | 248 | 104 | 252 | 10.6% | 1.09 [0.89, 1.34] |
| **Subtotal (95% CI)** | | **454** | | **461** | **24.6%** | **1.08 [0.79, 1.48]** |
| Total events | 170 | | 159 | | | |
| Heterogeneity: Tau² = 0.04; Chi² = 4.64, df = 2 (P = 0.10); I² = 57% | | | | | | |
| Test for overall effect: Z = 0.48 (P = 0.63) | | | | | | |
| **4.10.2 Zinc sulfate** | | | | | | |
| Bhatnagar 2004 | 86 | 132 | 79 | 134 | 10.7% | 1.11 [0.92, 1.33] |
| Crisinel 2015 | 30 | 42 | 26 | 45 | 9.0% | 1.24 [0.90, 1.69] |
| Larson 2005 | 139 | 534 | 64 | 533 | 9.6% | 2.17 [1.65, 2.84] |
| Polat 2003 (3) | 3 | 52 | 1 | 54 | 0.6% | 3.12 [0.33, 29.00] |
| Polat 2003 (4) | 2 | 40 | 1 | 36 | 0.6% | 1.80 [0.17, 19.02] |
| **Subtotal (95% CI)** | | **800** | | **802** | **30.5%** | **1.48 [0.96, 2.27]** |
| Total events | 260 | | 171 | | | |
| Heterogeneity: Tau² = 0.13; Chi² = 20.37, df = 4 (P = 0.0004); I² = 80% | | | | | | |
| Test for overall effect: Z = 1.79 (P = 0.07) | | | | | | |
| **4.10.3 Zinc acetate** | | | | | | |
| Brooks 2005 (5) | 6 | 43 | 4 | 45 | 2.0% | 1.57 [0.48, 5.18] |
| Brooks 2005 (6) | 8 | 43 | 3 | 44 | 1.8% | 2.73 [0.78, 9.61] |
| **Subtotal (95% CI)** | | **86** | | **89** | **3.8%** | **2.04 [0.86, 4.85]** |
| Total events | 14 | | 7 | | | |
| Heterogeneity: Tau² = 0.00; Chi² = 0.39, df = 1 (P = 0.53); I² = 0% | | | | | | |
| Test for overall effect: Z = 1.61 (P = 0.11) | | | | | | |
| **4.10.4 unspecified** | | | | | | |
| Awasthi 2006 | 86 | 998 | 67 | 984 | 9.1% | 1.27 [0.93, 1.72] |
| FischerWalker 2006 | 47 | 538 | 33 | 536 | 7.3% | 1.42 [0.92, 2.18] |
| Shimelis 2008 | 46 | 179 | 24 | 222 | 7.0% | 2.38 [1.51, 3.74] |
| Strand 2002 (7) | 87 | 224 | 43 | 225 | 8.9% | 2.03 [1.48, 2.78] |
| Strand 2002 (8) | 73 | 221 | 42 | 224 | 8.7% | 1.76 [1.27, 2.45] |
| **Subtotal (95% CI)** | | **2160** | | **2191** | **41.1%** | **1.70 [1.36, 2.12]** |
| Total events | 339 | | 209 | | | |
| Heterogeneity: Tau² = 0.03; Chi² = 7.63, df = 4 (P = 0.11); I² = 48% | | | | | | |
| Test for overall effect: Z = 4.70 (P < 0.00001) | | | | | | |
| **Total (95% CI)** | | **3500** | | **3543** | **100.0%** | **1.46 [1.22, 1.76]** |
| Total events | 783 | | 546 | | | |
| Heterogeneity: Tau² = 0.07; Chi² = 45.55, df = 14 (P < 0.0001); I² = 69% | | | | | | |
| Test for overall effect: Z = 4.04 (P < 0.0001) | | | | | | |
| Test for subgroup differences: Chi² = 5.88, df = 3 (P = 0.12), I² = 49.0% | | | | | | |

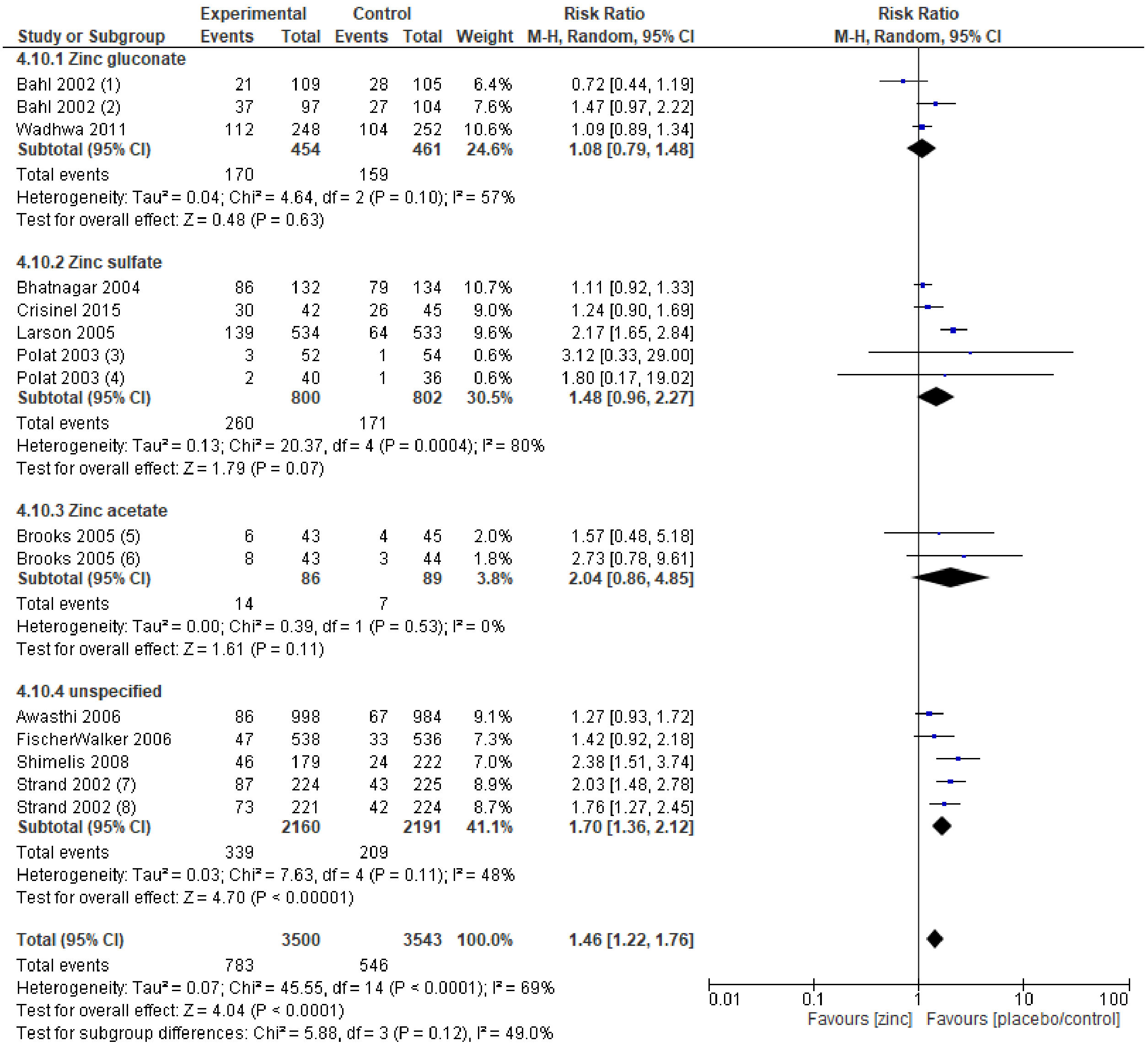


Footnotes
(1) zinc-ORS
(2) zinc syrup
(3) normal serum zinc at enrollment
(4) low zinc at enrollment
(5) 20mg
(6) 5mg
(7) zinc-caretakers
(8) zinc-field workers

Figure 2E

Outcome2: Vomiting (based on World Bank Income Classification)

| Study or Subgroup | Experimental Events | Experimental Total | Control Events | Control Total | Weight | Risk Ratio M-H, Random, 95% CI |
|---|---|---|---|---|---|---|
| **2.10.1 LMIC** | | | | | | |
| Bahl 2002 (1) | 37 | 97 | 27 | 104 | 7.6% | 1.47 [0.97, 2.22] |
| Bahl 2002 (2) | 21 | 109 | 28 | 105 | 6.4% | 0.72 [0.44, 1.19] |
| Bhatnagar 2004 | 86 | 132 | 79 | 134 | 10.7% | 1.11 [0.92, 1.33] |
| Brooks 2005 | 8 | 43 | 3 | 44 | 1.8% | 2.73 [0.78, 9.61] |
| Brooks 2005 | 6 | 43 | 4 | 44 | 2.0% | 1.53 [0.47, 5.06] |
| FischerWalker 2006 | 47 | 538 | 33 | 536 | 7.3% | 1.42 [0.92, 2.18] |
| Larson 2005 | 139 | 534 | 64 | 533 | 9.6% | 2.17 [1.65, 2.84] |
| Polat 2003 (3) | 2 | 40 | 1 | 36 | 0.6% | 1.80 [0.17, 19.02] |
| Polat 2003 (4) | 3 | 52 | 1 | 54 | 0.6% | 3.12 [0.33, 29.00] |
| Shimelis 2008 | 46 | 179 | 24 | 222 | 7.0% | 2.38 [1.51, 3.74] |
| Strand 2002 (5) | 73 | 221 | 42 | 224 | 8.7% | 1.76 [1.27, 2.45] |
| Strand 2002 (6) | 87 | 224 | 43 | 225 | 8.9% | 2.03 [1.48, 2.78] |
| Wadhwa 2011 | 112 | 248 | 104 | 252 | 10.6% | 1.09 [0.89, 1.34] |
| **Subtotal (95% CI)** | | **2460** | | **2513** | **82.0%** | **1.52 [1.21, 1.90]** |
| Total events | 667 | | 453 | | | |
| Heterogeneity: Tau² = 0.10; Chi² = 44.94, df = 12 (P < 0.0001); I² = 73% | | | | | | |
| Test for overall effect: Z = 3.67 (P = 0.0002) | | | | | | |
| **2.10.3 HIC** | | | | | | |
| Crisinel 2015 | 30 | 42 | 26 | 45 | 9.0% | 1.24 [0.90, 1.69] |
| **Subtotal (95% CI)** | | **42** | | **45** | **9.0%** | **1.24 [0.90, 1.69]** |
| Total events | 30 | | 26 | | | |
| Heterogeneity: Not applicable | | | | | | |
| Test for overall effect: Z = 1.32 (P = 0.19) | | | | | | |
| **2.10.4 mixed** | | | | | | |
| Awasthi 2006 | 86 | 998 | 67 | 984 | 9.1% | 1.27 [0.93, 1.72] |
| **Subtotal (95% CI)** | | **998** | | **984** | **9.1%** | **1.27 [0.93, 1.72]** |
| Total events | 86 | | 67 | | | |
| Heterogeneity: Not applicable | | | | | | |
| Test for overall effect: Z = 1.50 (P = 0.13) | | | | | | |
| **Total (95% CI)** | | **3500** | | **3542** | **100.0%** | **1.46 [1.22, 1.76]** |
| Total events | 783 | | 546 | | | |
| Heterogeneity: Tau² = 0.07; Chi² = 45.53, df = 14 (P < 0.0001); I² = 69% | | | | | | |
| Test for overall effect: Z = 4.03 (P < 0.0001) | | | | | | |
| Test for subgroup differences: Chi² = 1.49, df = 2 (P = 0.48), I² = 0% | | | | | | |

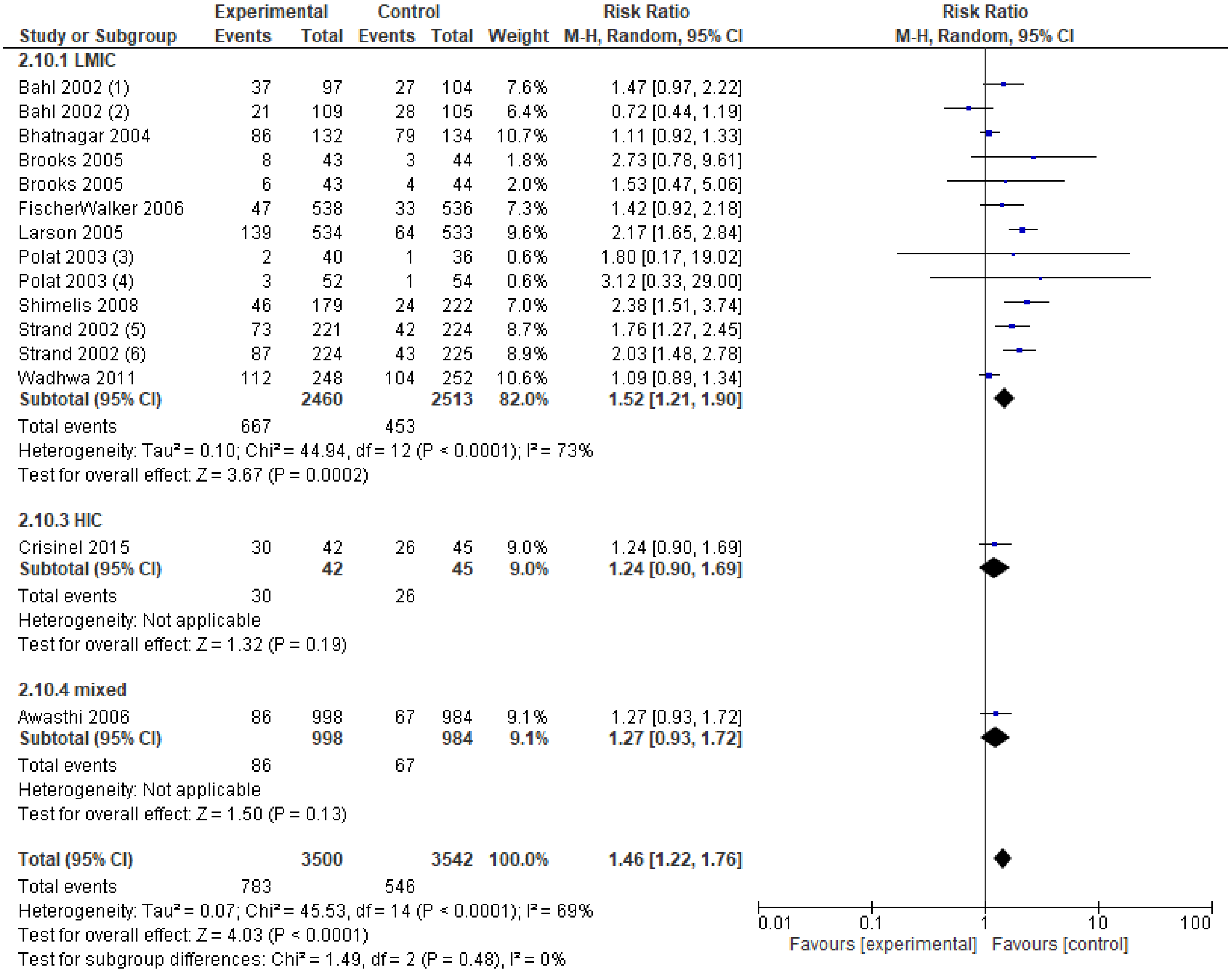


Footnotes
(1) zinc syrup
(2) zinc-ORS
(3) low zinc at enrollment
(4) normal serum zinc at enrollment
(5) zinc-field workers
(6) zinc-caretakers

Figure 2F

Outcome 2: Vomiting (based on form of zinc given)

| Study or Subgroup | Experimental Events | Experimental Total | Control Events | Control Total | Weight | Risk Ratio M-H, Random, 95% CI |
|---|---|---|---|---|---|---|
| **5.10.1 syrup** | | | | | | |
| Bahl 2002 (1) | 37 | 97 | 27 | 104 | 7.6% | 1.47 [0.97, 2.22] |
| Bhatnagar 2004 | 86 | 132 | 79 | 134 | 10.7% | 1.11 [0.92, 1.33] |
| Brooks 2005 | 8 | 43 | 3 | 44 | 1.8% | 2.73 [0.78, 9.61] |
| Brooks 2005 | 6 | 43 | 4 | 44 | 2.0% | 1.53 [0.47, 5.06] |
| Polat 2003 (2) | 2 | 40 | 1 | 36 | 0.6% | 1.80 [0.17, 19.02] |
| Polat 2003 (3) | 3 | 52 | 1 | 54 | 0.6% | 3.12 [0.33, 29.00] |
| Strand 2002 (4) | 73 | 221 | 42 | 224 | 8.7% | 1.76 [1.27, 2.45] |
| Strand 2002 (5) | 87 | 224 | 43 | 225 | 8.9% | 2.03 [1.48, 2.78] |
| **Subtotal (95% CI)** | | **852** | | **865** | **41.0%** | **1.59 [1.20, 2.11]** |
| Total events | 302 | | 200 | | | |
| Heterogeneity: Tau² = 0.07; Chi² = 16.73, df = 7 (P = 0.02); I² = 58% | | | | | | |
| Test for overall effect: Z = 3.20 (P = 0.001) | | | | | | |
| **5.10.2 dispersible tablet** | | | | | | |
| Awasthi 2006 | 86 | 998 | 67 | 984 | 9.1% | 1.27 [0.93, 1.72] |
| Crisinel 2015 | 30 | 42 | 26 | 45 | 9.0% | 1.24 [0.90, 1.69] |
| FischerWalker 2006 | 47 | 538 | 33 | 536 | 7.3% | 1.42 [0.92, 2.18] |
| Larson 2005 | 139 | 534 | 64 | 533 | 9.6% | 2.17 [1.65, 2.84] |
| Shimelis 2008 | 46 | 179 | 24 | 222 | 7.0% | 2.38 [1.51, 3.74] |
| **Subtotal (95% CI)** | | **2291** | | **2320** | **42.0%** | **1.61 [1.22, 2.13]** |
| Total events | 348 | | 214 | | | |
| Heterogeneity: Tau² = 0.07; Chi² = 13.13, df = 4 (P = 0.01); I² = 70% | | | | | | |
| Test for overall effect: Z = 3.37 (P = 0.0008) | | | | | | |
| **5.10.3 ORS** | | | | | | |
| Bahl 2002 (6) | 21 | 109 | 28 | 105 | 6.4% | 0.72 [0.44, 1.19] |
| Wadhwa 2011 | 112 | 248 | 104 | 252 | 10.6% | 1.09 [0.89, 1.34] |
| **Subtotal (95% CI)** | | **357** | | **357** | **17.0%** | **0.95 [0.64, 1.40]** |
| Total events | 133 | | 132 | | | |
| Heterogeneity: Tau² = 0.05; Chi² = 2.33, df = 1 (P = 0.13); I² = 57% | | | | | | |
| Test for overall effect: Z = 0.27 (P = 0.79) | | | | | | |
| **Total (95% CI)** | | **3500** | | **3542** | **100.0%** | **1.46 [1.22, 1.76]** |
| Total events | 783 | | 546 | | | |
| Heterogeneity: Tau² = 0.07; Chi² = 45.53, df = 14 (P < 0.0001); I² = 69% | | | | | | |
| Test for overall effect: Z = 4.03 (P < 0.0001) | | | | | | |
| Test for subgroup differences: Chi² = 5.51, df = 2 (P = 0.06), I² = 63.7% | | | | | | |

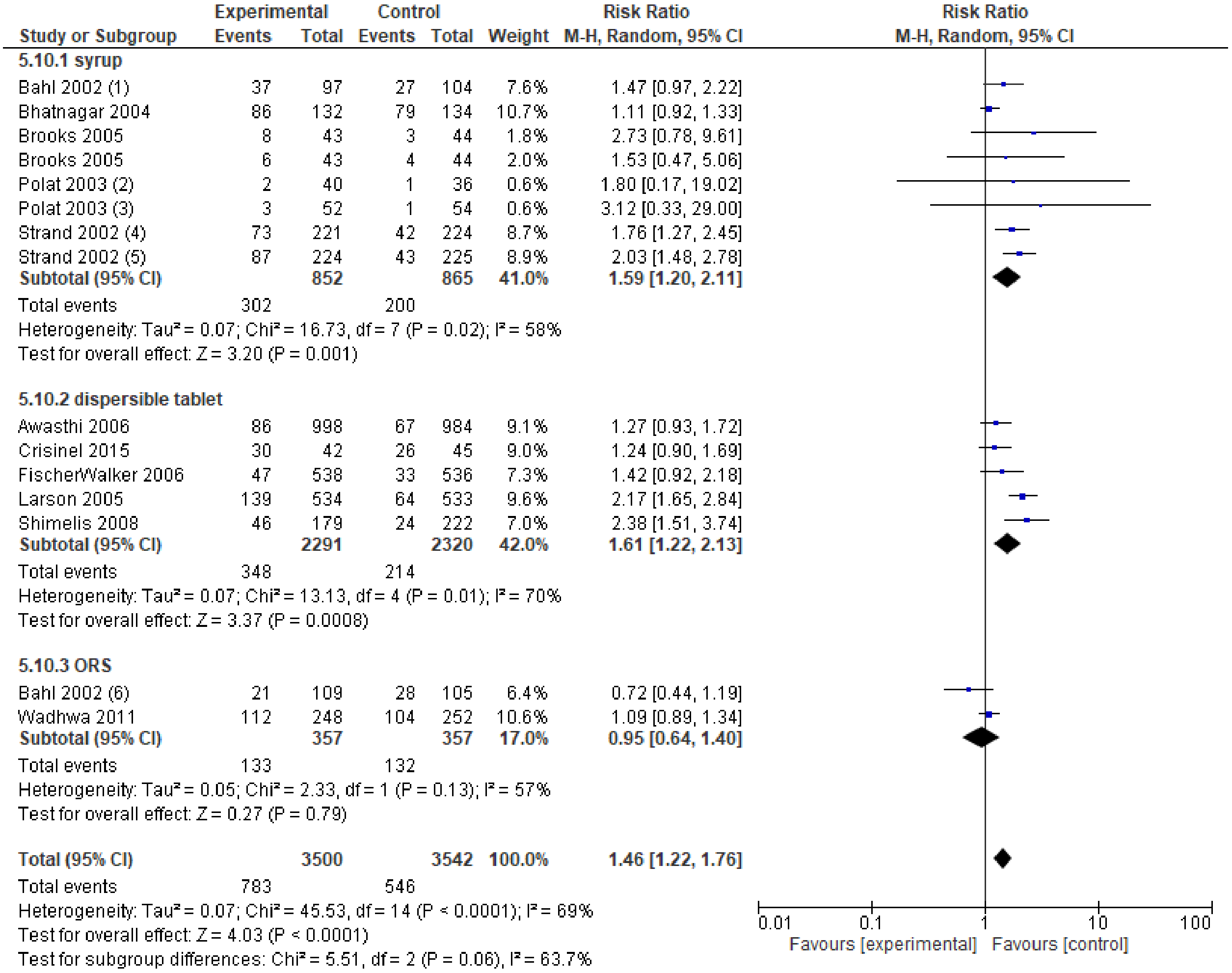


Footnotes
(1) zinc syrup
(2) low zinc at enrollment
(3) normal serum zinc at enrollment
(4) zinc-field workers
(5) zinc-caretakers
(6) zinc-ORS

Figure 2G

Outcome 2: Vomiting (based on age groups)

| Study or Subgroup | Experimental Events | Experimental Total | Control Events | Control Total | Weight | Risk Ratio M-H, Random, 95% CI |
|---|---|---|---|---|---|---|
| **6.10.1 <6 months** | | | | | | |
| Brooks 2005 | 8 | 43 | 3 | 44 | 1.8% | 2.73 [0.78, 9.61] |
| Brooks 2005 | 6 | 43 | 4 | 44 | 2.0% | 1.53 [0.47, 5.06] |
| FischerWalker 2006 | 47 | 538 | 33 | 536 | 7.3% | 1.42 [0.92, 2.18] |
| **Subtotal (95% CI)** | | **624** | | **624** | **11.1%** | **1.52 [1.04, 2.23]** |
| Total events | 61 | | 40 | | | |
| Heterogeneity: Tau² = 0.00; Chi² = 0.93, df = 2 (P = 0.63); I² = 0% | | | | | | |
| Test for overall effect: Z = 2.14 (P = 0.03) | | | | | | |
| **6.10.2 >6 months** | | | | | | |
| Awasthi 2006 | 86 | 998 | 67 | 984 | 9.1% | 1.27 [0.93, 1.72] |
| Bahl 2002 (1) | 21 | 109 | 27 | 104 | 6.3% | 0.74 [0.45, 1.23] |
| Bahl 2002 (2) | 37 | 97 | 28 | 105 | 7.6% | 1.43 [0.95, 2.15] |
| Strand 2002 (3) | 73 | 221 | 42 | 224 | 8.7% | 1.76 [1.27, 2.45] |
| Strand 2002 (4) | 87 | 224 | 43 | 225 | 9.0% | 2.03 [1.48, 2.78] |
| **Subtotal (95% CI)** | | **1649** | | **1642** | **40.7%** | **1.42 [1.06, 1.90]** |
| Total events | 304 | | 207 | | | |
| Heterogeneity: Tau² = 0.08; Chi² = 13.22, df = 4 (P = 0.01); I² = 70% | | | | | | |
| Test for overall effect: Z = 2.32 (P = 0.02) | | | | | | |
| **6.10.3 <6 and >6 months** | | | | | | |
| Bhatnagar 2004 | 86 | 132 | 79 | 134 | 10.8% | 1.11 [0.92, 1.33] |
| Crisinel 2015 | 30 | 42 | 26 | 45 | 9.0% | 1.24 [0.90, 1.69] |
| Larson 2005 | 139 | 534 | 64 | 533 | 9.6% | 2.17 [1.65, 2.84] |
| Polat 2003 (5) | 3 | 52 | 1 | 54 | 0.6% | 3.12 [0.33, 29.00] |
| Polat 2003 (6) | 2 | 40 | 1 | 36 | 0.6% | 1.80 [0.17, 19.02] |
| Shimelis 2008 | 46 | 179 | 24 | 222 | 7.0% | 2.38 [1.51, 3.74] |
| Wadhwa 2011 | 112 | 248 | 104 | 252 | 10.6% | 1.09 [0.89, 1.34] |
| **Subtotal (95% CI)** | | **1227** | | **1276** | **48.2%** | **1.49 [1.10, 2.01]** |
| Total events | 418 | | 299 | | | |
| Heterogeneity: Tau² = 0.10; Chi² = 29.85, df = 6 (P < 0.0001); I² = 80% | | | | | | |
| Test for overall effect: Z = 2.56 (P = 0.01) | | | | | | |
| **Total (95% CI)** | | **3500** | | **3542** | **100.0%** | **1.46 [1.22, 1.76]** |
| Total events | 783 | | 546 | | | |
| Heterogeneity: Tau² = 0.07; Chi² = 44.85, df = 14 (P < 0.0001); I² = 69% | | | | | | |
| Test for overall effect: Z = 4.06 (P < 0.0001) | | | | | | |
| Test for subgroup differences: Chi² = 0.09, df = 2 (P = 0.95), I² = 0% | | | | | | |

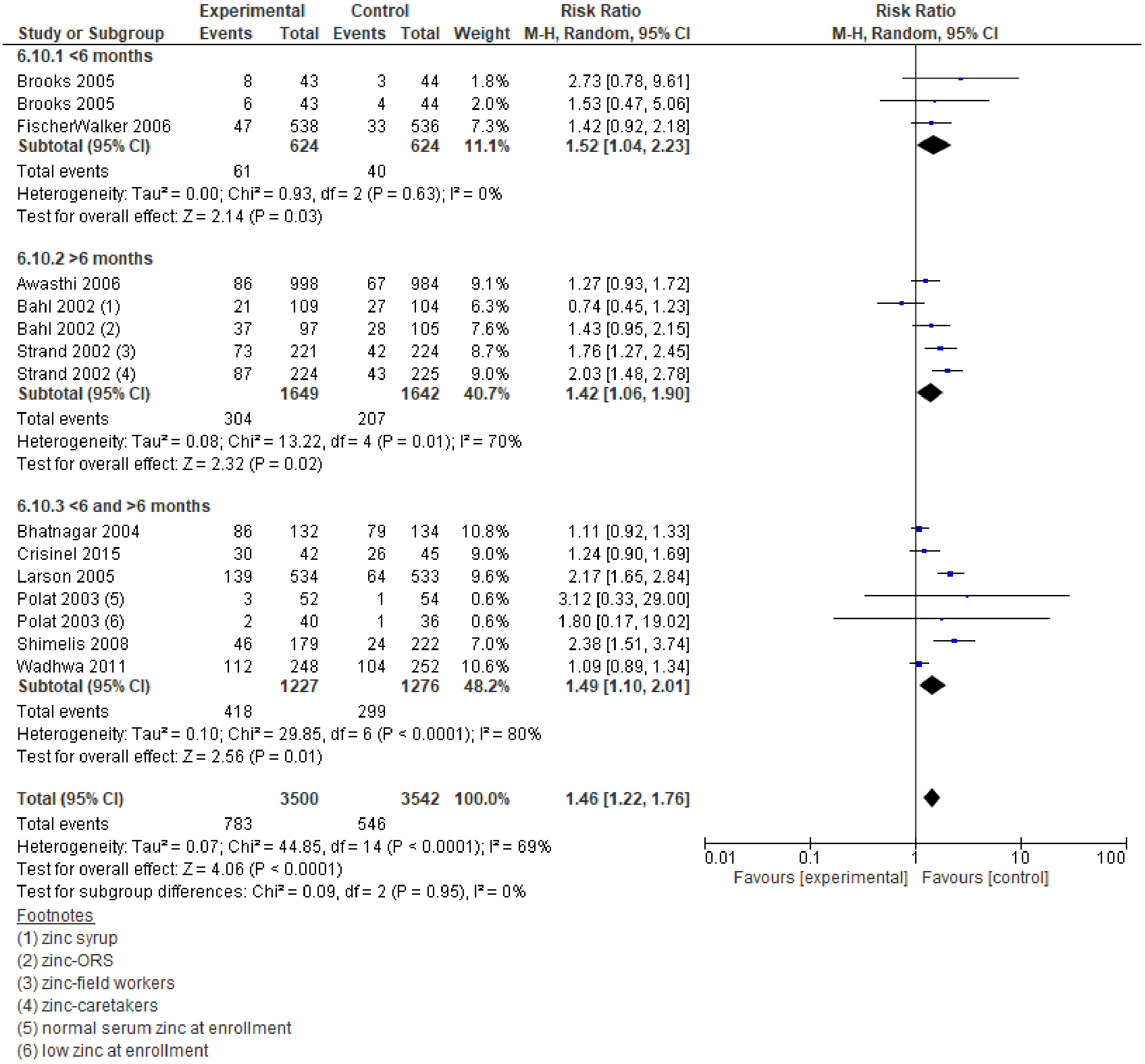


Footnotes

(1) zinc syrup

(2) zinc-ORS

(3) zinc-field workers

(4) zinc-caretakers

(5) normal serum zinc at enrollment

(6) low zinc at enrollment

Figure 2H

Outcome 2: Vomiting (based on study setting)

| Study or Subgroup | Experimental Events | Experimental Total | Control Events | Control Total | Weight | Risk Ratio M-H, Random, 95% CI |
|---|---|---|---|---|---|---|
| **7.10.1 inpatient** | | | | | | |
| Bhatnagar 2004 | 86 | 132 | 79 | 134 | 10.7% | 1.11 [0.92, 1.33] |
| Brooks 2005 | 8 | 43 | 3 | 44 | 1.8% | 2.73 [0.78, 9.61] |
| Brooks 2005 | 6 | 43 | 4 | 44 | 2.0% | 1.53 [0.47, 5.06] |
| Wadhwa 2011 | 112 | 248 | 104 | 252 | 10.6% | 1.09 [0.89, 1.34] |
| **Subtotal (95% CI)** | | **466** | | **474** | **25.2%** | **1.12 [0.97, 1.28]** |
| Total events | 212 | | 190 | | | |
| Heterogeneity: Tau² = 0.00; Chi² = 2.31, df = 3 (P = 0.51); I² = 0% | | | | | | |
| Test for overall effect: Z = 1.59 (P = 0.11) | | | | | | |
| **7.10.2 outpatient** | | | | | | |
| Awasthi 2006 | 86 | 998 | 67 | 984 | 9.1% | 1.27 [0.93, 1.72] |
| Bahl 2002 (1) | 37 | 97 | 27 | 104 | 7.6% | 1.47 [0.97, 2.22] |
| Bahl 2002 (2) | 21 | 109 | 28 | 105 | 6.4% | 0.72 [0.44, 1.19] |
| FischerWalker 2006 | 47 | 538 | 33 | 536 | 7.3% | 1.42 [0.92, 2.18] |
| Polat 2003 (3) | 3 | 52 | 1 | 54 | 0.6% | 3.12 [0.33, 29.00] |
| Polat 2003 (4) | 2 | 40 | 1 | 36 | 0.6% | 1.80 [0.17, 19.02] |
| Shimelis 2008 | 46 | 179 | 24 | 222 | 7.0% | 2.38 [1.51, 3.74] |
| Strand 2002 (5) | 73 | 221 | 42 | 224 | 8.7% | 1.76 [1.27, 2.45] |
| Strand 2002 (6) | 87 | 224 | 43 | 225 | 8.9% | 2.03 [1.48, 2.78] |
| **Subtotal (95% CI)** | | **2458** | | **2490** | **56.3%** | **1.53 [1.21, 1.93]** |
| Total events | 402 | | 266 | | | |
| Heterogeneity: Tau² = 0.06; Chi² = 18.15, df = 8 (P = 0.02); I² = 56% | | | | | | |
| Test for overall effect: Z = 3.57 (P = 0.0004) | | | | | | |
| **7.10.3 inpatient and outpatient** | | | | | | |
| Crisinel 2015 | 30 | 42 | 26 | 45 | 9.0% | 1.24 [0.90, 1.69] |
| Larson 2005 | 139 | 534 | 64 | 533 | 9.6% | 2.17 [1.65, 2.84] |
| **Subtotal (95% CI)** | | **576** | | **578** | **18.6%** | **1.65 [0.91, 2.97]** |
| Total events | 169 | | 90 | | | |
| Heterogeneity: Tau² = 0.16; Chi² = 8.14, df = 1 (P = 0.004); I² = 88% | | | | | | |
| Test for overall effect: Z = 1.65 (P = 0.10) | | | | | | |
| **Total (95% CI)** | | **3500** | | **3542** | **100.0%** | **1.46 [1.22, 1.76]** |
| Total events | 783 | | 546 | | | |
| Heterogeneity: Tau² = 0.07; Chi² = 45.53, df = 14 (P < 0.0001); I² = 69% | | | | | | |
| Test for overall effect: Z = 4.03 (P < 0.0001) | | | | | | |
| Test for subgroup differences: Chi² = 6.24, df = 2 (P = 0.04), I² = 68.0% | | | | | | |

Risk Ratio
M-H, Random, 95% CI

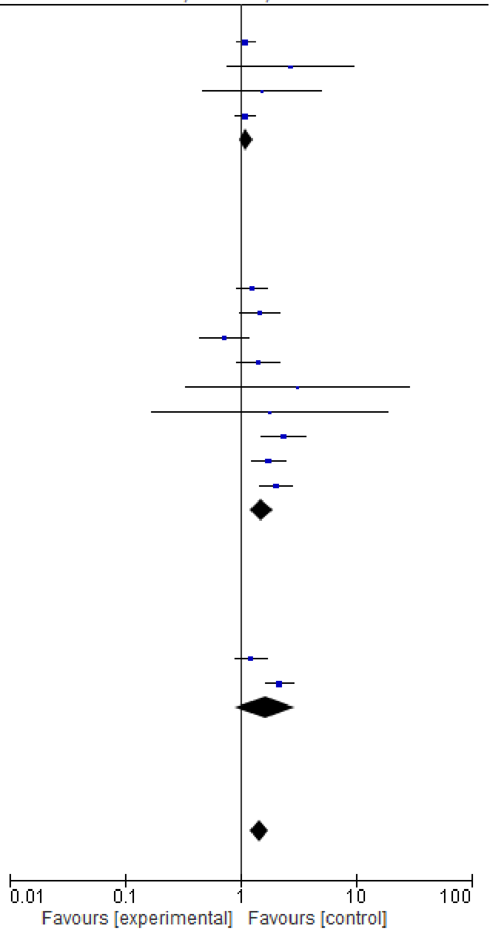


Footnotes
(1) zinc syrup
(2) zinc-ORS
(3) normal serum zinc at enrollment
(4) low zinc at enrollment
(5) zinc-field workers
(6) zinc-caretakers

Figure 2l

Outcome 2- Vomiting sensitivity analysis

| Study or Subgroup | Experimental Events | Experimental Total | Control Events | Control Total | Weight | Risk Ratio M-H, Random, 95% CI |
|---|---|---|---|---|---|---|
| Bahl 2002 (1) | 37 | 97 | 27 | 104 | 10.2% | 1.47 [0.97, 2.22] |
| Bahl 2002 (2) | 21 | 109 | 28 | 105 | 8.6% | 0.72 [0.44, 1.19] |
| Bhatnagar 2004 | 86 | 132 | 79 | 134 | 14.7% | 1.11 [0.92, 1.33] |
| Brooks 2005 (3) | 8 | 43 | 3 | 44 | 2.4% | 2.73 [0.78, 9.61] |
| Brooks 2005 (4) | 6 | 43 | 4 | 45 | 2.6% | 1.57 [0.48, 5.18] |
| Crisinel 2015 | 30 | 42 | 26 | 45 | 12.2% | 1.24 [0.90, 1.69] |
| FischerWalker 2006 | 47 | 538 | 33 | 536 | 9.9% | 1.42 [0.92, 2.18] |
| Larson 2005 | 139 | 534 | 64 | 533 | 13.1% | 2.17 [1.65, 2.84] |
| Strand 2002 (5) | 73 | 221 | 42 | 224 | 11.8% | 1.76 [1.27, 2.45] |
| Wadhwa 2011 | 112 | 248 | 104 | 252 | 14.5% | 1.09 [0.89, 1.34] |
| **Total (95% CI)** | | **2007** | | **2022** | **100.0%** | **1.35 [1.10, 1.67]** |
| Total events | 559 | | 410 | | | |

Heterogeneity: Tau² = 0.07; Chi² = 31.57, df = 9 (P = 0.0002); I² = 71%
Test for overall effect: Z = 2.82 (P = 0.005)

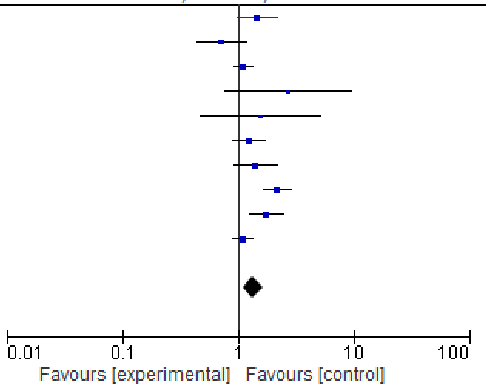


Footnotes
(1) zinc syrup
(2) zinc-ORS
(3) 5mg
(4) 20mg
(5) zinc-field workers

## Mortality

Figure 3A

Outcome 3: Mortality (subgroups by dose of zinc)

| Study or Subgroup | Experimental Events | Experimental Total | Control Events | Control Total | Weight | Risk Ratio M-H, Fixed, 95% CI |
|---|---|---|---|---|---|---|
| **3.17.1 20mg** | | | | | | |
| Shimelis 2008 | 0 | 179 | 0 | 222 | | Not estimable |
| **Subtotal (95% CI)** | | **179** | | **222** | | **Not estimable** |
| Total events | 0 | | 0 | | | |
| Heterogeneity: Not applicable | | | | | | |
| Test for overall effect: Not applicable | | | | | | |
| **3.17.2 Dose by weight or age** | | | | | | |
| Brooks 2005 (1) | 0 | 174 | 0 | 89 | | Not estimable |
| Patel 2009 | 1 | 248 | 1 | 123 | 57.2% | 0.50 [0.03, 7.86] |
| **Subtotal (95% CI)** | | **422** | | **212** | **57.2%** | **0.50 [0.03, 7.86]** |
| Total events | 1 | | 1 | | | |
| Heterogeneity: Not applicable | | | | | | |
| Test for overall effect: Z = 0.50 (P = 0.62) | | | | | | |
| **3.17.3 10mg** | | | | | | |
| FischerWalker 2006 | 1 | 538 | 1 | 536 | 42.8% | 1.00 [0.06, 15.89] |
| **Subtotal (95% CI)** | | **538** | | **536** | **42.8%** | **1.00 [0.06, 15.89]** |
| Total events | 1 | | 1 | | | |
| Heterogeneity: Not applicable | | | | | | |
| Test for overall effect: Z = 0.00 (P = 1.00) | | | | | | |
| **Total (95% CI)** | | **1139** | | **970** | **100.0%** | **0.71 [0.10, 4.88]** |
| Total events | 2 | | 2 | | | |

Heterogeneity: Chi² = 0.12, df = 1 (P = 0.73); I² = 0%
Test for overall effect: Z = 0.35 (P = 0.73)
Test for subgroup differences: Chi² = 0.12, df = 1 (P = 0.73), I² = 0%

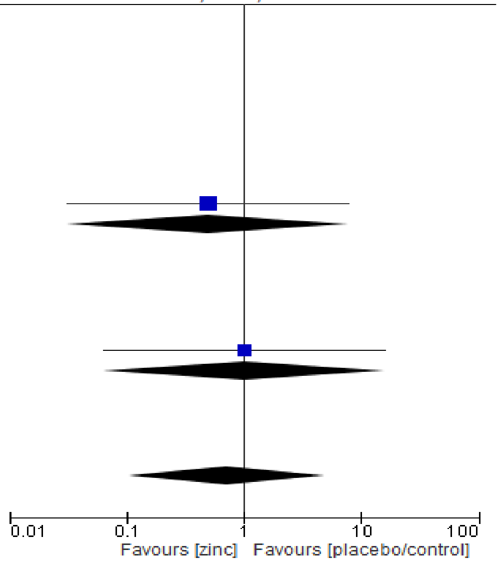


Footnotes
(1) 2 arms- 5mg and 20mg

Figure 3B

Outcome 3: Mortality (subgroups by zinc formulation)

| Study or Subgroup | Experimental Events | Experimental Total | Control Events | Control Total | Weight | Risk Ratio M-H, Fixed, 95% CI |
|---|---|---|---|---|---|---|
| **4.17.1 Zinc acetate** | | | | | | |
| Brooks 2005 | 0 | 174 | 0 | 89 | | Not estimable |
| **Subtotal (95% CI)** | | **174** | | **89** | | **Not estimable** |
| Total events | 0 | | 0 | | | |
| Heterogeneity: Not applicable | | | | | | |
| Test for overall effect: Not applicable | | | | | | |
| **4.17.2 Zinc sulfate** | | | | | | |
| Patel 2009 | 1 | 248 | 1 | 123 | 57.2% | 0.50 [0.03, 7.86] |
| **Subtotal (95% CI)** | | **248** | | **123** | **57.2%** | **0.50 [0.03, 7.86]** |
| Total events | 1 | | 1 | | | |
| Heterogeneity: Not applicable | | | | | | |
| Test for overall effect: Z = 0.50 (P = 0.62) | | | | | | |
| **4.17.3 Unspecified** | | | | | | |
| FischerWalker 2006 | 1 | 538 | 1 | 536 | 42.8% | 1.00 [0.06, 15.89] |
| Shimelis 2008 | 0 | 179 | 0 | 222 | | Not estimable |
| **Subtotal (95% CI)** | | **717** | | **758** | **42.8%** | **1.00 [0.06, 15.89]** |
| Total events | 1 | | 1 | | | |
| Heterogeneity: Not applicable | | | | | | |
| Test for overall effect: Z = 0.00 (P = 1.00) | | | | | | |
| **Total (95% CI)** | | **1139** | | **970** | **100.0%** | **0.71 [0.10, 4.88]** |
| Total events | 2 | | 2 | | | |
| Heterogeneity: Chi² = 0.12, df = 1 (P = 0.73); I² = 0% | | | | | | |
| Test for overall effect: Z = 0.35 (P = 0.73) | | | | | | |
| Test for subgroup differences: Chi² = 0.12, df = 1 (P = 0.73), I² = 0% | | | | | | |

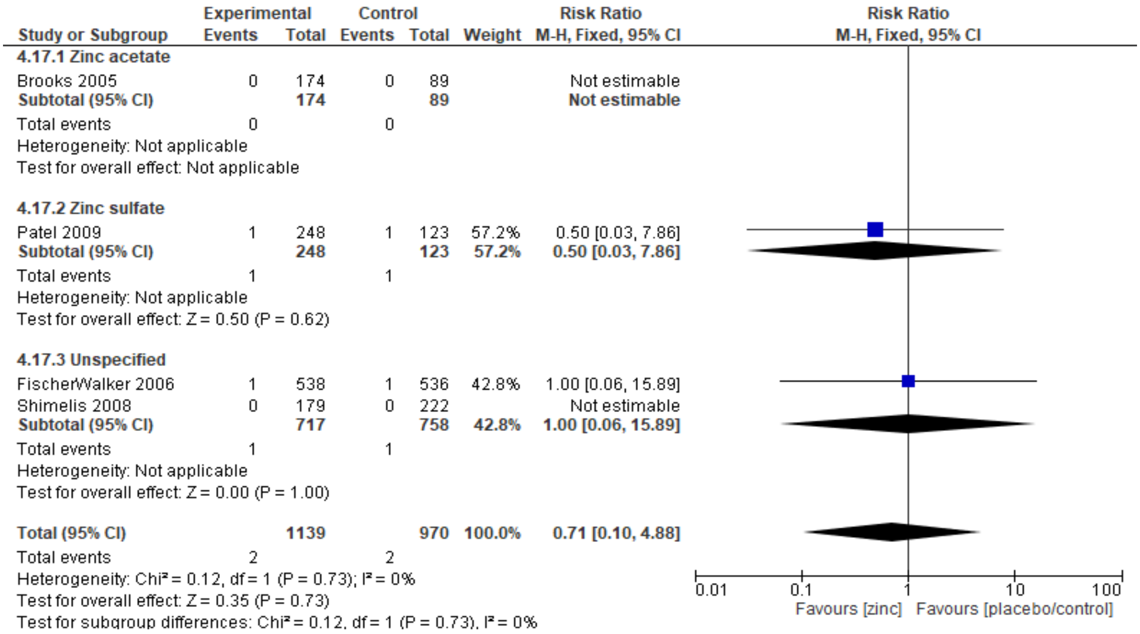


Figure 3C

Outcome 3: Mortality (subgroups by duration of zinc supplementation)

| Study or Subgroup | Experimental Events | Experimental Total | Control Events | Control Total | Weight | Risk Ratio M-H, Fixed, 95% CI |
|---|---|---|---|---|---|---|
| **8.17.1 14d** | | | | | | |
| FischerWalker 2006 | 1 | 538 | 1 | 536 | 42.8% | 1.00 [0.06, 15.89] |
| Patel 2009 | 1 | 248 | 1 | 123 | 57.2% | 0.50 [0.03, 7.86] |
| Shimelis 2008 | 0 | 179 | 0 | 222 | | Not estimable |
| **Subtotal (95% CI)** | | **965** | | **881** | **100.0%** | **0.71 [0.10, 4.88]** |
| Total events | 2 | | 2 | | | |
| Heterogeneity: Chi² = 0.12, df = 1 (P = 0.73); I² = 0% | | | | | | |
| Test for overall effect: Z = 0.35 (P = 0.73) | | | | | | |
| **8.17.2 Unspecified** | | | | | | |
| Brooks 2005 | 0 | 174 | 0 | 89 | | Not estimable |
| **Subtotal (95% CI)** | | **174** | | **89** | | **Not estimable** |
| Total events | 0 | | 0 | | | |
| Heterogeneity: Not applicable | | | | | | |
| Test for overall effect: Not applicable | | | | | | |
| **Total (95% CI)** | | **1139** | | **970** | **100.0%** | **0.71 [0.10, 4.88]** |
| Total events | 2 | | 2 | | | |
| Heterogeneity: Chi² = 0.12, df = 1 (P = 0.73); I² = 0% | | | | | | |
| Test for overall effect: Z = 0.35 (P = 0.73) | | | | | | |
| Test for subgroup differences: Not applicable | | | | | | |

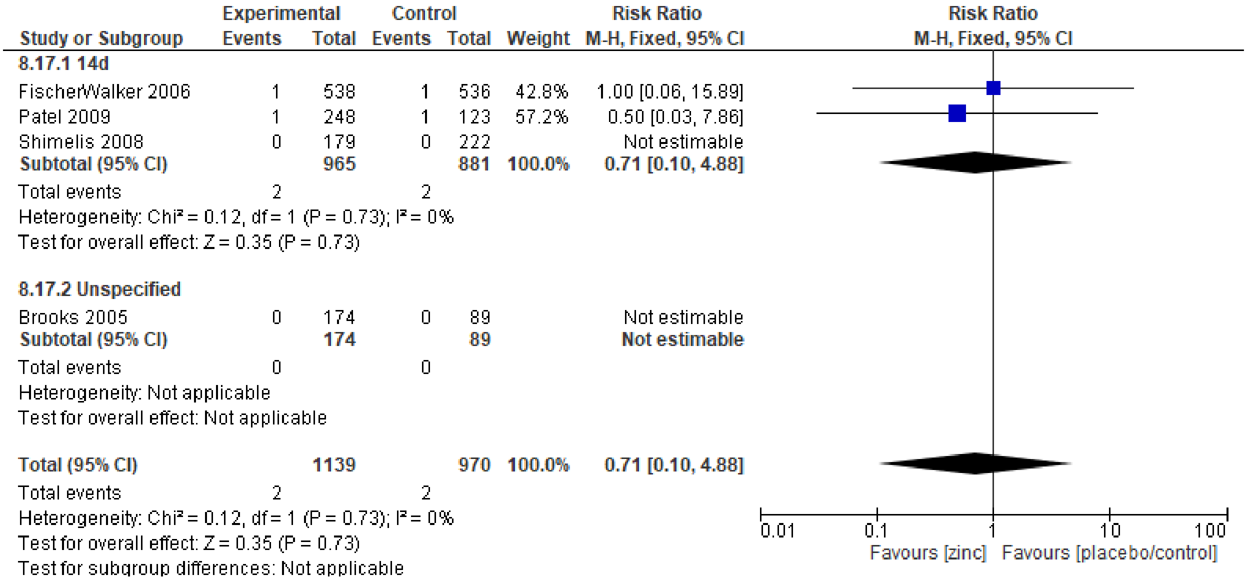

Figure 3D

## Outcome 3: Mortality (subgroups by definition of diarrhea)

| Study or Subgroup | Experimental Events | Experimental Total | Control Events | Control Total | Weight | Risk Ratio M-H, Fixed, 95% CI |
|---|---|---|---|---|---|---|
| **13.17.1 WHO definition** | | | | | | |
| Brooks 2005 | 0 | 174 | 0 | 89 | | Not estimable |
| FischerWalker 2006 | 1 | 538 | 1 | 536 | 42.8% | 1.00 [0.06, 15.89] |
| Patel 2009 | 1 | 248 | 1 | 123 | 57.2% | 0.50 [0.03, 7.86] |
| **Subtotal (95% CI)** | | **960** | | **748** | **100.0%** | **0.71 [0.10, 4.88]** |
| Total events | 2 | | 2 | | | |
| Heterogeneity: Chi² = 0.12, df = 1 (P = 0.73); I² = 0% | | | | | | |
| Test for overall effect: Z = 0.35 (P = 0.73) | | | | | | |
| **13.17.2 Other definition** | | | | | | |
| Shimelis 2008 | 0 | 179 | 0 | 222 | | Not estimable |
| **Subtotal (95% CI)** | | **179** | | **222** | | **Not estimable** |
| Total events | 0 | | 0 | | | |
| Heterogeneity: Not applicable | | | | | | |
| Test for overall effect: Not applicable | | | | | | |
| **Total (95% CI)** | | **1139** | | **970** | **100.0%** | **0.71 [0.10, 4.88]** |
| Total events | 2 | | 2 | | | |
| Heterogeneity: Chi² = 0.12, df = 1 (P = 0.73); I² = 0% | | | | | | |
| Test for overall effect: Z = 0.35 (P = 0.73) | | | | | | |
| Test for subgroup differences: Not applicable | | | | | | |

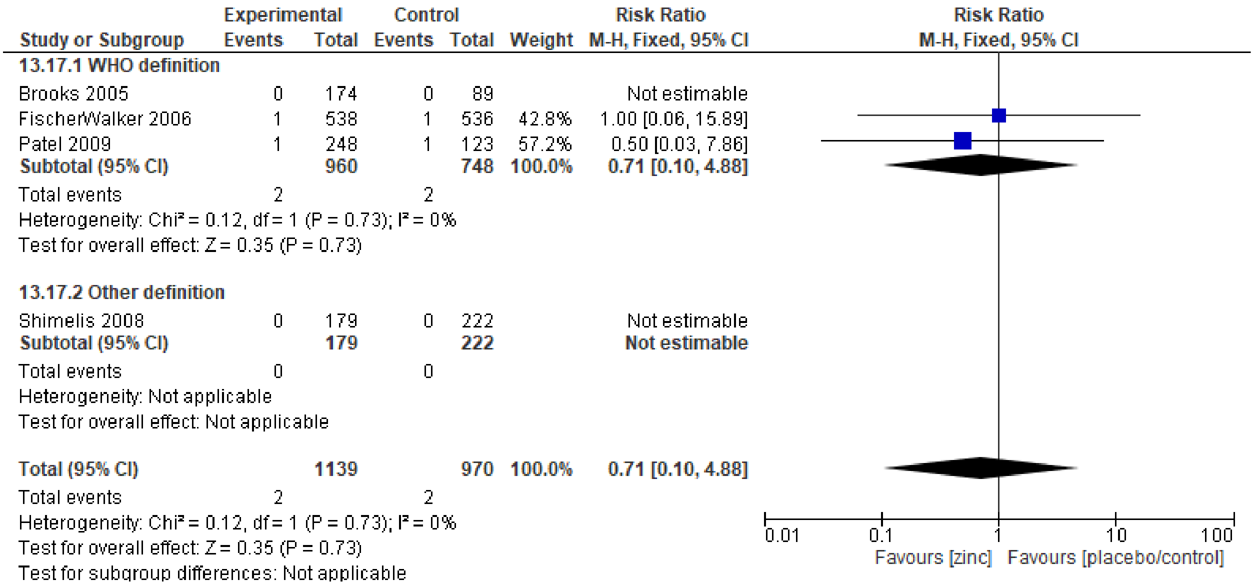


Figure 3E

## Outcome 3: Mortality (subgroups by age group)

| Study or Subgroup | Experimental Events | Experimental Total | Control Events | Control Total | Weight | Risk Ratio M-H, Fixed, 95% CI |
|---|---|---|---|---|---|---|
| **6.17.1 <6 months** | | | | | | |
| Brooks 2005 | 0 | 174 | 0 | 89 | | Not estimable |
| FischerWalker 2006 | 1 | 538 | 1 | 536 | 42.8% | 1.00 [0.06, 15.89] |
| **Subtotal (95% CI)** | | **712** | | **625** | **42.8%** | **1.00 [0.06, 15.89]** |
| Total events | 1 | | 1 | | | |
| Heterogeneity: Not applicable | | | | | | |
| Test for overall effect: Z = 0.00 (P = 1.00) | | | | | | |
| **6.17.2 > 6months** | | | | | | |
| Patel 2009 | 1 | 248 | 1 | 123 | 57.2% | 0.50 [0.03, 7.86] |
| **Subtotal (95% CI)** | | **248** | | **123** | **57.2%** | **0.50 [0.03, 7.86]** |
| Total events | 1 | | 1 | | | |
| Heterogeneity: Not applicable | | | | | | |
| Test for overall effect: Z = 0.50 (P = 0.62) | | | | | | |
| **6.17.3 <6 and >6 months** | | | | | | |
| Shimelis 2008 | 0 | 179 | 0 | 222 | | Not estimable |
| **Subtotal (95% CI)** | | **179** | | **222** | | **Not estimable** |
| Total events | 0 | | 0 | | | |
| Heterogeneity: Not applicable | | | | | | |
| Test for overall effect: Not applicable | | | | | | |
| **Total (95% CI)** | | **1139** | | **970** | **100.0%** | **0.71 [0.10, 4.88]** |
| Total events | 2 | | 2 | | | |
| Heterogeneity: Chi² = 0.12, df = 1 (P = 0.73); I² = 0% | | | | | | |
| Test for overall effect: Z = 0.35 (P = 0.73) | | | | | | |
| Test for subgroup differences: Chi² = 0.12, df = 1 (P = 0.73), I² = 0% | | | | | | |

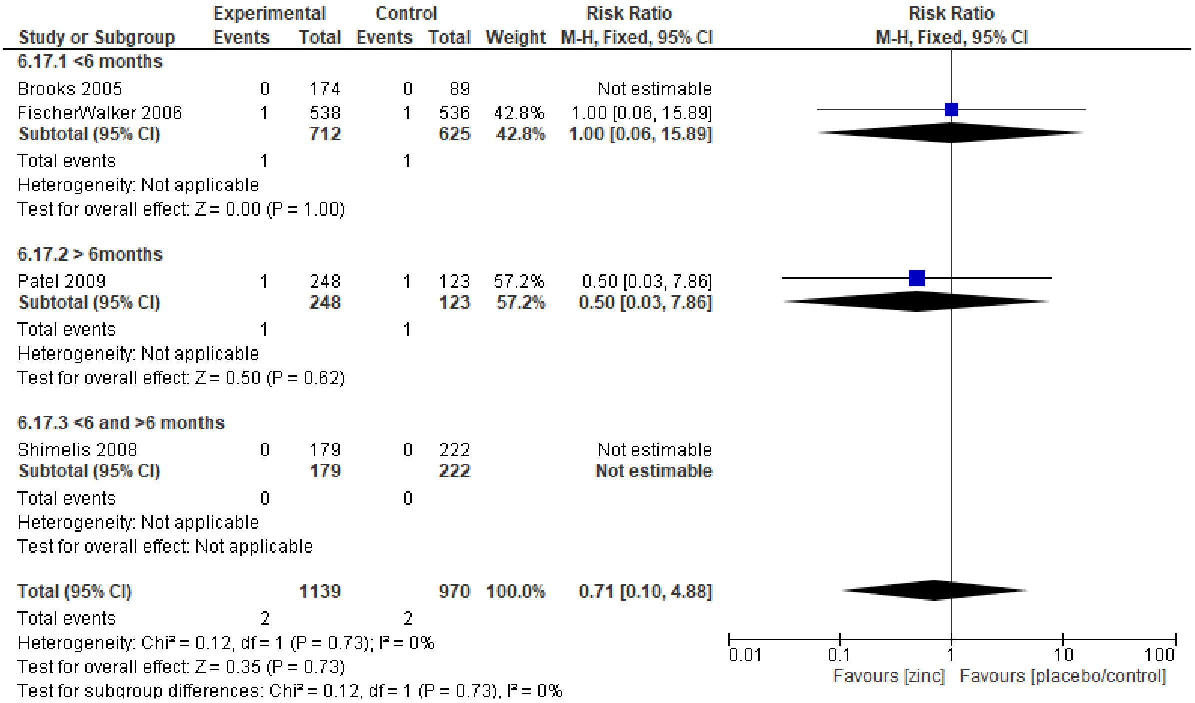

Figure 3F

Outcome: Mortality (subgroups by study setting)

| Study or Subgroup | Experimental Events | Experimental Total | Control Events | Control Total | Weight | Risk Ratio M-H, Fixed, 95% CI |
|---|---|---|---|---|---|---|
| **7.17.1 Inpatient** | | | | | | |
| Brooks 2005 | 0 | 174 | 0 | 89 | | Not estimable |
| **Subtotal (95% CI)** | | **174** | | **89** | | **Not estimable** |
| Total events | 0 | | 0 | | | |
| Heterogeneity: Not applicable | | | | | | |
| Test for overall effect: Not applicable | | | | | | |
| **7.17.2 Outpatient** | | | | | | |
| FischerWalker 2006 | 1 | 538 | 1 | 536 | 42.8% | 1.00 [0.06, 15.89] |
| Shimelis 2008 | 0 | 179 | 0 | 222 | | Not estimable |
| **Subtotal (95% CI)** | | **717** | | **758** | **42.8%** | **1.00 [0.06, 15.89]** |
| Total events | 1 | | 1 | | | |
| Heterogeneity: Not applicable | | | | | | |
| Test for overall effect: Z = 0.00 (P = 1.00) | | | | | | |
| **7.17.3 Inpatient and outpatient** | | | | | | |
| Patel 2009 | 1 | 248 | 1 | 123 | 57.2% | 0.50 [0.03, 7.86] |
| **Subtotal (95% CI)** | | **248** | | **123** | **57.2%** | **0.50 [0.03, 7.86]** |
| Total events | 1 | | 1 | | | |
| Heterogeneity: Not applicable | | | | | | |
| Test for overall effect: Z = 0.50 (P = 0.62) | | | | | | |
| **Total (95% CI)** | | **1139** | | **970** | **100.0%** | **0.71 [0.10, 4.88]** |
| Total events | 2 | | 2 | | | |
| Heterogeneity: Chi² = 0.12, df = 1 (P = 0.73); I² = 0% | | | | | | |
| Test for overall effect: Z = 0.35 (P = 0.73) | | | | | | |
| Test for subgroup differences: Chi² = 0.12, df = 1 (P = 0.73), I² = 0% | | | | | | |

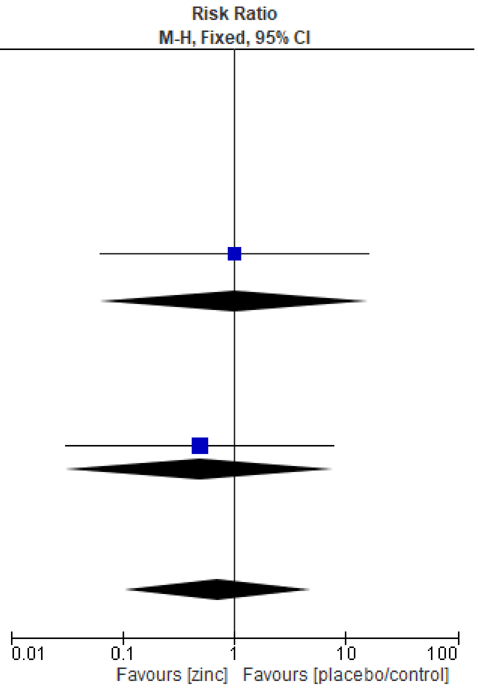


Figure 3G

Outcome 3- Mortality (sensitivity analysis)

| Study or Subgroup | Experimental Events | Experimental Total | Control Events | Control Total | Weight | Risk Ratio M-H, Fixed, 95% CI |
|---|---|---|---|---|---|---|
| Brooks 2005 | 0 | 174 | 0 | 89 | | Not estimable |
| FischerWalker 2006 | 1 | 538 | 1 | 536 | 100.0% | 1.00 [0.06, 15.89] |
| **Total (95% CI)** | | **712** | | **625** | **100.0%** | **1.00 [0.06, 15.89]** |
| Total events | 1 | | 1 | | | |
| Heterogeneity: Not applicable | | | | | | |
| Test for overall effect: Z = 0.00 (P = 1.00) | | | | | | |

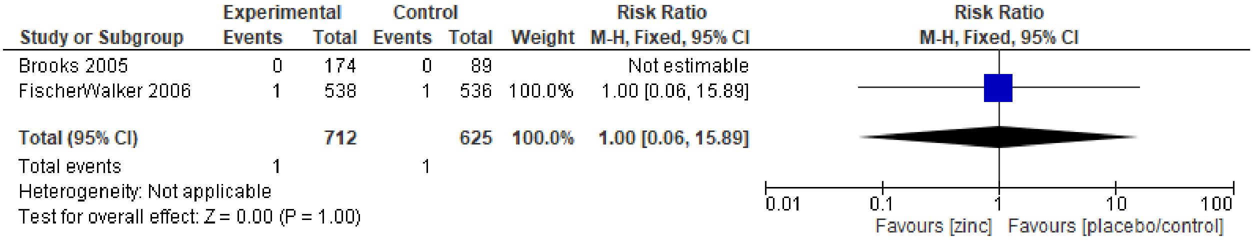

**Duration of Diarrhea**

Figure 4A

Outcome 4: Duration of diarrhea (subgroup analysis by definition of diarrhea)

| | Experimental | | | Control | | | | Mean Difference |
|---|---|---|---|---|---|---|---|---|
| **Study or Subgroup** | **Mean** | **SD** | **Total** | **Mean** | **SD** | **Total** | **Weight** | **IV, Random, 95% CI** |
| **13.3.1 WHO definition of diarrhea** | | | | | | | | |
| Al Sonboli 2003 | 28.8 | 19.2 | 37 | 60 | 43.2 | 37 | 3.4% | -31.20 [-46.43, -15.97] |
| Bhatnagar 2004 | 55.8 | 37 | 132 | 64.6 | 45.6 | 134 | 4.4% | -8.80 [-18.77, 1.17] |
| Boran 2005 | 72.48 | 48 | 145 | 88.08 | 76.8 | 120 | 3.3% | -15.60 [-31.41, 0.21] |
| Brooks 2005 (1) | 120 | 12.2 | 86 | 120 | 12.2 | 45 | 5.4% | 0.00 [-4.40, 4.40] |
| Brooks 2005 (2) | 120 | 12.2 | 85 | 120 | 12.2 | 44 | 5.4% | 0.00 [-4.44, 4.44] |
| Crisinel 2015 | 62 | 50.32 | 39 | 64.9 | 58.2 | 40 | 2.1% | -2.90 [-26.87, 21.07] |
| Dalgic 2011 | 81.84 | 33.12 | 60 | 128.4 | 43.2 | 60 | 3.7% | -46.56 [-60.33, -32.79] |
| Dutta 2011 | 64.1 | 21.7 | 41 | 88.2 | 23 | 43 | 4.5% | -24.10 [-33.66, -14.54] |
| FischerWalker 2006 | 198 | 90.72 | 538 | 186 | 82.56 | 536 | 4.3% | 12.00 [1.63, 22.37] |
| FischerWalker 2008 (3) | 141.6 | 108 | 100 | 170.4 | 124.8 | 105 | 1.4% | -28.80 [-60.70, 3.10] |
| FischerWalker 2008 (4) | 108 | 84 | 520 | 136.8 | 108 | 527 | 4.1% | -28.80 [-40.51, -17.09] |
| Kakar 2022 | 27.63 | 14.22 | 72 | 38.86 | 20.7 | 72 | 5.2% | -11.23 [-17.03, -5.43] |
| Patel 2009 | 64.4 | 37.8 | 248 | 62.2 | 33.5 | 247 | 5.1% | 2.20 [-4.09, 8.49] |
| Patro 2010 | 78.75 | 37 | 69 | 71.5 | 38.6 | 72 | 3.9% | 7.25 [-5.23, 19.73] |
| Rerksuppaphol 2020 | 34.7 | 24.5 | 43 | 56 | 33.7 | 43 | 3.9% | -21.30 [-33.75, -8.85] |
| Yalcin 2022 | 80.1 | 10.3 | 28 | 84.5 | 10.7 | 22 | 5.2% | -4.40 [-10.28, 1.48] |
| **Subtotal (95% CI)** | | | **2243** | | | **2147** | **65.3%** | **-11.26 [-17.51, -5.00]** |
| Heterogeneity: Tau² = 125.50; Chi² = 121.55, df = 15 (P < 0.00001); I² = 88% | | | | | | | | |
| Test for overall effect: Z = 3.53 (P = 0.0004) | | | | | | | | |
| **13.3.2 Other definition** | | | | | | | | |
| Bahl 2002 (5) | 38.4 | 52.8 | 402 | 40.8 | 60 | 201 | 4.5% | -2.40 [-12.17, 7.37] |
| Bahl 2002 (6) | 33.6 | 57.6 | 404 | 40.8 | 60 | 200 | 4.4% | -7.20 [-17.23, 2.83] |
| Dutta 2000 | 70.4 | 10 | 44 | 103.4 | 17.1 | 36 | 5.1% | -33.00 [-39.32, -26.68] |
| Polat 2003 (7) | 105.6 | 31.2 | 40 | 146.4 | 40.8 | 36 | 3.2% | -40.80 [-57.27, -24.33] |
| Polat 2003 (8) | 112.8 | 33.6 | 52 | 124.8 | 38.4 | 54 | 3.7% | -12.00 [-25.72, 1.72] |
| Roy 2008 | 64.1 | 2.86 | 82 | 72.8 | 2.86 | 82 | 5.7% | -8.70 [-9.58, -7.82] |
| **Subtotal (95% CI)** | | | **1024** | | | **609** | **26.5%** | **-16.69 [-27.78, -5.60]** |
| Heterogeneity: Tau² = 164.59; Chi² = 72.07, df = 5 (P < 0.00001); I² = 93% | | | | | | | | |
| Test for overall effect: Z = 2.95 (P = 0.003) | | | | | | | | |
| **13.3.3 Not mentioned** | | | | | | | | |
| Gregorio 2007 | 71.52 | 22.08 | 59 | 88.08 | 38.4 | 57 | 4.1% | -16.56 [-28.01, -5.11] |
| Yazar 2016 | 86.4 | 30.8 | 55 | 114.3 | 30.9 | 55 | 4.1% | -27.90 [-39.43, -16.37] |
| **Subtotal (95% CI)** | | | **114** | | | **112** | **8.2%** | **-22.21 [-33.32, -11.10]** |
| Heterogeneity: Tau² = 29.93; Chi² = 1.87, df = 1 (P = 0.17); I² = 47% | | | | | | | | |
| Test for overall effect: Z = 3.92 (P < 0.0001) | | | | | | | | |
| **Total (95% CI)** | | | **3381** | | | **2868** | **100.0%** | **-13.27 [-17.66, -8.89]** |
| Heterogeneity: Tau² = 87.49; Chi² = 216.51, df = 23 (P < 0.00001); I² = 89% | | | | | | | | |
| Test for overall effect: Z = 5.93 (P < 0.00001) | | | | | | | | |
| Test for subgroup differences: Chi² = 3.03, df = 2 (P = 0.22), I² = 34.0% | | | | | | | | |

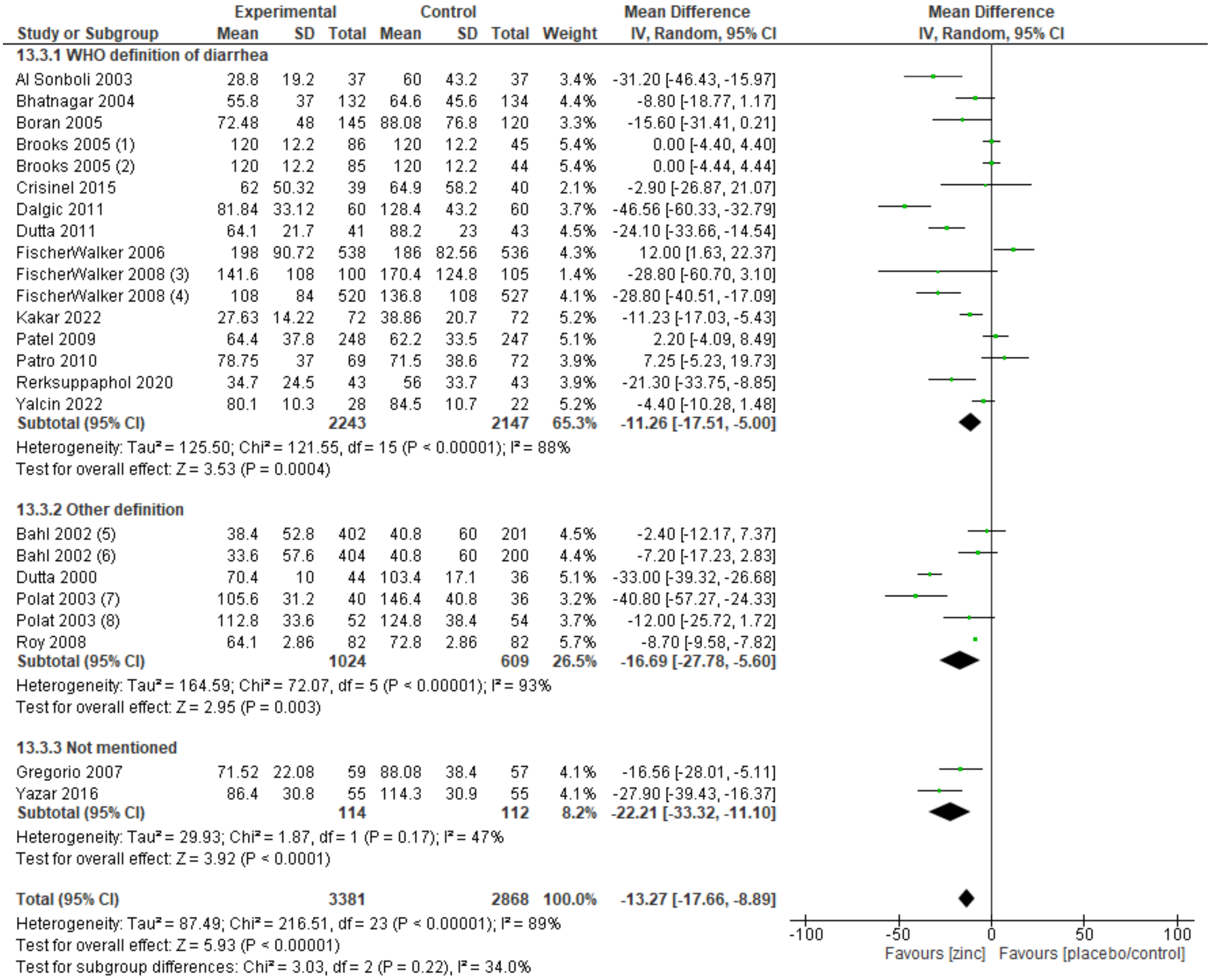


Footnotes

(1) 20mg

(2) 5mg

(3) <12 months

(4) 12-59 months

(5) zinc-ORS

(6) zinc syrup

(7) low serum zinc

(8) normal serum zinc

Figure 4B

Outcome 4: Duration of diarrhea (subgroup analysis by dose of zinc administered)

| Study or Subgroup | Experimental Mean | Experimental SD | Experimental Total | Control Mean | Control SD | Control Total | Weight | Mean Difference IV, Random, 95% CI |
|---|---|---|---|---|---|---|---|---|
| **3.2.1 5mg** | | | | | | | | |
| Brooks 2005 (1) | 120 | 12.2 | 86 | 120 | 12.2 | 44 | 5.4% | 0.00 [-4.43, 4.43] |
| **Subtotal (95% CI)** | | | **86** | | | **44** | **5.4%** | **0.00 [-4.43, 4.43]** |
| Heterogeneity: Not applicable | | | | | | | | |
| Test for overall effect: Z = 0.00 (P = 1.00) | | | | | | | | |
| **3.2.2 10mg** | | | | | | | | |
| FischerWalker 2006 | 198 | 90.72 | 538 | 186 | 82.56 | 536 | 4.3% | 12.00 [1.63, 22.37] |
| **Subtotal (95% CI)** | | | **538** | | | **536** | **4.3%** | **12.00 [1.63, 22.37]** |
| Heterogeneity: Not applicable | | | | | | | | |
| Test for overall effect: Z = 2.27 (P = 0.02) | | | | | | | | |
| **3.2.3 15mg** | | | | | | | | |
| Rerksuppaphol 2020 | 34.7 | 24.5 | 43 | 56 | 33.7 | 43 | 3.9% | -21.30 [-33.75, -8.85] |
| Yazar 2016 | 86.4 | 30.8 | 55 | 114.3 | 30.9 | 55 | 4.1% | -27.90 [-39.43, -16.37] |
| **Subtotal (95% CI)** | | | **98** | | | **98** | **8.0%** | **-24.85 [-33.31, -16.39]** |
| Heterogeneity: Tau² = 0.00; Chi² = 0.58, df = 1 (P = 0.45); I² = 0% | | | | | | | | |
| Test for overall effect: Z = 5.76 (P < 0.00001) | | | | | | | | |
| **3.2.4 20mg** | | | | | | | | |
| Brooks 2005 (2) | 120 | 12.2 | 85 | 120 | 12.2 | 45 | 5.4% | 0.00 [-4.41, 4.41] |
| Dutta 2011 | 64.1 | 21.7 | 41 | 88.2 | 23 | 43 | 4.5% | -24.10 [-33.66, -14.54] |
| FischerWalker 2008 (3) | 108 | 84 | 520 | 136.8 | 108 | 527 | 4.1% | -28.80 [-40.51, -17.09] |
| FischerWalker 2008 (4) | 141.6 | 108 | 100 | 170.4 | 124.8 | 105 | 1.4% | -28.80 [-60.70, 3.10] |
| Gregorio 2007 | 71.52 | 22.08 | 59 | 88.08 | 38.4 | 57 | 4.1% | -16.56 [-28.01, -5.11] |
| Polat 2003 (5) | 105.6 | 31.2 | 40 | 146.4 | 40.8 | 36 | 3.2% | -40.80 [-57.27, -24.33] |
| Polat 2003 (6) | 112.8 | 33.6 | 52 | 124.8 | 38.4 | 54 | 3.7% | -12.00 [-25.72, 1.72] |
| Yalcin 2022 | 80.1 | 10.3 | 28 | 84.5 | 10.7 | 22 | 5.2% | -4.40 [-10.28, 1.48] |
| **Subtotal (95% CI)** | | | **925** | | | **889** | **31.5%** | **-17.62 [-27.35, -7.88]** |
| Heterogeneity: Tau² = 153.28; Chi² = 56.40, df = 7 (P < 0.00001); I² = 88% | | | | | | | | |
| Test for overall effect: Z = 3.55 (P = 0.0004) | | | | | | | | |
| **3.2.5 30mg** | | | | | | | | |
| Roy 2008 | 64.1 | 2.86 | 82 | 72.8 | 2.86 | 82 | 5.7% | -8.70 [-9.58, -7.82] |
| **Subtotal (95% CI)** | | | **82** | | | **82** | **5.7%** | **-8.70 [-9.58, -7.82]** |
| Heterogeneity: Not applicable | | | | | | | | |
| Test for overall effect: Z = 19.48 (P < 0.00001) | | | | | | | | |
| **3.2.6 40mg** | | | | | | | | |
| Dutta 2000 | 70.4 | 10 | 44 | 103.4 | 17.1 | 36 | 5.1% | -33.00 [-39.32, -26.68] |
| **Subtotal (95% CI)** | | | **44** | | | **36** | **5.1%** | **-33.00 [-39.32, -26.68]** |
| Heterogeneity: Not applicable | | | | | | | | |
| Test for overall effect: Z = 10.24 (P < 0.00001) | | | | | | | | |
| **3.2.7 unspecified** | | | | | | | | |
| Kakar 2022 | 27.63 | 14.22 | 72 | 38.86 | 20.7 | 72 | 5.2% | -11.23 [-17.03, -5.43] |
| Patel 2009 | 64.4 | 37.8 | 248 | 62.2 | 33.5 | 247 | 5.1% | 2.20 [-4.09, 8.49] |
| **Subtotal (95% CI)** | | | **320** | | | **319** | **10.3%** | **-4.57 [-17.73, 8.59]** |
| Heterogeneity: Tau² = 80.65; Chi² = 9.46, df = 1 (P = 0.002); I² = 89% | | | | | | | | |
| Test for overall effect: Z = 0.68 (P = 0.50) | | | | | | | | |
| **3.2.8 dose by weight or age** | | | | | | | | |
| Al Sonboli 2003 (7) | 28.8 | 19.2 | 37 | 60 | 43.2 | 37 | 3.4% | -31.20 [-46.43, -15.97] |
| Bahl 2002 (8) | 33.6 | 57.6 | 404 | 40.8 | 60 | 200 | 4.4% | -7.20 [-17.23, 2.83] |
| Bahl 2002 (9) | 38.4 | 52.8 | 402 | 40.8 | 60 | 201 | 4.5% | -2.40 [-12.17, 7.37] |
| Bhatnagar 2004 (10) | 55.8 | 37 | 132 | 64.6 | 45.6 | 134 | 4.4% | -8.80 [-18.77, 1.17] |
| Boran 2005 (11) | 72.48 | 48 | 145 | 88.08 | 76.8 | 120 | 3.3% | -15.60 [-31.41, 0.21] |
| Crisinel 2015 (12) | 62 | 50.32 | 39 | 64.9 | 58.2 | 40 | 2.1% | -2.90 [-26.87, 21.07] |
| Dalgic 2011 (13) | 81.84 | 33.12 | 60 | 128.4 | 43.2 | 60 | 3.7% | -46.56 [-60.33, -32.79] |
| Patro 2010 (14) | 78.75 | 37 | 69 | 71.5 | 38.6 | 72 | 3.9% | 7.25 [-5.23, 19.73] |
| **Subtotal (95% CI)** | | | **1288** | | | **864** | **29.6%** | **-13.33 [-24.81, -1.84]** |
| Heterogeneity: Tau² = 223.88; Chi² = 45.05, df = 7 (P < 0.00001); I² = 84% | | | | | | | | |
| Test for overall effect: Z = 2.27 (P = 0.02) | | | | | | | | |
| **Total (95% CI)** | | | **3381** | | | **2868** | **100.0%** | **-13.27 [-17.66, -8.89]** |
| Heterogeneity: Tau² = 87.49; Chi² = 216.51, df = 23 (P < 0.00001); I² = 89% | | | | | | | | |
| Test for overall effect: Z = 5.93 (P < 0.00001) | | | | | | | | |
| Test for subgroup differences: Chi² = 104.88, df = 7 (P < 0.00001), I² = 93.3% | | | | | | | | |

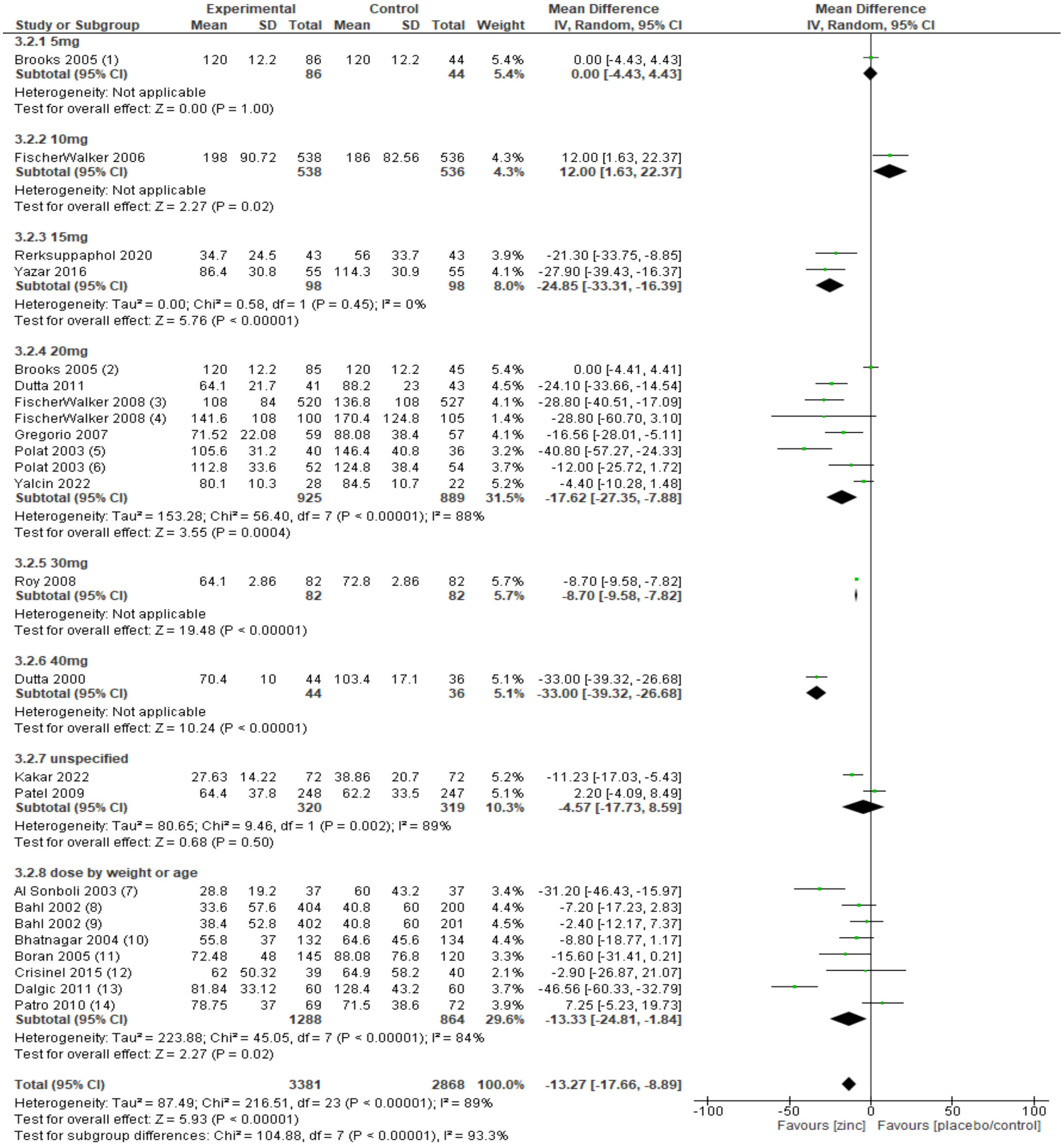


Footnotes
(1) 5mg
(2) 20mg
(3) 12-59 months
(4) <12 months
(5) low serum zinc
(6) normal serum zinc
(7) 3-6m: 22.5 mg, 7-60months: 45mg
(8) zinc syrup-12 to 35 months- 30 mg 6 to 11 months- 15 mg
(9) zinc-ORS: 12 to 35 months- 30 mg 6 to 11 months- 15 mg
(10) Participants <12 and >12 months were given 15 mg and 30 mg of zinc respectively
(11) 15 mg for 6-12 months and 30 mg for 12-60 month old
(12) 10 mg once a day (QD) in children <6 months and 20 mg QD for children ≥6 months.
(13) zinc-less than 6 months 10mg/day more than 6 months 20 mg/day
(14) 10 mg for <6 months and 20mg for >6 months

Figure 4C

## Outcome 4: Duration of diarrhea (subgroup analysis by duration of zinc supplementation)

| | Experimental | | | Control | | | | Mean Difference |
|---|---|---|---|---|---|---|---|---|
| **Study or Subgroup** | **Mean** | **SD** | **Total** | **Mean** | **SD** | **Total** | **Weight** | **IV, Random, 95% CI** |
| **8.2.1 <=5d** | | | | | | | | |
| Al Sonboli 2003 | 28.8 | 19.2 | 37 | 60 | 43.2 | 37 | 3.4% | -31.20 [-46.43, -15.97] |
| Dutta 2000 | 70.4 | 10 | 44 | 103.4 | 17.1 | 36 | 5.1% | -33.00 [-39.32, -26.68] |
| Yazar 2016 | 86.4 | 30.8 | 55 | 114.3 | 30.9 | 55 | 4.1% | -27.90 [-39.43, -16.37] |
| **Subtotal (95% CI)** | | | **136** | | | **128** | **12.6%** | **-31.75 [-36.96, -26.54]** |
| Heterogeneity: Tau² = 0.00; Chi² = 0.58, df = 2 (P = 0.75); I² = 0% | | | | | | | | |
| Test for overall effect: Z = 11.95 (P < 0.00001) | | | | | | | | |
| **8.2.2 <=10d** | | | | | | | | |
| Crisinel 2015 | 62 | 50.32 | 39 | 64.9 | 58.2 | 40 | 2.1% | -2.90 [-26.87, 21.07] |
| Patro 2010 | 78.75 | 37 | 69 | 71.5 | 38.6 | 72 | 3.9% | 7.25 [-5.23, 19.73] |
| Roy 2008 | 64.1 | 2.86 | 82 | 72.8 | 2.86 | 82 | 5.7% | -8.70 [-9.58, -7.82] |
| Yalcin 2022 | 80.1 | 10.3 | 28 | 84.5 | 10.7 | 22 | 5.2% | -4.40 [-10.28, 1.48] |
| **Subtotal (95% CI)** | | | **218** | | | **216** | **16.9%** | **-4.55 [-10.49, 1.39]** |
| Heterogeneity: Tau² = 19.37; Chi² = 8.40, df = 3 (P = 0.04); I² = 64% | | | | | | | | |
| Test for overall effect: Z = 1.50 (P = 0.13) | | | | | | | | |
| **8.2.3 14d** | | | | | | | | |
| Bahl 2002 (1) | 33.6 | 57.6 | 404 | 40.8 | 60 | 200 | 4.4% | -7.20 [-17.23, 2.83] |
| Bahl 2002 (2) | 38.4 | 52.8 | 402 | 40.8 | 60 | 201 | 4.5% | -2.40 [-12.17, 7.37] |
| Bhatnagar 2004 | 55.8 | 37 | 132 | 64.6 | 45.6 | 134 | 4.4% | -8.80 [-18.77, 1.17] |
| Boran 2005 | 72.48 | 48 | 145 | 88.08 | 76.8 | 120 | 3.3% | -15.60 [-31.41, 0.21] |
| Dutta 2011 | 64.1 | 21.7 | 41 | 88.2 | 23 | 43 | 4.5% | -24.10 [-33.66, -14.54] |
| FischerWalker 2006 | 198 | 90.72 | 538 | 186 | 82.56 | 536 | 4.3% | 12.00 [1.63, 22.37] |
| FischerWalker 2008 (3) | 141.6 | 108 | 100 | 170.4 | 124.8 | 105 | 1.4% | -28.80 [-60.70, 3.10] |
| FischerWalker 2008 (4) | 108 | 84 | 520 | 136.8 | 108 | 527 | 4.1% | -28.80 [-40.51, -17.09] |
| Gregorio 2007 | 71.52 | 22.08 | 59 | 88.08 | 38.4 | 57 | 4.1% | -16.56 [-28.01, -5.11] |
| Patel 2009 | 64.4 | 37.8 | 248 | 62.2 | 33.5 | 247 | 5.1% | 2.20 [-4.09, 8.49] |
| Polat 2003 (5) | 112.8 | 33.6 | 52 | 124.8 | 38.4 | 54 | 3.7% | -12.00 [-25.72, 1.72] |
| Polat 2003 (6) | 105.6 | 31.2 | 40 | 146.4 | 40.8 | 36 | 3.2% | -40.80 [-57.27, -24.33] |
| **Subtotal (95% CI)** | | | **2681** | | | **2260** | **46.9%** | **-12.86 [-20.98, -4.73]** |
| Heterogeneity: Tau² = 162.78; Chi² = 68.68, df = 11 (P < 0.00001); I² = 84% | | | | | | | | |
| Test for overall effect: Z = 3.10 (P = 0.002) | | | | | | | | |
| **8.2.4 unspecified** | | | | | | | | |
| Brooks 2005 (7) | 120 | 12.2 | 86 | 120 | 12.2 | 45 | 5.4% | 0.00 [-4.40, 4.40] |
| Brooks 2005 (8) | 120 | 12.2 | 85 | 120 | 12.2 | 44 | 5.4% | 0.00 [-4.44, 4.44] |
| Dalgic 2011 | 81.84 | 33.12 | 60 | 128.4 | 43.2 | 60 | 3.7% | -46.56 [-60.33, -32.79] |
| Kakar 2022 | 27.63 | 14.22 | 72 | 38.86 | 20.7 | 72 | 5.2% | -11.23 [-17.03, -5.43] |
| Rerksuppaphol 2020 | 34.7 | 24.5 | 43 | 56 | 33.7 | 43 | 3.9% | -21.30 [-33.75, -8.85] |
| **Subtotal (95% CI)** | | | **346** | | | **264** | **23.6%** | **-13.96 [-24.81, -3.10]** |
| Heterogeneity: Tau² = 133.89; Chi² = 55.82, df = 4 (P < 0.00001); I² = 93% | | | | | | | | |
| Test for overall effect: Z = 2.52 (P = 0.01) | | | | | | | | |
| **Total (95% CI)** | | | **3381** | | | **2868** | **100.0%** | **-13.27 [-17.66, -8.89]** |
| Heterogeneity: Tau² = 87.49; Chi² = 216.51, df = 23 (P < 0.00001); I² = 89% | | | | | | | | |
| Test for overall effect: Z = 5.93 (P < 0.00001) | | | | | | | | |
| Test for subgroup differences: Chi² = 48.55, df = 3 (P < 0.00001), I² = 93.8% | | | | | | | | |

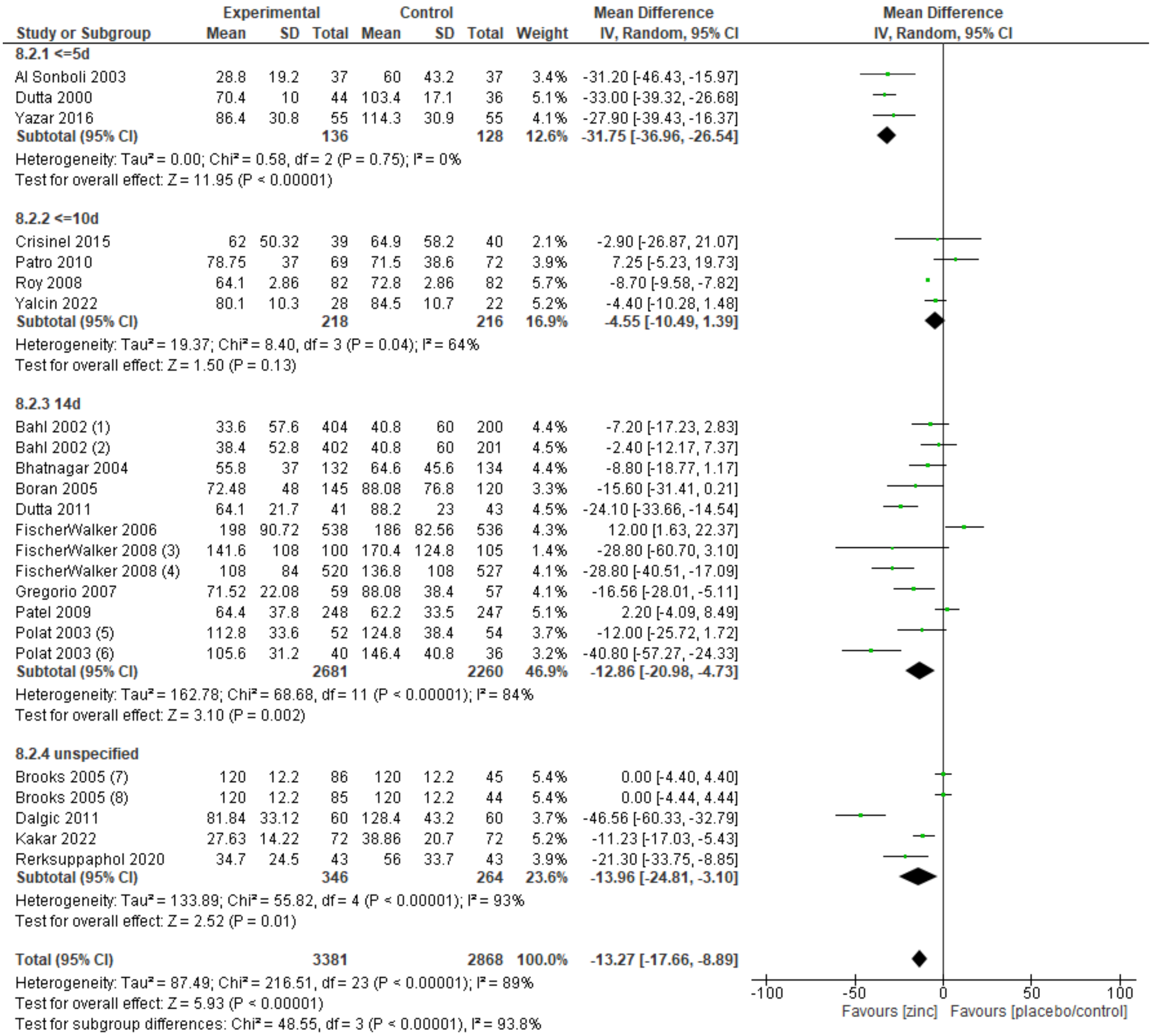


Footnotes
(1) zinc syrup
(2) zinc-ORS
(3) <12 months
(4) 12-59 months
(5) normal serum zinc
(6) low serum zinc
(7) 20mg
(8) 5mg

Figure 4D

## Outcome 4: Duration of diarrhea (subgroups by type of zinc formulation)

| Study or Subgroup | Experimental Mean | Experimental SD | Experimental Total | Control Mean | Control SD | Control Total | Weight | Mean Difference IV, Random, 95% CI |
|---|---|---|---|---|---|---|---|---|
| **4.2.1 zinc gluconate** | | | | | | | | |
| Bahl 2002 (1) | 38.4 | 52.8 | 402 | 40.8 | 60 | 201 | 4.5% | -2.40 [-12.17, 7.37] |
| Bahl 2002 (2) | 33.6 | 57.6 | 404 | 40.8 | 60 | 200 | 4.4% | -7.20 [-17.23, 2.83] |
| **Subtotal (95% CI)** | | | **806** | | | **401** | **8.9%** | **-4.74 [-11.74, 2.26]** |
| Heterogeneity: Tau² = 0.00; Chi² = 0.45, df = 1 (P = 0.50); I² = 0% | | | | | | | | |
| Test for overall effect: Z = 1.33 (P = 0.18) | | | | | | | | |
| **4.2.2 zinc sulfate** | | | | | | | | |
| Bhatnagar 2004 | 55.8 | 37 | 132 | 64.6 | 45.6 | 134 | 4.4% | -8.80 [-18.77, 1.17] |
| Boran 2005 | 72.48 | 48 | 145 | 88.08 | 76.8 | 120 | 3.3% | -15.60 [-31.41, 0.21] |
| Crisinel 2015 | 62 | 50.32 | 39 | 64.9 | 58.2 | 40 | 2.1% | -2.90 [-26.87, 21.07] |
| Dutta 2000 | 70.4 | 10 | 44 | 103.4 | 17.1 | 36 | 5.1% | -33.00 [-39.32, -26.68] |
| Gregorio 2007 | 71.52 | 22.08 | 59 | 88.08 | 38.4 | 57 | 4.1% | -16.56 [-28.01, -5.11] |
| Patro 2010 | 78.75 | 37 | 69 | 71.5 | 38.6 | 72 | 3.9% | 7.25 [-5.23, 19.73] |
| Polat 2003 (3) | 105.6 | 31.2 | 40 | 146.4 | 40.8 | 36 | 3.2% | -40.80 [-57.27, -24.33] |
| Polat 2003 (4) | 112.8 | 33.6 | 52 | 124.8 | 38.4 | 54 | 3.7% | -12.00 [-25.72, 1.72] |
| **Subtotal (95% CI)** | | | **580** | | | **549** | **29.8%** | **-15.70 [-27.12, -4.27]** |
| Heterogeneity: Tau² = 221.13; Chi² = 49.46, df = 7 (P < 0.00001); I² = 86% | | | | | | | | |
| Test for overall effect: Z = 2.69 (P = 0.007) | | | | | | | | |
| **4.2.3 zinc acetate** | | | | | | | | |
| Brooks 2005 (5) | 120 | 12.2 | 86 | 120 | 12.2 | 45 | 5.4% | 0.00 [-4.40, 4.40] |
| Brooks 2005 (6) | 120 | 12.2 | 85 | 120 | 12.2 | 44 | 5.4% | 0.00 [-4.44, 4.44] |
| Dalgic 2011 | 81.84 | 33.12 | 60 | 128.4 | 43.2 | 60 | 3.7% | -46.56 [-60.33, -32.79] |
| FischerWalker 2008 (7) | 108 | 84 | 520 | 136.8 | 108 | 527 | 4.1% | -28.80 [-40.51, -17.09] |
| FischerWalker 2008 (8) | 141.6 | 108 | 100 | 170.4 | 124.8 | 105 | 1.4% | -28.80 [-60.70, 3.10] |
| Roy 2008 | 64.1 | 2.86 | 82 | 72.8 | 2.86 | 82 | 5.7% | -8.70 [-9.58, -7.82] |
| **Subtotal (95% CI)** | | | **933** | | | **863** | **25.7%** | **-14.44 [-22.71, -6.17]** |
| Heterogeneity: Tau² = 78.45; Chi² = 70.89, df = 5 (P < 0.00001); I² = 93% | | | | | | | | |
| Test for overall effect: Z = 3.42 (P = 0.0006) | | | | | | | | |
| **4.2.4 zinc bisglycinate** | | | | | | | | |
| Rerksuppaphol 2020 | 34.7 | 24.5 | 43 | 56 | 33.7 | 43 | 3.9% | -21.30 [-33.75, -8.85] |
| **Subtotal (95% CI)** | | | **43** | | | **43** | **3.9%** | **-21.30 [-33.75, -8.85]** |
| Heterogeneity: Not applicable | | | | | | | | |
| Test for overall effect: Z = 3.35 (P = 0.0008) | | | | | | | | |
| **4.2.5 elemental zinc/unspecified** | | | | | | | | |
| Al Sonboli 2003 | 28.8 | 19.2 | 37 | 60 | 43.2 | 37 | 3.4% | -31.20 [-46.43, -15.97] |
| Dutta 2011 | 64.1 | 21.7 | 41 | 88.2 | 23 | 43 | 4.5% | -24.10 [-33.66, -14.54] |
| FischerWalker 2006 | 198 | 90.72 | 538 | 186 | 82.56 | 536 | 4.3% | 12.00 [1.63, 22.37] |
| Kakar 2022 | 27.63 | 14.22 | 72 | 38.86 | 20.7 | 72 | 5.2% | -11.23 [-17.03, -5.43] |
| Patel 2009 | 64.4 | 37.8 | 248 | 62.2 | 33.5 | 247 | 5.1% | 2.20 [-4.09, 8.49] |
| Yalcin 2022 | 80.1 | 10.3 | 28 | 84.5 | 10.7 | 22 | 5.2% | -4.40 [-10.28, 1.48] |
| Yazar 2016 | 86.4 | 30.8 | 55 | 114.3 | 30.9 | 55 | 4.1% | -27.90 [-39.43, -16.37] |
| **Subtotal (95% CI)** | | | **1019** | | | **1012** | **31.8%** | **-11.26 [-20.90, -1.63]** |
| Heterogeneity: Tau² = 145.85; Chi² = 58.24, df = 6 (P < 0.00001); I² = 90% | | | | | | | | |
| Test for overall effect: Z = 2.29 (P = 0.02) | | | | | | | | |
| **Total (95% CI)** | | | **3381** | | | **2868** | **100.0%** | **-13.27 [-17.66, -8.89]** |
| Heterogeneity: Tau² = 87.49; Chi² = 216.51, df = 23 (P < 0.00001); I² = 89% | | | | | | | | |
| Test for overall effect: Z = 5.93 (P < 0.00001) | | | | | | | | |
| Test for subgroup differences: Chi² = 6.97, df = 4 (P = 0.14), I² = 42.6% | | | | | | | | |

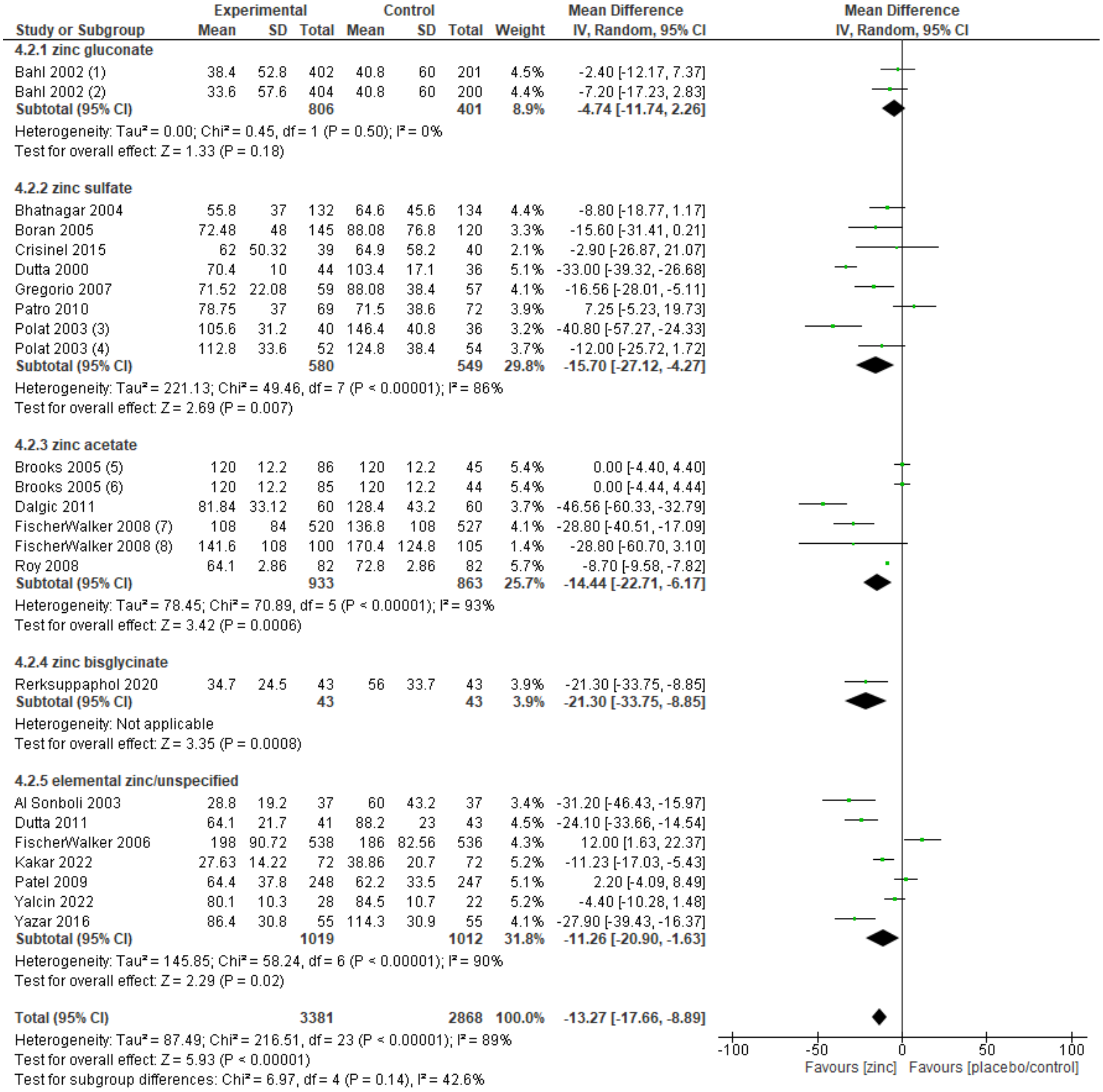


Footnotes

(1) zinc-ORS

(2) zinc syrup

(3) low serum zinc

(4) normal serum zinc

(5) 20mg

(6) 5mg

(7) 12-59 months

(8) <12 months

Figure 4E

Outcome 4: Duration of diarrhea (subgroups based on World Bank Income Classification)

| Study or Subgroup | Experimental Mean | Experimental SD | Experimental Total | Control Mean | Control SD | Control Total | Weight | Mean Difference IV, Random, 95% CI |
|---|---|---|---|---|---|---|---|---|
| **2.2.1 LMIC** | | | | | | | | |
| Bahl 2002 (1) | 33.6 | 57.6 | 404 | 40.8 | 60 | 200 | 4.4% | -7.20 [-17.23, 2.83] |
| Bahl 2002 (2) | 38.4 | 52.8 | 402 | 40.8 | 60 | 201 | 4.5% | -2.40 [-12.17, 7.37] |
| Bhatnagar 2004 | 55.8 | 37 | 132 | 64.6 | 45.6 | 134 | 4.4% | -8.80 [-18.77, 1.17] |
| Brooks 2005 (3) | 120 | 12.2 | 86 | 120 | 12.2 | 45 | 5.4% | 0.00 [-4.40, 4.40] |
| Brooks 2005 (4) | 120 | 12.2 | 85 | 120 | 12.2 | 44 | 5.4% | 0.00 [-4.44, 4.44] |
| Dutta 2000 | 70.4 | 10 | 44 | 103.4 | 17.1 | 36 | 5.1% | -33.00 [-39.32, -26.68] |
| Dutta 2011 | 64.1 | 21.7 | 41 | 88.2 | 23 | 43 | 4.5% | -24.10 [-33.66, -14.54] |
| FischerWalker 2006 | 198 | 90.72 | 538 | 186 | 82.56 | 536 | 4.3% | 12.00 [1.63, 22.37] |
| FischerWalker 2008 (5) | 141.6 | 108 | 100 | 170.4 | 124.8 | 105 | 1.4% | -28.80 [-60.70, 3.10] |
| FischerWalker 2008 (6) | 108 | 84 | 520 | 136.8 | 108 | 527 | 4.1% | -28.80 [-40.51, -17.09] |
| Gregorio 2007 | 71.52 | 22.08 | 59 | 88.08 | 38.4 | 57 | 4.1% | -16.56 [-28.01, -5.11] |
| Kakar 2022 | 27.63 | 14.22 | 72 | 38.86 | 20.7 | 72 | 5.2% | -11.23 [-17.03, -5.43] |
| Patel 2009 | 64.4 | 37.8 | 248 | 62.2 | 33.5 | 247 | 5.1% | 2.20 [-4.09, 8.49] |
| Polat 2003 (7) | 105.6 | 31.2 | 40 | 146.4 | 40.8 | 36 | 3.2% | -40.80 [-57.27, -24.33] |
| Polat 2003 (8) | 112.8 | 33.6 | 52 | 124.8 | 38.4 | 54 | 3.7% | -12.00 [-25.72, 1.72] |
| Roy 2008 | 64.1 | 2.86 | 82 | 72.8 | 2.86 | 82 | 5.7% | -8.70 [-9.58, -7.82] |
| **Subtotal (95% CI)** | | | **2905** | | | **2419** | **70.5%** | **-11.29 [-16.27, -6.30]** |
| Heterogeneity: Tau² = 77.90; Chi² = 155.13, df = 15 (P < 0.00001); I² = 90% | | | | | | | | |
| Test for overall effect: Z = 4.44 (P < 0.00001) | | | | | | | | |
| **2.2.3 HIC** | | | | | | | | |
| Al Sonboli 2003 | 28.8 | 19.2 | 37 | 60 | 43.2 | 37 | 3.4% | -31.20 [-46.43, -15.97] |
| Boran 2005 | 72.48 | 48 | 145 | 88.08 | 76.8 | 120 | 3.3% | -15.60 [-31.41, 0.21] |
| Crisinel 2015 | 62 | 50.32 | 39 | 64.9 | 58.2 | 40 | 2.1% | -2.90 [-26.87, 21.07] |
| Dalgic 2011 | 81.84 | 33.12 | 60 | 128.4 | 43.2 | 60 | 3.7% | -46.56 [-60.33, -32.79] |
| Patro 2010 | 78.75 | 37 | 69 | 71.5 | 38.6 | 72 | 3.9% | 7.25 [-5.23, 19.73] |
| Rerksuppaphol 2020 | 34.7 | 24.5 | 43 | 56 | 33.7 | 43 | 3.9% | -21.30 [-33.75, -8.85] |
| Yalcin 2022 | 80.1 | 10.3 | 28 | 84.5 | 10.7 | 22 | 5.2% | -4.40 [-10.28, 1.48] |
| Yazar 2016 | 86.4 | 30.8 | 55 | 114.3 | 30.9 | 55 | 4.1% | -27.90 [-39.43, -16.37] |
| **Subtotal (95% CI)** | | | **476** | | | **449** | **29.5%** | **-17.97 [-30.21, -5.73]** |
| Heterogeneity: Tau² = 259.94; Chi² = 55.47, df = 7 (P < 0.00001); I² = 87% | | | | | | | | |
| Test for overall effect: Z = 2.88 (P = 0.004) | | | | | | | | |
| **Total (95% CI)** | | | **3381** | | | **2868** | **100.0%** | **-13.27 [-17.66, -8.89]** |
| Heterogeneity: Tau² = 87.49; Chi² = 216.51, df = 23 (P < 0.00001); I² = 89% | | | | | | | | |
| Test for overall effect: Z = 5.93 (P < 0.00001) | | | | | | | | |
| Test for subgroup differences: Chi² = 0.98, df = 1 (P = 0.32), I² = 0% | | | | | | | | |

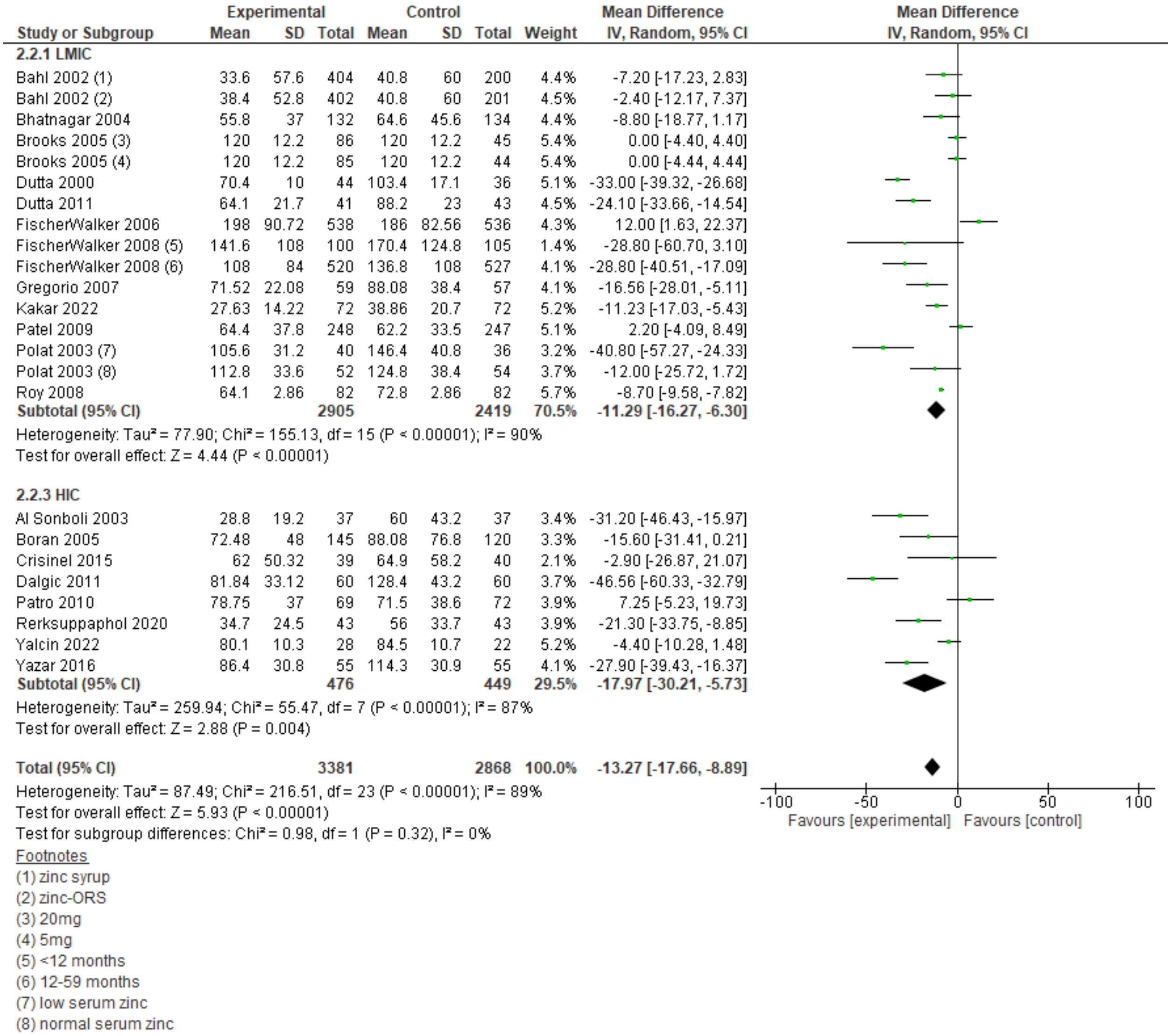


Footnotes
(1) zinc syrup
(2) zinc-ORS
(3) 20mg
(4) 5mg
(5) <12 months
(6) 12-59 months
(7) low serum zinc
(8) normal serum zinc

Figure 4F

Outcome 4: Duration of diarrhea (subgroups based on form of zinc given)

| | Experimental | | | Control | | | | Mean Difference |
|---|---|---|---|---|---|---|---|---|
| **Study or Subgroup** | **Mean** | **SD** | **Total** | **Mean** | **SD** | **Total** | **Weight** | **IV, Random, 95% CI** |
| **5.2.1 syrup** | | | | | | | | |
| Bahl 2002 (1) | 33.6 | 57.6 | 404 | 40.8 | 60 | 200 | 4.4% | -7.20 [-17.23, 2.83] |
| Bhatnagar 2004 | 55.8 | 37 | 132 | 64.6 | 45.6 | 134 | 4.4% | -8.80 [-18.77, 1.17] |
| Boran 2005 | 72.48 | 48 | 145 | 88.08 | 76.8 | 120 | 3.3% | -15.60 [-31.41, 0.21] |
| Brooks 2005 (2) | 120 | 12.2 | 86 | 120 | 12.2 | 44 | 5.4% | 0.00 [-4.43, 4.43] |
| Brooks 2005 (3) | 120 | 12.2 | 85 | 120 | 12.2 | 45 | 5.4% | 0.00 [-4.41, 4.41] |
| Dalgic 2011 | 81.84 | 33.12 | 60 | 128.4 | 43.2 | 60 | 3.7% | -46.56 [-60.33, -32.79] |
| Dutta 2000 | 70.4 | 10 | 44 | 103.4 | 17.1 | 36 | 5.1% | -33.00 [-39.32, -26.68] |
| Patro 2010 | 78.75 | 37 | 69 | 71.5 | 38.6 | 72 | 3.9% | 7.25 [-5.23, 19.73] |
| Polat 2003 (4) | 105.6 | 31.2 | 40 | 146.4 | 40.8 | 36 | 3.2% | -40.80 [-57.27, -24.33] |
| Polat 2003 (5) | 112.8 | 33.6 | 52 | 124.8 | 38.4 | 54 | 3.7% | -12.00 [-25.72, 1.72] |
| Roy 2008 | 64.1 | 2.86 | 82 | 72.8 | 2.86 | 82 | 5.7% | -8.70 [-9.58, -7.82] |
| Yalcin 2022 | 80.1 | 10.3 | 28 | 84.5 | 10.7 | 22 | 5.2% | -4.40 [-10.28, 1.48] |
| Yazar 2016 | 86.4 | 30.8 | 55 | 114.3 | 30.9 | 55 | 4.1% | -27.90 [-39.43, -16.37] |
| **Subtotal (95% CI)** | | | **1282** | | | **960** | **57.4%** | **-13.87 [-19.82, -7.92]** |
| Heterogeneity: Tau² = 93.77; Chi² = 150.22, df = 12 (P < 0.00001); I² = 92% | | | | | | | | |
| Test for overall effect: Z = 4.57 (P < 0.00001) | | | | | | | | |
| **5.2.2 dispersible tablet** | | | | | | | | |
| Al Sonboli 2003 | 28.8 | 19.2 | 37 | 60 | 43.2 | 37 | 3.4% | -31.20 [-46.43, -15.97] |
| Crisinel 2015 | 62 | 50.32 | 39 | 64.9 | 58.2 | 40 | 2.1% | -2.90 [-26.87, 21.07] |
| FischerWalker 2006 | 198 | 90.72 | 538 | 186 | 82.56 | 536 | 4.3% | 12.00 [1.63, 22.37] |
| Gregorio 2007 | 71.52 | 22.08 | 59 | 88.08 | 38.4 | 57 | 4.1% | -16.56 [-28.01, -5.11] |
| **Subtotal (95% CI)** | | | **673** | | | **670** | **13.9%** | **-9.63 [-30.02, 10.77]** |
| Heterogeneity: Tau² = 369.97; Chi² = 25.36, df = 3 (P < 0.0001); I² = 88% | | | | | | | | |
| Test for overall effect: Z = 0.93 (P = 0.35) | | | | | | | | |
| **5.2.3 powder sachet** | | | | | | | | |
| Rerksuppaphol 2020 | 34.7 | 24.5 | 43 | 56 | 33.7 | 43 | 3.9% | -21.30 [-33.75, -8.85] |
| **Subtotal (95% CI)** | | | **43** | | | **43** | **3.9%** | **-21.30 [-33.75, -8.85]** |
| Heterogeneity: Not applicable | | | | | | | | |
| Test for overall effect: Z = 3.35 (P = 0.0008) | | | | | | | | |
| **5.2.4 ORS** | | | | | | | | |
| Bahl 2002 (6) | 38.4 | 52.8 | 402 | 40.8 | 60 | 201 | 4.5% | -2.40 [-12.17, 7.37] |
| **Subtotal (95% CI)** | | | **402** | | | **201** | **4.5%** | **-2.40 [-12.17, 7.37]** |
| Heterogeneity: Not applicable | | | | | | | | |
| Test for overall effect: Z = 0.48 (P = 0.63) | | | | | | | | |
| **5.2.5 unspecified** | | | | | | | | |
| Dutta 2011 | 64.1 | 21.7 | 41 | 88.2 | 23 | 43 | 4.5% | -24.10 [-33.66, -14.54] |
| FischerWalker 2008 (7) | 141.6 | 108 | 100 | 170.4 | 124.8 | 105 | 1.4% | -28.80 [-60.70, 3.10] |
| FischerWalker 2008 (8) | 108 | 84 | 520 | 136.8 | 108 | 527 | 4.1% | -28.80 [-40.51, -17.09] |
| Kakar 2022 | 27.63 | 14.22 | 72 | 38.86 | 20.7 | 72 | 5.2% | -11.23 [-17.03, -5.43] |
| Patel 2009 | 64.4 | 37.8 | 248 | 62.2 | 33.5 | 247 | 5.1% | 2.20 [-4.09, 8.49] |
| **Subtotal (95% CI)** | | | **981** | | | **994** | **20.3%** | **-16.13 [-28.36, -3.90]** |
| Heterogeneity: Tau² = 152.76; Chi² = 34.13, df = 4 (P < 0.00001); I² = 88% | | | | | | | | |
| Test for overall effect: Z = 2.58 (P = 0.010) | | | | | | | | |
| **Total (95% CI)** | | | **3381** | | | **2868** | **100.0%** | **-13.27 [-17.66, -8.89]** |
| Heterogeneity: Tau² = 87.49; Chi² = 216.51, df = 23 (P < 0.00001); I² = 89% | | | | | | | | |
| Test for overall effect: Z = 5.93 (P < 0.00001) | | | | | | | | |
| Test for subgroup differences: Chi² = 6.64, df = 4 (P = 0.16), I² = 39.7% | | | | | | | | |

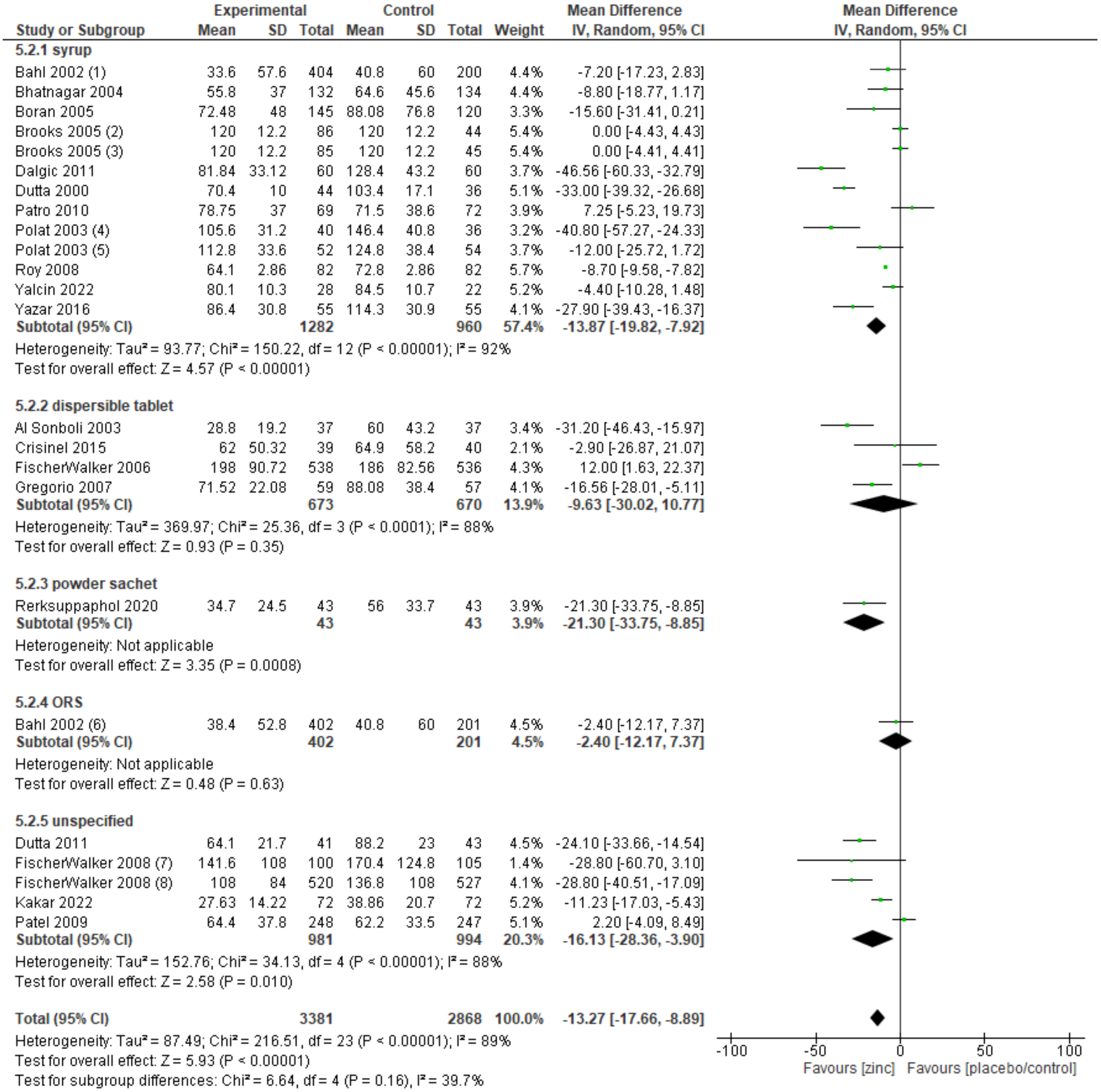


Footnotes
(1) zinc syrup
(2) 5mg
(3) 20mg
(4) low serum zinc
(5) normal serum zinc
(6) zinc-ORS
(7) <12 months
(8) 12-59 months

Figure 4G

Outcome 4: Duration of diarrhea (subgroups based on age)

| | Experimental | | | Control | | | | Mean Difference |
|---|---|---|---|---|---|---|---|---|
| **Study or Subgroup** | **Mean** | **SD** | **Total** | **Mean** | **SD** | **Total** | **Weight** | **IV, Random, 95% CI** |
| **6.2.1 <6 months** | | | | | | | | |
| Brooks 2005 (1) | 120 | 12.2 | 86 | 120 | 12.2 | 45 | 5.4% | 0.00 [-4.40, 4.40] |
| Brooks 2005 (2) | 120 | 12.2 | 85 | 120 | 12.2 | 44 | 5.4% | 0.00 [-4.44, 4.44] |
| FischerWalker 2006 | 198 | 90.72 | 538 | 186 | 82.56 | 536 | 4.3% | 12.00 [1.63, 22.37] |
| **Subtotal (95% CI)** | | | **709** | | | **625** | **15.1%** | **2.05 [-3.01, 7.12]** |
| Heterogeneity: Tau² = 11.04; Chi² = 4.71, df = 2 (P = 0.09); I² = 58% | | | | | | | | |
| Test for overall effect: Z = 0.79 (P = 0.43) | | | | | | | | |
| **6.2.2 >6 months** | | | | | | | | |
| Bahl 2002 (3) | 38.4 | 52.8 | 402 | 40.8 | 60 | 201 | 4.5% | -2.40 [-12.17, 7.37] |
| Bahl 2002 (4) | 33.6 | 57.6 | 404 | 40.8 | 60 | 200 | 4.4% | -7.20 [-17.23, 2.83] |
| Boran 2005 | 72.48 | 48 | 145 | 88.08 | 76.8 | 120 | 3.3% | -15.60 [-31.41, 0.21] |
| Dutta 2011 (5) | 64.1 | 21.7 | 41 | 88.2 | 23 | 43 | 4.5% | -24.10 [-33.66, -14.54] |
| Patel 2009 | 64.4 | 37.8 | 248 | 62.2 | 33.5 | 247 | 5.1% | 2.20 [-4.09, 8.49] |
| Rerksuppaphol 2020 | 34.7 | 24.5 | 43 | 56 | 33.7 | 43 | 3.9% | -21.30 [-33.75, -8.85] |
| Yalcin 2022 (6) | 80.1 | 10.3 | 28 | 84.5 | 10.7 | 22 | 5.2% | -4.40 [-10.28, 1.48] |
| Yazar 2016 | 86.4 | 30.8 | 55 | 114.3 | 30.9 | 55 | 4.1% | -27.90 [-39.43, -16.37] |
| **Subtotal (95% CI)** | | | **1366** | | | **931** | **34.9%** | **-11.86 [-19.75, -3.97]** |
| Heterogeneity: Tau² = 102.37; Chi² = 40.43, df = 7 (P < 0.00001); I² = 83% | | | | | | | | |
| Test for overall effect: Z = 2.95 (P = 0.003) | | | | | | | | |
| **6.2.3 <6 and >6 months** | | | | | | | | |
| Al Sonboli 2003 | 28.8 | 19.2 | 37 | 60 | 43.2 | 37 | 3.4% | -31.20 [-46.43, -15.97] |
| Bhatnagar 2004 | 55.8 | 37 | 132 | 64.6 | 45.6 | 134 | 4.4% | -8.80 [-18.77, 1.17] |
| Crisinel 2015 | 62 | 50.32 | 39 | 64.9 | 58.2 | 40 | 2.1% | -2.90 [-26.87, 21.07] |
| Dalgic 2011 (7) | 81.84 | 33.12 | 60 | 128.4 | 43.2 | 60 | 3.7% | -46.56 [-60.33, -32.79] |
| Dutta 2000 | 70.4 | 10 | 44 | 103.4 | 17.1 | 36 | 5.1% | -33.00 [-39.32, -26.68] |
| FischerWalker 2008 (8) | 108 | 84 | 520 | 136.8 | 108 | 527 | 4.1% | -28.80 [-40.51, -17.09] |
| FischerWalker 2008 (9) | 141.6 | 108 | 100 | 170.4 | 124.8 | 105 | 1.4% | -28.80 [-60.70, 3.10] |
| Gregorio 2007 | 71.52 | 22.08 | 59 | 88.08 | 38.4 | 57 | 4.1% | -16.56 [-28.01, -5.11] |
| Kakar 2022 | 27.63 | 14.22 | 72 | 38.86 | 20.7 | 72 | 5.2% | -11.23 [-17.03, -5.43] |
| Patro 2010 | 78.75 | 37 | 69 | 71.5 | 38.6 | 72 | 3.9% | 7.25 [-5.23, 19.73] |
| Polat 2003 (10) | 105.6 | 31.2 | 40 | 146.4 | 40.8 | 36 | 3.2% | -40.80 [-57.27, -24.33] |
| Polat 2003 (11) | 112.8 | 33.6 | 52 | 124.8 | 38.4 | 54 | 3.7% | -12.00 [-25.72, 1.72] |
| Roy 2008 | 64.1 | 2.86 | 82 | 72.8 | 2.86 | 82 | 5.7% | -8.70 [-9.58, -7.82] |
| **Subtotal (95% CI)** | | | **1306** | | | **1312** | **49.9%** | **-19.64 [-27.18, -12.11]** |
| Heterogeneity: Tau² = 145.47; Chi² = 126.79, df = 12 (P < 0.00001); I² = 91% | | | | | | | | |
| Test for overall effect: Z = 5.11 (P < 0.00001) | | | | | | | | |
| **Total (95% CI)** | | | **3381** | | | **2868** | **100.0%** | **-13.27 [-17.66, -8.89]** |
| Heterogeneity: Tau² = 87.49; Chi² = 216.51, df = 23 (P < 0.00001); I² = 89% | | | | | | | | |
| Test for overall effect: Z = 5.93 (P < 0.00001) | | | | | | | | |
| Test for subgroup differences: Chi² = 24.40, df = 2 (P < 0.00001), I² = 91.8% | | | | | | | | |

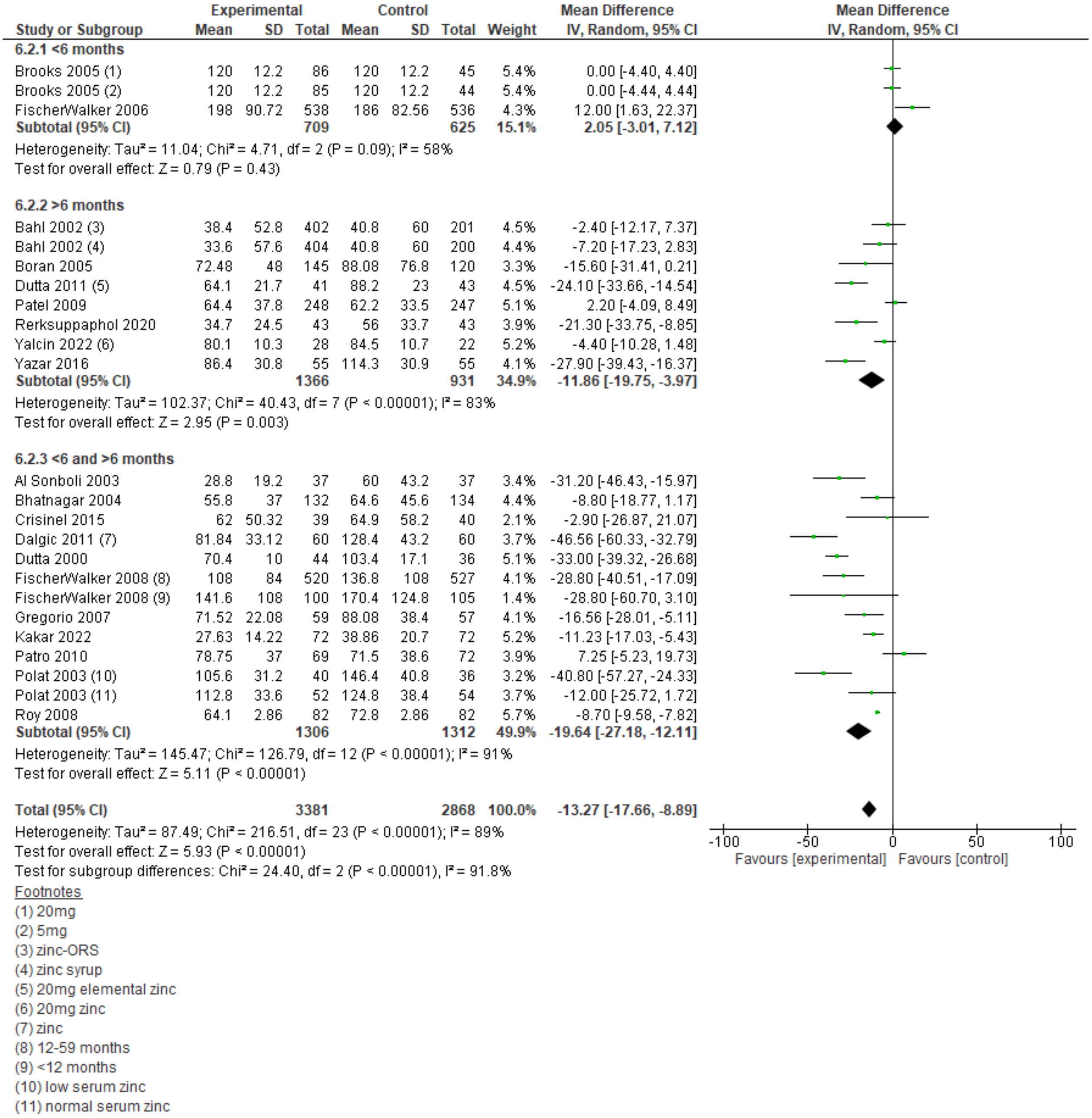


Footnotes
(1) 20mg
(2) 5mg
(3) zinc-ORS
(4) zinc syrup
(5) 20mg elemental zinc
(6) 20mg zinc
(7) zinc
(8) 12-59 months
(9) <12 months
(10) low serum zinc
(11) normal serum zinc

Figure 4H

Outcome 4: Vomiting (subgroups based on study setting)

| | Experimental | | | Control | | | | Mean Difference |
|---|---|---|---|---|---|---|---|---|
| **Study or Subgroup** | **Mean** | **SD** | **Total** | **Mean** | **SD** | **Total** | **Weight** | **IV, Random, 95% CI** |
| **7.2.1 inpatient** | | | | | | | | |
| Al Sonboli 2003 | 28.8 | 19.2 | 37 | 60 | 43.2 | 37 | 3.4% | -31.20 [-46.43, -15.97] |
| Bhatnagar 2004 | 55.8 | 37 | 132 | 64.6 | 45.6 | 134 | 4.4% | -8.80 [-18.77, 1.17] |
| Brooks 2005 (1) | 120 | 12.2 | 86 | 120 | 12.2 | 45 | 5.4% | 0.00 [-4.40, 4.40] |
| Brooks 2005 (2) | 120 | 12.2 | 85 | 120 | 12.2 | 44 | 5.4% | 0.00 [-4.44, 4.44] |
| Dalgic 2011 | 81.84 | 33.12 | 60 | 128.4 | 43.2 | 60 | 3.7% | -46.56 [-60.33, -32.79] |
| Dutta 2000 | 70.4 | 10 | 44 | 103.4 | 17.1 | 36 | 5.1% | -33.00 [-39.32, -26.68] |
| Dutta 2011 | 64.1 | 21.7 | 41 | 88.2 | 23 | 43 | 4.5% | -24.10 [-33.66, -14.54] |
| Rerksuppaphol 2020 | 34.7 | 24.5 | 43 | 56 | 33.7 | 43 | 3.9% | -21.30 [-33.75, -8.85] |
| Roy 2008 | 64.1 | 2.86 | 82 | 72.8 | 2.86 | 82 | 5.7% | -8.70 [-9.58, -7.82] |
| **Subtotal (95% CI)** | | | **610** | | | **524** | **41.5%** | **-17.77 [-25.27, -10.27]** |
| Heterogeneity: Tau² = 110.23; Chi² = 137.69, df = 8 (P < 0.00001); I² = 94% | | | | | | | | |
| Test for overall effect: Z = 4.64 (P < 0.00001) | | | | | | | | |
| **7.2.2 outpatient** | | | | | | | | |
| Bahl 2002 (3) | 33.6 | 57.6 | 404 | 40.8 | 60 | 200 | 4.4% | -7.20 [-17.23, 2.83] |
| Bahl 2002 (4) | 38.4 | 52.8 | 402 | 40.8 | 60 | 201 | 4.5% | -2.40 [-12.17, 7.37] |
| Boran 2005 | 72.48 | 48 | 145 | 88.08 | 76.8 | 120 | 3.3% | -15.60 [-31.41, 0.21] |
| FischerWalker 2006 | 198 | 90.72 | 538 | 186 | 82.56 | 536 | 4.3% | 12.00 [1.63, 22.37] |
| FischerWalker 2008 (5) | 141.6 | 108 | 100 | 170.4 | 124.8 | 105 | 1.4% | -28.80 [-60.70, 3.10] |
| FischerWalker 2008 (6) | 108 | 84 | 520 | 136.8 | 108 | 527 | 4.1% | -28.80 [-40.51, -17.09] |
| Gregorio 2007 | 71.52 | 22.08 | 59 | 88.08 | 38.4 | 57 | 4.1% | -16.56 [-28.01, -5.11] |
| Polat 2003 (7) | 112.8 | 33.6 | 52 | 124.8 | 38.4 | 54 | 3.7% | -12.00 [-25.72, 1.72] |
| Polat 2003 (8) | 105.6 | 31.2 | 40 | 146.4 | 40.8 | 36 | 3.2% | -40.80 [-57.27, -24.33] |
| Yalcin 2022 | 80.1 | 10.3 | 28 | 84.5 | 10.7 | 22 | 5.2% | -4.40 [-10.28, 1.48] |
| Yazar 2016 | 86.4 | 30.8 | 55 | 114.3 | 30.9 | 55 | 4.1% | -27.90 [-39.43, -16.37] |
| **Subtotal (95% CI)** | | | **2343** | | | **1913** | **42.2%** | **-14.26 [-22.71, -5.81]** |
| Heterogeneity: Tau² = 158.37; Chi² = 59.14, df = 10 (P < 0.00001); I² = 83% | | | | | | | | |
| Test for overall effect: Z = 3.31 (P = 0.0009) | | | | | | | | |
| **7.2.3 inpatient and outpatient** | | | | | | | | |
| Crisinel 2015 | 62 | 50.32 | 39 | 64.9 | 58.2 | 40 | 2.1% | -2.90 [-26.87, 21.07] |
| Patel 2009 | 64.4 | 37.8 | 248 | 62.2 | 33.5 | 247 | 5.1% | 2.20 [-4.09, 8.49] |
| Patro 2010 | 78.75 | 37 | 69 | 71.5 | 38.6 | 72 | 3.9% | 7.25 [-5.23, 19.73] |
| **Subtotal (95% CI)** | | | **356** | | | **359** | **11.1%** | **2.90 [-2.57, 8.37]** |
| Heterogeneity: Tau² = 0.00; Chi² = 0.74, df = 2 (P = 0.69); I² = 0% | | | | | | | | |
| Test for overall effect: Z = 1.04 (P = 0.30) | | | | | | | | |
| **7.2.4 unspecified** | | | | | | | | |
| Kakar 2022 | 27.63 | 14.22 | 72 | 38.86 | 20.7 | 72 | 5.2% | -11.23 [-17.03, -5.43] |
| **Subtotal (95% CI)** | | | **72** | | | **72** | **5.2%** | **-11.23 [-17.03, -5.43]** |
| Heterogeneity: Not applicable | | | | | | | | |
| Test for overall effect: Z = 3.79 (P = 0.0001) | | | | | | | | |
| **Total (95% CI)** | | | **3381** | | | **2868** | **100.0%** | **-13.27 [-17.66, -8.89]** |
| Heterogeneity: Tau² = 87.49; Chi² = 216.51, df = 23 (P < 0.00001); I² = 89% | | | | | | | | |
| Test for overall effect: Z = 5.93 (P < 0.00001) | | | | | | | | |
| Test for subgroup differences: Chi² = 25.08, df = 3 (P < 0.0001), I² = 88.0% | | | | | | | | |

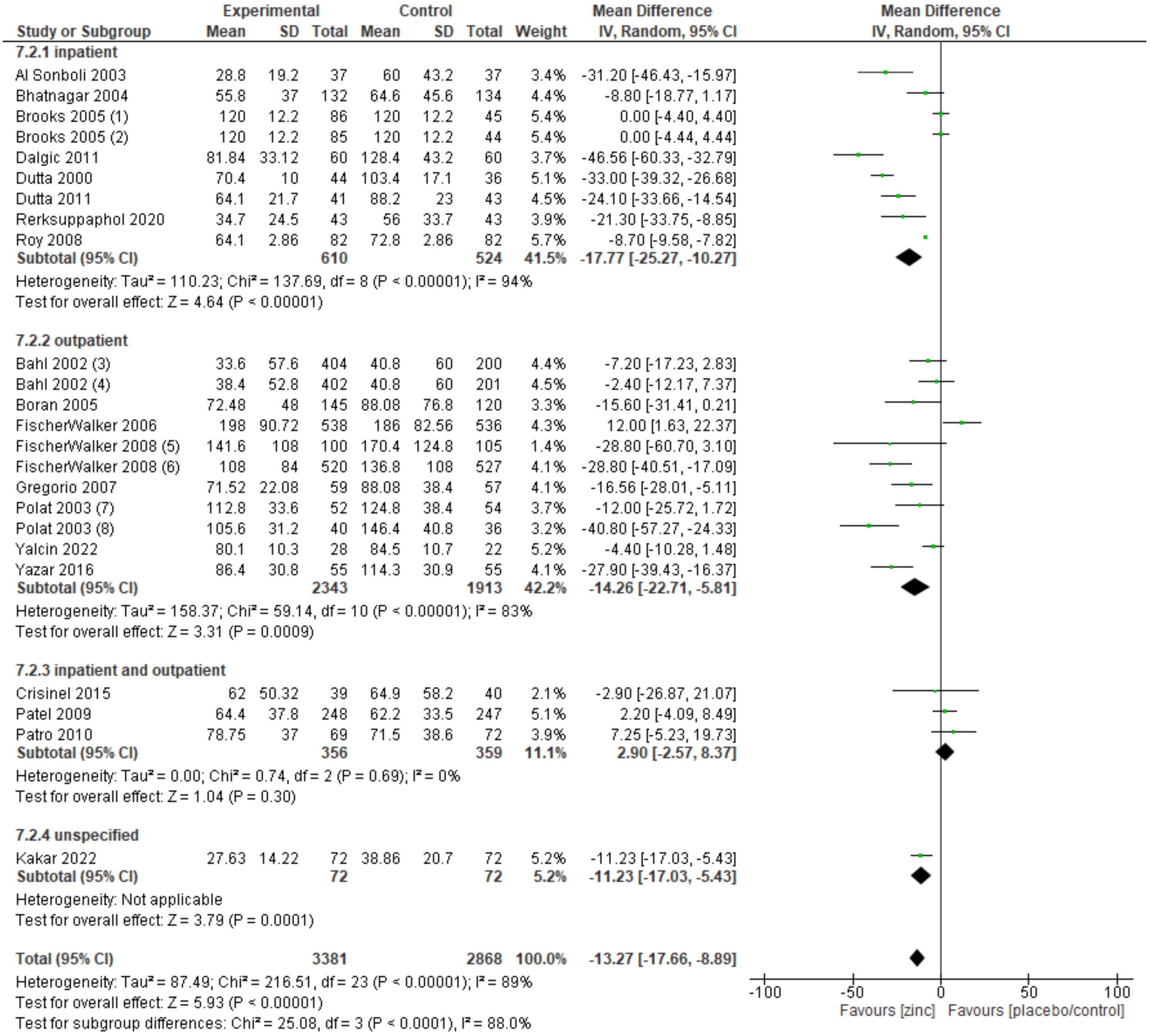


Footnotes
(1) 20mg
(2) 5mg
(3) zinc syrup
(4) zinc-ORS
(5) <12 months
(6) 12-59 months
(7) normal serum zinc
(8) low serum zinc

Figure 4I

Outcome 4: Duration of diarrhea (sensitivity analysis)

| | Experimental | | | Control | | | | Mean Difference |
|---|---|---|---|---|---|---|---|---|
| Study or Subgroup | Mean | SD | Total | Mean | SD | Total | Weight | IV, Random, 95% CI |
| Al Sonboli 2003 | 28.8 | 19.2 | 37 | 60 | 43.2 | 37 | 6.3% | -31.20 [-46.43, -15.97] |
| Bahl 2002 (1) | 38.4 | 52.8 | 402 | 40.8 | 60 | 201 | 8.4% | -2.40 [-12.17, 7.37] |
| Bahl 2002 (2) | 33.6 | 57.6 | 404 | 40.8 | 60 | 200 | 8.3% | -7.20 [-17.23, 2.83] |
| Bhatnagar 2004 | 55.8 | 37 | 132 | 64.6 | 45.6 | 134 | 8.3% | -8.80 [-18.77, 1.17] |
| Brooks 2005 (3) | 120 | 12.2 | 86 | 120 | 12.2 | 45 | 10.2% | 0.00 [-4.40, 4.40] |
| Brooks 2005 (4) | 120 | 12.2 | 85 | 120 | 12.2 | 44 | 10.2% | 0.00 [-4.44, 4.44] |
| Crisinel 2015 | 62 | 50.32 | 39 | 64.9 | 58.2 | 40 | 3.9% | -2.90 [-26.87, 21.07] |
| Dutta 2000 | 70.4 | 10 | 44 | 103.4 | 17.1 | 36 | 9.6% | -33.00 [-39.32, -26.68] |
| Dutta 2011 | 64.1 | 21.7 | 41 | 88.2 | 23 | 43 | 8.5% | -24.10 [-33.66, -14.54] |
| FischerWalker 2006 | 198 | 90.72 | 538 | 186 | 82.56 | 536 | 8.1% | 12.00 [1.63, 22.37] |
| Rerksuppaphol 2020 | 34.7 | 24.5 | 43 | 56 | 33.7 | 43 | 7.3% | -21.30 [-33.75, -8.85] |
| Roy 2008 | 64.1 | 2.86 | 82 | 72.8 | 2.86 | 82 | 10.8% | -8.70 [-9.58, -7.82] |
| **Total (95% CI)** | | | **1933** | | | **1441** | **100.0%** | **-10.36 [-16.30, -4.42]** |

Heterogeneity: Tau² = 84.85; Chi² = 125.94, df = 11 (P < 0.00001); I² = 91%

Test for overall effect: Z = 3.42 (P = 0.0006)

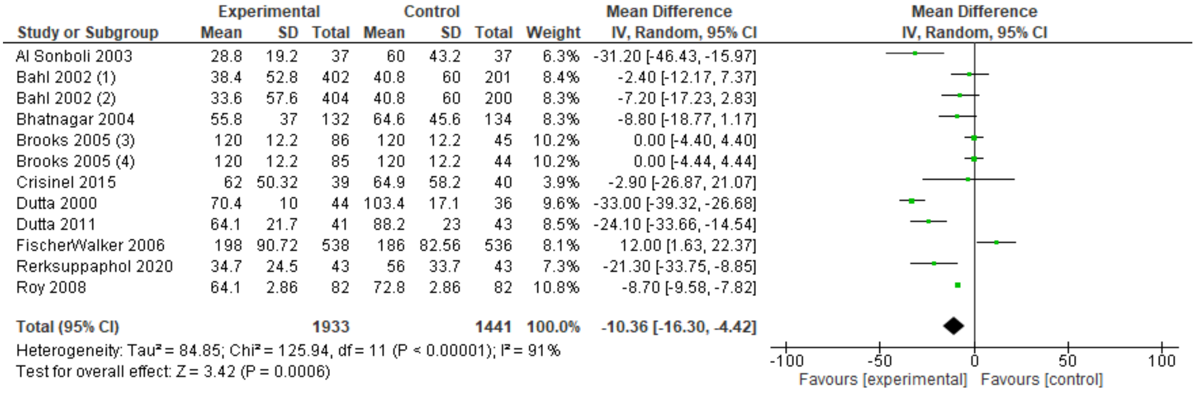


Footnotes
(1) zinc-ORS
(2) zinc syrup
(3) 20mg
(4) 5mg

**Zinc vs no zinc in persistent diarrhea**

Figure 5

Outcome1- Recovery from diarrhea after starting zinc supplementation

| | Experimental | | Control | | | Risk Ratio |
|---|---|---|---|---|---|---|
| Study or Subgroup | Events | Total | Events | Total | Weight | M-H, Fixed, 95% CI |
| Khatun 2001 (1) | 21 | 24 | 11 | 24 | 38.7% | 1.91 [1.20, 3.03] |
| Wang 2016 (2) | 28 | 31 | 18 | 33 | 61.3% | 1.66 [1.19, 2.31] |
| **Total (95% CI)** | | **55** | | **57** | **100.0%** | **1.75 [1.34, 2.30]** |
| Total events | 49 | | 29 | | | |

Heterogeneity: Chi² = 0.25, df = 1 (P = 0.62); I² = 0%

Test for overall effect: Z = 4.06 (P < 0.0001)

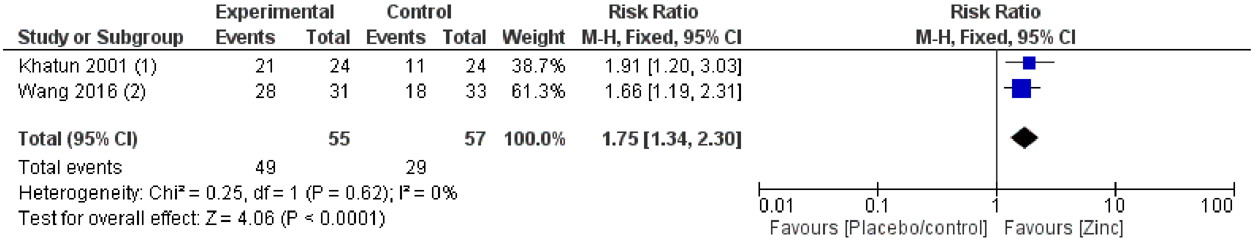


Footnotes
(1) 7 days after intervention
(2) 5 days after intervention

Figure 6

Outcome 2- Mortality

| Study or Subgroup | Experimental Events | Experimental Total | Control Events | Control Total | Weight | Risk Ratio M-H, Fixed, 95% CI |
|---|---|---|---|---|---|---|
| Khatun 2001 | 0 | 24 | 0 | 24 | | Not estimable |
| Roy 2007 (2) | 6 | 78 | 7 | 76 | 100.0% | 0.84 [0.29, 2.37] |
| **Total (95% CI)** | | **102** | | **100** | **100.0%** | **0.84 [0.29, 2.37]** |
| Total events | 6 | | 7 | | | |
| Heterogeneity: Not applicable | | | | | | |
| Test for overall effect: Z = 0.34 (P = 0.74) | | | | | | |

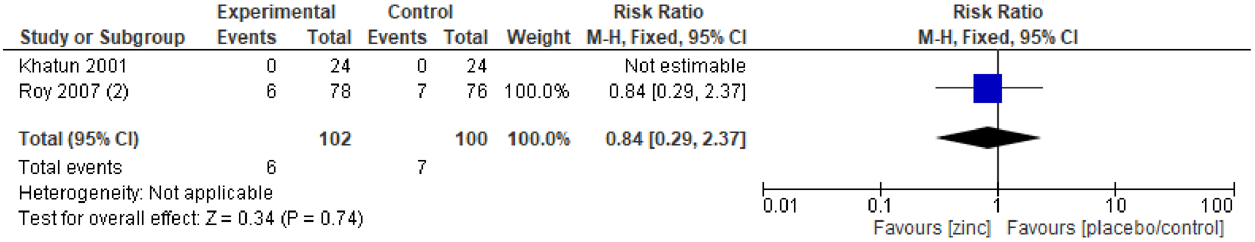


Figure 7

Outcome 3- Duration of diarrhea

| Study or Subgroup | Experimental Mean | Experimental SD | Experimental Total | Control Mean | Control SD | Control Total | Weight | Mean Difference IV, Random, 95% CI |
|---|---|---|---|---|---|---|---|---|
| Khatun 2001 | 69.6 | 33.6 | 44 | 84 | 33.6 | 44 | 44.9% | -14.40 [-28.44, -0.36] |
| Roy 2007 (2) | 45.6 | 9.6 | 78 | 81.6 | 14.88 | 76 | 55.1% | -36.00 [-39.97, -32.03] |
| **Total (95% CI)** | | | **122** | | | **120** | **100.0%** | **-26.29 [-47.35, -5.23]** |
| Heterogeneity: Tau² = 205.57; Chi² = 8.42, df = 1 (P = 0.004); I² = 88% | | | | | | | | |
| Test for overall effect: Z = 2.45 (P = 0.01) | | | | | | | | |

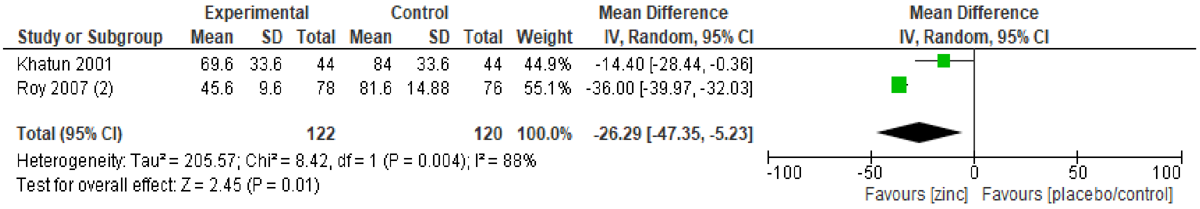


## **Low dose vs high dose zinc in acute diarrhea**

Figure 8- ROB 2 analysis for diarrhea greater than 5 days and vomiting

| Study ID | D1 | D2 | D3 | D4 | D5 | Overall |
|---|---|---|---|---|---|---|
| Dhingra 2020 | + | + | + | + | + | + |

## Figure 9- Forest Plot for Duration of Diarrhea More than Five Days

| Study or Subgroup | Experimental Events | Experimental Total | Control Events | Control Total | Weight | Risk Ratio M-H, Fixed, 95% CI |
|---|---|---|---|---|---|---|
| Dhingra 2020 (1) | 106 | 1480 | 48 | 739 | 50.0% | 1.10 [0.79, 1.53] |
| Dhingra 2020 (2) | 114 | 1480 | 48 | 740 | 50.0% | 1.19 [0.86, 1.64] |
| **Total (95% CI)** | | **2960** | | **1479** | **100.0%** | **1.15 [0.91, 1.44]** |
| Total events | 220 | | 96 | | | |

Heterogeneity: Chi² = 0.10, df = 1 (P = 0.75); I² = 0%
Test for overall effect: Z = 1.15 (P = 0.25)

Risk Ratio M-H, Fixed, 95% CI

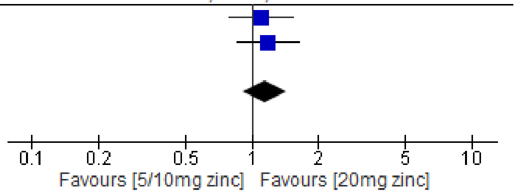


Footnotes
(1) 5mg
(2) 10mg

## Figure 10- Forest Plot for Frequency of Vomiting

| Study or Subgroup | Experimental Events | Experimental Total | Control Events | Control Total | Weight | Risk Ratio M-H, Fixed, 95% CI |
|---|---|---|---|---|---|---|
| Dhingra 2020 (1) | 301 | 1496 | 201 | 745 | 49.9% | 0.75 [0.64, 0.87] |
| Dhingra 2020 (2) | 333 | 1488 | 202 | 745 | 50.1% | 0.83 [0.71, 0.96] |
| **Total (95% CI)** | | **2984** | | **1490** | **100.0%** | **0.79 [0.70, 0.88]** |
| Total events | 634 | | 403 | | | |

Heterogeneity: Chi² = 0.84, df = 1 (P = 0.36); I² = 0%
Test for overall effect: Z = 4.37 (P < 0.0001)

Risk Ratio M-H, Fixed, 95% CI

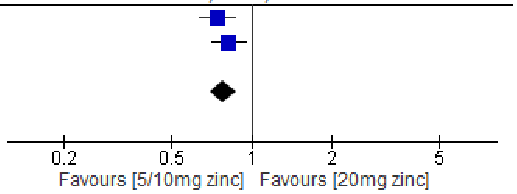


Footnotes
(1) 5mg
(2) 10mg
